# Chemically diverse activity-based probes with unexpected inhibitory mechanisms targeting trypsin-like serine proteases

**DOI:** 10.3389/fchem.2022.1089959

**Published:** 2023-01-05

**Authors:** Alba Ramos-Llorca, Lisse Decraecker, Valérie M. Y. Cacheux, Irena Zeiburlina, Michelle De bruyn, Louise Battut, Carlos Moreno-Cinos, Davide Ceradini, Eric Espinosa, Gilles Dietrich, Maya Berg, Ingrid De Meester, Pieter Van Der Veken, Guy Boeckxstaens, Anne-Marie Lambeir, Alexandre Denadai-Souza, Koen Augustyns

**Affiliations:** ^1^ Laboratory of Medicinal Chemistry, Department of Pharmaceutical Sciences, Faculty of Pharmaceutical, Biomedical and Veterinary Sciences, University of Antwerp, Antwerp, Belgium; ^2^ Laboratory for Intestinal Neuroimmune Interactions, Translational Research Center for Gastrointestinal Disorders, Department of Chronic Diseases, Metabolism and Ageing, KU Leuven, Leuven, Belgium; ^3^ Laboratory of Medical Biochemistry, Department of Pharmaceutical Sciences, Faculty of Pharmaceutical, Biomedical and Veterinary Sciences, University of Antwerp, Antwerp, Belgium; ^4^ IRSD, Université de Toulouse, INSERM, INRA, ENVT, UPS, Toulouse, France; ^5^ Latvian Institute of Organic Synthesis, Riga, Latvia

**Keywords:** activity-based probe, serine protease, inhibitors, mast cells (MCs), irreversible inhibition

## Abstract

Activity-based probes (ABP) are molecules that bind covalently to the active form of an enzyme family, making them an attractive tool for target and biomarker identification and drug discovery. The present study describes the synthesis and biochemical characterization of novel activity-based probes targeting trypsin-like serine proteases. We developed an extensive library of activity-based probes with “clickable” affinity tags and a diaryl phosphonate warhead. A wide diversity was achieved by including natural amino acid analogs as well as basic polar residues as side chains. A detailed enzymatic characterization was performed in a panel of trypsin-like serine proteases. Their inhibitory potencies and kinetic profile were examined, and their IC_50_ values, mechanism of inhibition, and kinetic constants were determined. The activity-based probes with a benzyl guanidine side chain showed the highest inhibitory effects in the panel. Surprisingly, some of the high-affinity probes presented a reversible inhibitory mechanism. On the other hand, probes with different side chains exhibited the expected irreversible mechanism. For the first time, we demonstrate that not only irreversible probes but also reversible probes can tightly label recombinant proteases and proteases released from human mast cells. Even under denaturing SDS-PAGE conditions, reversible slow-tight-binding probes can label proteases due to the formation of high-affinity complexes and slow dissociation rates. This unexpected finding will transform the view on the required irreversible nature of activity-based probes. The diversity of this library of activity-based probes combined with a detailed enzyme kinetic characterization will advance their applications in proteomic studies and drug discovery.

## 1 Introduction

In recent years, many efforts have been carried out to study the expression and function of proteins in biological organisms. This provided new insights into the pathophysiology of different diseases and disorders. Activity-based protein profiling (ABPP) is a proteomics technique that uses activity-based probes (ABPs) to visualize and characterize enzyme activity within a complex proteome. These chemical probes are designed to react covalently with the active form of a target enzyme and allow their detection or isolation. Thus, ABPs give information about the activity level of an enzyme rather than its expression level (Cravatt et al., 2008; Fang et al., 2021). This feature represents a considerable advantage since protease activity is tightly regulated. All ABPs share a similar structure consisting of four parts: 1) a warhead or reactive group; 2) a selectivity enhancing group that targets a specific enzyme family; 3) a reporter tag used for visualization or isolation, and 4) a linker or spacer to connect the three other components (Jessani and Cravatt, 2004; Berger et al., 2012).

ABPs have the potential for target and biomarker identification in different pathologies (Carvalho et al., 2015). The most common application for ABPs is detecting and visualizing active enzymes in biological samples, including *in vitro* samples, and visualization in animal models *in vivo*. However, the uptake of the ABPs by living organisms can be problematic due to their bulkiness. Alternatively, conjugation with the reporter tag can be done *in situ* by click chemistry. The probe precursors are added to living cells or tissues and then undergo the reaction with a fluorophore or affinity label to yield the ABP. Subsequently, the probes can be visualized or identified by different techniques (Sanman and Bogyo, 2014; Yao et al., 2021).

Different analytical tools can detect the complex enzyme-ABP. The method used depends on the reporter tag of choice. Fluorescent or radioactive labels could be detected by SDS-PAGE gel, imaging by microscopy, or flow cytometry (Herrera Moro Chao et al., 2015; Poreba et al., 2018; Elvas et al., 2019). Whereas, Western blots are used for biotinylated probes (Sadaghiani et al., 2007; Edgington et al., 2011). The latter can also be used to isolate or purify the targeted enzyme before detection. The strong interaction of biotin with immobilized avidin makes it a good strategy for enzyme affinity purification and mass spectrometry (MS) detection, thereby allowing the unambiguous identification of active proteases (Fonović and Bogyo, 2008; Li et al., 2013). Unfortunately, the conditions needed to dissociate the biotin-avidin interaction are harsh and sometimes unsuitable for the analysis. Alternatively, desthiobiotin, an analog of biotin which does not contain sulfur, can be used. Desthiobiotin has a lower affinity with avidin, therefore, the ABPs and enzymes can better tolerate the dissociation conditions (Hirsch et al., 2002; Ngo et al., 2019).

ABPs have been designed for various enzyme families (Yee et al., 2005; Kalesh et al., 2010; Kallemeijn et al., 2012). Specifically, they have been widely studied for many proteases (Serim et al., 2012), such as cysteine (Wang et al., 2003; Kato et al., 2005), metalloproteases (Geurink et al., 2010) and serine proteases (Shannon et al., 2012). Serine proteases represent one of the largest classes of proteases expressed in the human degradome. Unlike cysteine proteases, which are predominantly intracellular, most serine proteases are secreted enzymes, thereby active players in cell-to-cell communication in health and disease. Unambiguous identification of active serine proteases released by cells has been hindered by the limitations of classical studies based on enzymatic substrates and inhibitors due to the catalytic overlap among proteases, while the use of ABPs for this purpose has made its proof (Denadai-Souza et al., 2018).

The first probe targeting serine proteases was based on a fluorophosphonate warhead, designed to react covalently with the active site serine residue on serine hydrolases. However, it has a wide-ranging reactivity and did not show selectivity towards serine proteases (Liu et al., 1999). Other warheads reported for this enzyme family include isocoumarins (Kam et al., 1993), sulfonyloxyphtalimides (Schulz-Fincke et al., 2018), and diphenyl phosphonates (Ides et al., 2014).

Diphenyl phosphonate (DPP) is a well-studied reactive group. The phosphorus can react with the serine hydroxyl on the active site to form a covalent bond. Thus, it has the required characteristics to design selective and irreversible ABPs. Therefore, it was previously used to develop irreversible inhibitors and ABPs targeting serine proteases (Oleksyszyn and Powers, 1991; Jackson et al., 1998; Joossens et al., 2007).

Within the human degradome, serine proteases can be divided into three main subfamilies known as trypsin-, chymotrypsin-, and elastase-like, depending on their substrate specificity. The specificity of a protease is determined by the preferred amino acid sequence that will fit into the specificity pocket of the active site (Perona and Craik, 2008). In this study, we focus on trypsin-like serine proteases. These have an aspartic acid residue at the bottom of the primary substrate binding pocket (S1). Thus their preference is shifted towards basic and polar residues such as lysine or arginine (Drag and Salvesen, 2010).

**FIGURE 1:**
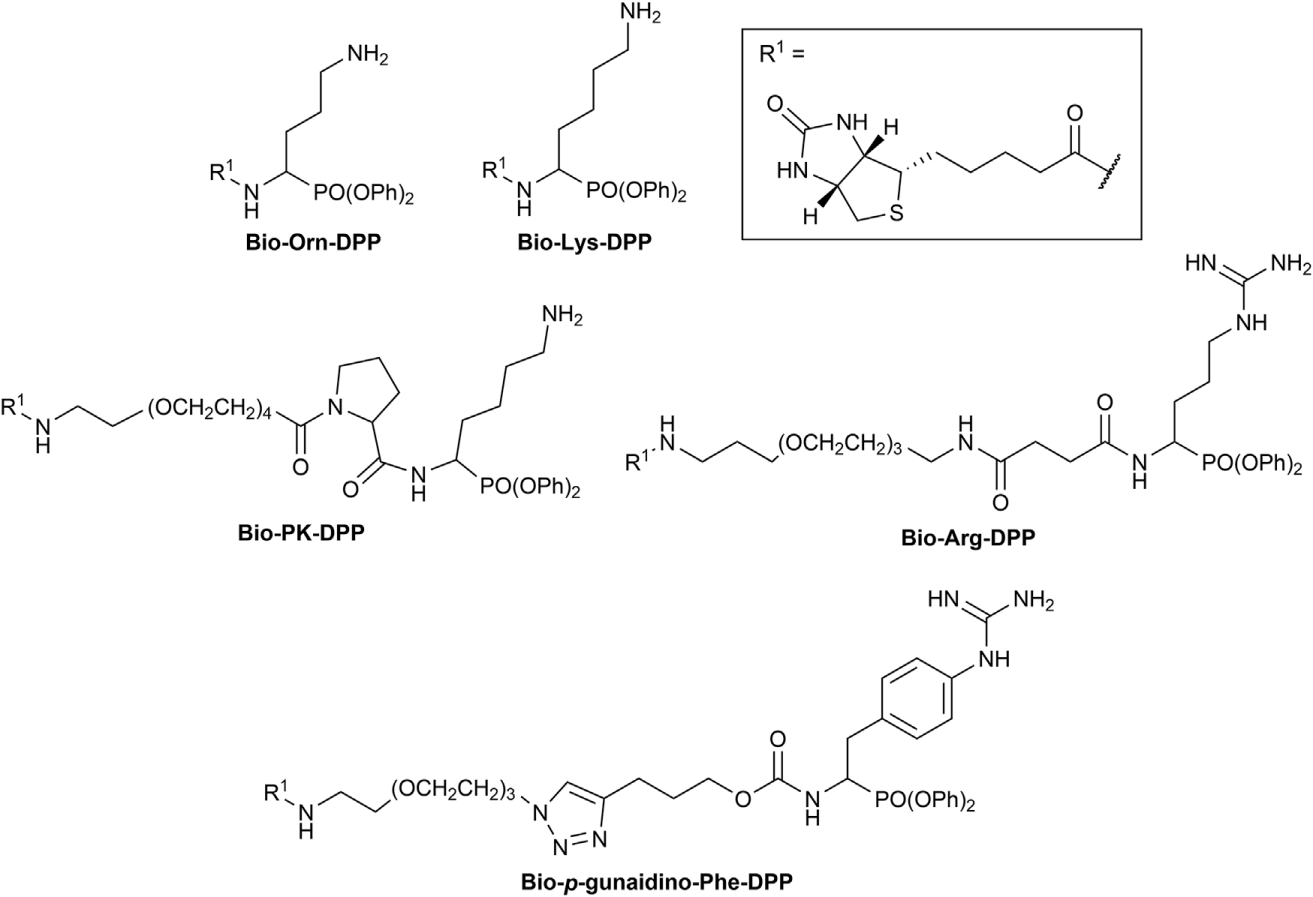
Chemical structure of published biotinylated ABPs targeting trypsin-like serine proteases.

The first synthesized *a*-aminoalkyl diphenyl phosphonate ABPs targeting trypsin-like proteases were described by Hawthorne et al. (2004). These were based on lysine (Biotin-Lys-DPP) and ornithine (Bio-Orn-DPP) as a selectivity enhancing group. Similarly, Pan et al. (2006) described a lysine ABP by adding a pegylated linker. Additionally, they described a promising ABP with two residues, lysine in P1 and proline in P2 (Biotin-PK-DPP). More recently, Reihill et al. described an arginine probe (Biotin-Arg-DPP) as an inhibitor for channel activating proteases (Martin and Walker, 2011; Reihill et al., 2016; Ferguson et al., 2022). Moreover, several “clickable” benzyl guanidine probes conjugated with different reporter tags were reported, for instance, biotin (Biotin-*p*-guanidino-Phe-DPP) (Augustyns et al., 2012). This was proven to be a potential Positron emission tomography (PET) imaging agent if coupled with a radioactive label (Ides et al., 2014) (Figure 1).

Driven by the need to identify new biomarkers in pathologies where serine proteases are upregulated and inspired by previously reported irreversible serine protease inhibitors and ABPs, this study reports the synthesis and characterization of a library of ABPs for trypsin-like serine proteases. The idea was to move away from the ABPs mimicking the natural basic amino acids lysine and arginine and to design several different synthetic analogs to improve potency and selectivity. The ABPs described are based on a DPP warhead with a biotin or desthiobiotin tag attached by click chemistry. We performed an extensive biochemical characterization and demonstrated the ability of these ABPs to differently label trypsin-like serine proteases, both recombinant enzymes and also from degranulated mast cells. Thus, these ABPs have the potential to be used in more complex pathological samples to identify active trypsin-like proteases participating in physiological processes as well as dysregulated enzymes potentially promoting disease. Therefore, these could represent a valuable tool with translational prospects for target identification and biomarker discovery.

## 2 Materials and methods

### 2.1 Chemistry

Reagents were obtained from commercial sources and were used without further purification. Characterization of all compounds was done with ^1^H and ^13^C NMR and mass spectrometry. ^1^H and ^13^C NMR spectra were recorded on a 400 MHz Bruker Avance III Nanobay spectrometer with Ultrashield working at 400 and 100 MHz, respectively, and analyzed by use of MestReNova or TopSpin analytical chemistry software. Chemical shifts (δ) are in parts per million (ppm), and coupling constants (*J*) are in hertz (Hz). The signal splitting patterns were described as s = singlet, d = doublet, t = triplet, q = quartet, p = pentuplet, dd = doublet of doublet, dt = doublet of triplet, td = triplet of doublet, tt = triplet of triplet, ddd = doublet of doublet of doublet, br = broad, and m = multiplet. The LC−MS analysis was performed on a Waters UPLC−MS system equipped with a TUV and QDa detector; the column used is an Acquity UPLC BEH C18 (1.7 μm, 2.1 × 50 mm), and as eluent, a mixture of .1% FA in H_2_O, .1% FA in CH3CN, H_2_O, and MeCN. The wavelengths for UV detection were 254 and 214 nm.

When necessary, flash chromatography separations were carried out using a Biotage Isolera One purification system equipped with an internal variable dual-wavelength diode array detector (200–400 nm). Silica gel columns were used for normal phase purifications, and reverse-phase purifications were done using C18 cartridges, both from Büchi or Biotage. Dry sample loading was done by self-packing sample cartridges using Celite 545. Gradients used varied for each purification.

HRMS involved the following: the dry samples of final compounds were dissolved in CH_3_CN to a concentration of .1 mM and then diluted x10 in a solution 50:50 of CH_3_CN:H_2_O.

The synthetic procedures and analytical data for all compounds reported in this manuscript can be found in the Supplementary Material. Additional data, including schemes for the synthesis of intermediates en route to final compounds, can also be found in the Supplementary Material.

### 2.2 Determination of IC_50_ values

The IC_50_ value is defined as the concentration of inhibitor required to reduce the enzyme activity to 50% after a 15 min preincubation with the enzyme at 37°C before the addition of the substrate. The IC_50_ values were determined using a spectrophotometric assay. All the experiments were conducted in duplicate. The readout consisted of evaluating the protease-mediated release of the chromophore *para*-nitroaniline (pNA) or fluorophore 7-amino-4-methyl coumarin (AMC) moieties from the respective substrates. Enzymatic activity was measured for 30 min at 37°C. All compounds were initially screened at three concentrations (10, 1, and .1 μM) to estimate the range of the IC_50_ value. Those which were able to reduce protease activity by at least 50% at a concentration of 10 μM were submitted to an exact IC_50_ determination. The final IC_50_ values of the most potent inhibitors were the average of two independent experimental results. A third independent experiment was performed when the standard deviation of the two independent experiments was higher than three times the IC_50_ value. IC_50_ values were obtained by fitting the data with the four-parameter logistics equation using GraphPad Prism 9.

The conditions for each protease are described as follows, the concentration of substrate used was at the corresponding K_m_.

Trypsin-3 (recombinant trypsin-3, R&D) and fluorogenic substrate tosyl-Gly-Pro-Arg-AMC (K_m_ = 22.5 μM) were used in Tris buffer (100 mM Tris, 1 mM CaCl_2_) at pH 8.0 (25°C). Tryptase (recombinant tryptase β-2, EnzoLife Sciences) and fluorogenic substrate Boc-Gln-Ala-Arg-AMC (K_m_ = 250 μM) were used in Tris buffer (50 mM Tris, 120 mM NaCl, .1 mg/ml BSA, .1 mg/ml heparin) at pH 8.0 (25°C). Thrombin (recombinant thrombin, R&D) and fluorogenic substrate Boc-Val-Pro-Arg-AMC (K_m_ = 15 μM) were used in TRIS buffer (50 mM Tris HCl, 50 mM Tris base, 10 mM CaCl_2_, 150 mM NaCl) at pH 8.3 (25°C). uPA (recombinant urokinase plasminogen activator, HYPHEN BioMed) and chromogenic substrate *pyro*-Glu-Gly-Arg-pNA (K_m_ = 80 μM) were used in HEPES buffer (50 mM HEPES) at pH 8.1 (25°C). Cathepsin G (cathepsin G human neutrophil, Sigma-Aldrich) and fluorogenic substrate Suc-Ala-Ala-Pro-Phe-AMC (K_m_ = 130 μM) were used in Tris buffer (50 mM Tris, 120 mM NaCl) at pH 8.0 (25°C). TLCK-treated pancreatic bovine chymotrypsin (Sigma-Aldrich) and fluorescent substrate Suc-Ala-Ala-Pro-Phe-AMC (K_m_ = 58 μM) were used in Tris buffer (50 mM Tris, 20 mM CaCl_2_) at pH 8.3 (25°C). Neutrophil Elastase (recombinant neutrophil elastase, Enzo LifeSciences) and fluorogenic substrate Suc-Ala-Ala-Pro-Val-AMC (K_m_ = 500 μM) were used in Tris buffer (50 mM Tris, 120 mM NaCl) at pH 8.0 (25°C).

### 2.3 Kinetic experiments and determination of k_app_, K1 and K_i_^*^

Because the compounds described in this paper were previously described as irreversible inhibitors, the IC_50_ value is inversely correlated with the second-order rate constant of inactivation. For a simple pseudo-first-order inactivation process, the activity after incubation with inhibitor (v_i_) varies with the inhibitor concentration (i), as described in the following equation:

$$v_{i}=v_{0}\cdot e^{-kit}$$

where v_0_ is the activity in the absence of an inhibitor, *k* is the second-order rate constant of inactivation, and *t* is the time. The inactivation rate constant was determined from the time course of inhibition.

Kinetic assays were performed in the following manner. The inhibitor was mixed with the substrate, and the buffer solution with the enzyme was added at the time zero. The progress curves show the release of pNA or AMC as a function of time. Initially, no inhibitor is bound to the enzyme, and the tangent to the progress curve (dA/dt) is proportional to the free enzyme concentration. The free enzyme concentration decreases over time due to inhibitor binding kinetics, as described above. Progress curves were recorded in pseudo-first-order conditions ([I]_0_>>[E]_0_) and with less than 10% conversion of the substrate during the entire time course. In these conditions, dA/dt decreases exponentially with time. The progress curves were fitted with the integrated rate equation with GraFit7 to yield a value for k_obs_, a pseudo-first-order rate constant.

$$A_{t}=A_{o}+v_{s}∙t+\left(v_{i}-v_{s}\right)∙\left(1-e^{-kobs∙t}\right)/k_{obs}$$

where A_t_ is the absorbance at time t, A_0_ is the absorbance at time zero, v_i_ is the initial rate and v_s_ is the velocity at the steady-state.

The apparent second-order rate constant (k_app_) was calculated from the slope of the linear part of the plot of k_obs_ vs. the inhibitor concentration ([I]_0_). In the case of competition between the inhibitor and the substrate, k_app_ is smaller than the “real” second-order rate constant k discussed above because a certain fraction of the enzyme is present as an enzyme-substrate complex. k_app_ depends on the substrate concentration used in the experiment, as described by Lambeir et al. (1996).

The apparent equilibrium constants were obtained from the following equations,

$$\frac{v_{i}}{v_{o}}=\frac{1}{1+\frac{\left[I\right]}{K1}}\;;\;\frac{v_{S}}{v_{o}}=\frac{1}{1+\frac{\left[I\right]}{K_{I}^{*}}}$$

where v_i_ is the initial rate, v_s_ is the steady-state velocity and v_o_ is the uninhibited initial rate.

### 2.4 Determination of inhibition type

To monitor the dissociation of the inhibitor-enzyme, aliquots of the enzyme were incubated at 37°C without and with the inhibitor at a concentration 10 times higher than its IC_50_. The enzyme concentration was 2.5 times higher than the concentration used for the IC_50_ determinations. After 15 min, the aliquots were diluted 10-fold or 100-fold into the substrate concentration and assay buffer used for the IC_50_ determination. The dissociation of the enzyme-inhibitor complex was monitored by substrate hydrolysis over time (Joossens et al., 2006).

### 2.5 Labeling and detection of recombinant proteases

Recombinant proteases (100 ng) were labeled with 1 µM ABP in reaction buffer in a final volume of 40 µL for 30 min at 37°C. After the addition of 4X Laemmli buffer (Bio-Rad, GmbH) supplemented with Bond-Breaker Tris (2-CarboxyEthyl)Phosphine (TCEP) Solution (Thermo Scientific, United States), the samples were heated at 95°C for 5 min and loaded into 4%–20% Mini-Protean TGX precast gels (Bio-Rad, GmbH). After electrophoresis, the proteins were blotted onto nitrocellulose membranes using the Trans-Blot Transfer Turbo System (Bio-Rad). Membranes were incubated with NeutrAvidin-HRP (1:50,000), and bands were visualized with ECL Select Western Blot Detection Reagent (GE Healthcare Life Sciences) by chemiluminescence (Chemidoc XRS; Bio-Rad). The molecular weight of each band was determined with the Image Lab Software v5 (Bio-Rad).

### 2.6 Primary human mast cells culture

Peripheral blood mononuclear cells (PBMCs) were obtained from buffy coats (Etablissement Français du Sang). CD34^+^ precursors cells were isolated from PBMCs (EasySep™ Human CD34 Positive Selection Kit, STEMCELL Technologies) and grown under serum-free conditions using StemSpanTM medium (STEMCELL Technologies) supplemented with recombinant human IL-6 (50 ng/ml; Peprotech), human IL-3 (10 ng/ml; Peprotech) and 3% supernatant of CHO transfectants secreting murine SCF (a gift from Dr. P. Dubreuil, Marseille, France, 3% correspond to ∼50 ng/ml SCF) for 1 week. Cells were next grown in IMDM Glutamax I, sodium pyruvate, 2-mercaptoethanol, .5% BSA, Insulin-transferrin selenium (all from Invitrogen), penicillin (100 U/ml), streptomycin (100 μg/ml) and 3% supernatant of CHO transfectants secreting murine SCF for 8 weeks then tested phenotypically (CD117+, FcεRI+) and functionally (β-hexosaminidase release in response to FcεRI crosslinking) before use for experiments. Only primary cell lines showing more than 95% CD117+/FcεRI+ cells were used for experiments.

### 2.7 Mast cell degranulation assay

1 × 10^5^ mast cells were distributed in 50 µl Tyrode’s Buffer and adapted to 37°C for 30 min. Mast cells were next stimulated with DNP-BSA (200 ng/ml) after sensitization with 1 μg/ml of IgE anti DNP (Sigma-Aldrich) overnight. Supernatants were harvested and stored at −80°C. (β-hexosaminidase was assayed by measuring the release of p-nitrophenol from the substrate p-nitrophenyl N-acetyl-β-D-glucosaminide).

### 2.8 Measurement of protein concentration

The concentration of protein in supernatants was determined using the Pierce Protein BCA Assay Kit, according to instructions (Thermo Scientific).

### 2.9 Measurement of proteolytic activity

Tryptase-like activity in mast cell supernatants was measured with .075 mM Boc-Gln-Ala-Arg-AMC hydrochloride as substrate in 50 mM Tris 120 mM NaCl + .1 mg/ml BSA + 50 μg/ml heparin pH = 8 at 25°C (Sigma-Aldrich). Substrate cleavage was calculated by the change in fluorescence (excitation: 355 nm, emission: 460 nm), measured over 30 min at 37 °C on a FLUOstar Omega microplate reader (BMG Labtech). Sample values were interpolated into a linear regression generated with a standard curve of AMC (Sigma-Aldrich). Data were expressed as U of tryptase-like activity per L (U defined as 1 μmol/min).

### 2.10 Functional proteomic profiling of mast cell supernatants

Mast cell supernatants (10 µg of total protein) were labeled with 1 µM individual ABPs (46b, 52b, or 57b) in 50 mM Tris, 120 mM NaCl, .1 mg/ml BSA, 50 μg/ml heparin, pH 8 at 25°C (Sigma-Aldrich) in a final volume of 500 µL. As a control, supernatants were pre-incubated for 60 min at 37°C under stirring (1,000 rpm) in 4 mM AEBSF (Sigma), and 1X complete EDTA-free inhibitor cocktail (Roche) before incubation with a mixture containing the 3 ABPs mentioned above. The pre-incubation with protease inhibitors allows the identification of active proteases since enzyme inhibition abrogates their interaction with the ABP. The reaction product was then precipitated in 15% trichloroacetic acid at 4°C overnight. The pellet was washed twice in cold acetone (−20°C) and solubilized in 45 μL of Laemmli buffer (Bio-Rad) supplemented with Bond-Breaker TCEP Solution (Thermo Scientific). Samples were then heated at 95°C for 5 min, clarified by centrifugation at 12,000 *g* for 5 min, and the solubilized sample was loaded into 4%–20% Mini-Protean TGX precast gels (Bio-Rad, GmbH). After electrophoresis, the proteins were blotted onto nitrocellulose membranes using the Trans-Blot Transfer Turbo System (Bio-Rad). Membranes were incubated with NeutrAvidin-HRP, and bands were visualized with ECL Select Western Blot Detection Reagent (GE Healthcare Life Sciences) by chemiluminescence (Chemidoc XRS; Bio-Rad). The molecular weight of each band was determined with the Image Lab Software v5 (Bio-Rad) with Dual Color Protein Precision Plus Standard (10–250 kDa; Bio-Rad) as reference. The bands corresponding to active proteases were identified by their sensitivity to the pre-incubation with protease inhibitors.

### 2.11 Detection of tryptase in mast cell supernatants

Protein in the mast cell supernatants (75 µg of total protein) was precipitated in 15% trichloroacetic acid at 4°C overnight. The pellet was washed twice in cold acetone (−20°C) and solubilized in 75 μL of Laemmli buffer protein supplemented with TCEP (Bio-Rad). Samples were then heated at 95°C for 5 min, clarified by centrifugation at 12,000 *g* for 5 min, and 5 µg of the solubilized sample was loaded into 4%–20% Mini-Protean TGX precast gels (Bio-Rad, GmbH). After electrophoresis, the proteins were blotted onto a nitrocellulose membrane using the Trans-Blot Transfer Turbo System (Bio-Rad). The membrane was incubated with primary anti-mast cell tryptase antibody (sc-32889, Santa Cruz Biotechnology) at 1:1,000 dilution overnight at 4°C. Subsequently, the membrane was labeled with the secondary HRP-conjugated antibody (1:10.000; P0448, Dako) for 1 h at room temperature. Bands were visualized with ECL Select Western Blot Detection Reagent (GE Healthcare Life Sciences) by chemiluminescence (Chemidoc XRS; Bio-Rad). The molecular weight of each band was determined with the Image Lab Software v5 (Bio-Rad).

## 3 Results

### 3.1 Chemical synthesis

With the aim to achieve a range of chemical tools able to identify different trypsin-like serine proteases in complex proteomes, the first goal of this study was to obtain an extensive and diverse library of ABPs. Trypsin-like serine proteases interact favorably with basic and polar residues in the S1 pocket (Perona and Craik, 2008). Previously reported diaryl phosphonate ABPs targeting this family incorporated ornithine and the trypsin-like natural substrate, lysine (Pan et al., 2006). Other studies included benzyl guanidine as the selectivity enhancing moiety (Ides et al., 2014). Pan et al. (2006) described a promising ABP for trypsin with a two amino acid diphenyl phosphonate with proline and lysine. To broaden the probe library, not only amino acids lysine and arginine were included but also other non-natural amino acid analogs with different polar and basic moieties and a two amino acid probe with proline and lysine. In addition to residues targeting the trypsin-like family, phenylalanine and valine were introduced to target chymotrypsin and elastase-like serine proteases.

**FIGURE 2:**
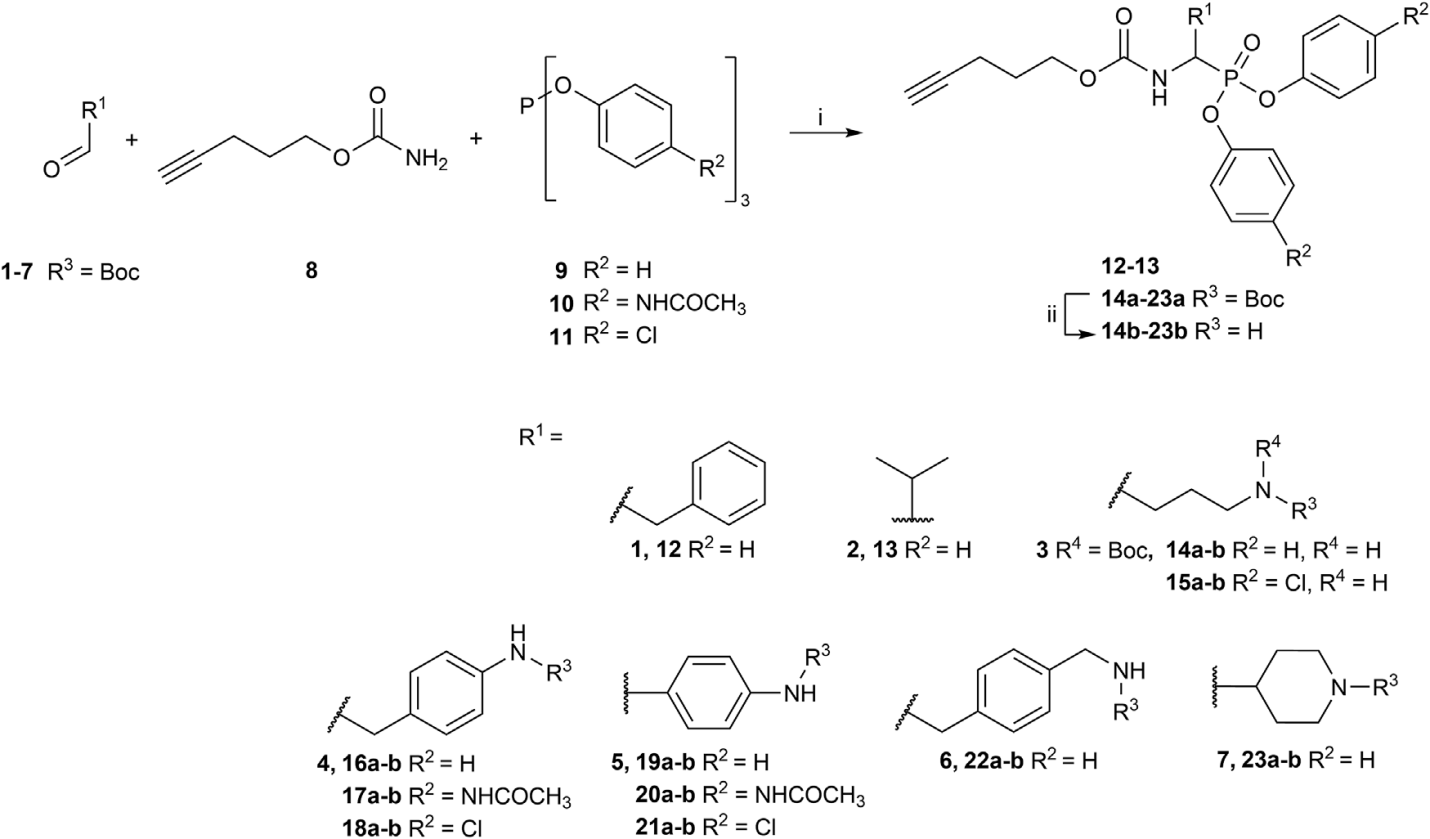
General synthesis of diaryl phosphonate alkyne probes by the Birum-Oleksyszyn reaction; (i) Lewis acid, solvent, rt, 16 h; (ii) TFA, DCM, rt, 2–5 h or HCl (4M, dioxane), rt, 2–5 h.

Although diaryl phosphonate ABPs targeting serine proteases have been reported in the literature, this study aimed to develop a common and accessible synthetic strategy for a wide-ranging library. Therefore, instead of adding the reporter tag by commonly used peptidic coupling (Pan et al., 2006; Reihill et al., 2016), the pegylated linker was added by click-chemistry. More specifically, the Huisgen 1,3-dipolar cycloaddition between an azide and an alkyne to form a 1,2,3-triazole moiety (Breugst and Reissig, 2020). This approach requires an initial probe bearing the warhead with a terminal alkyne. The synthetic strategy depicted in Figure 2 was inspired by the previously described route used for benzyl guanidine ABPs synthesis (Augustyns et al., 2012; Ides et al., 2014).

The primary step of the diaryl phosphonate probe synthesis is the Birum-Oleksyszyn reaction, first described by Oleksyszyn et al. (1979) in 1979 and later optimized by van der Veken et al. (2004) in 2005. This is a three-component reaction involving an aldehyde, a carbamate, and a triaryl phosphite, catalyzed by an acid (Figure 2). The product can contain an acid-labile Boc-protecting group if a Lewis acid is used as the catalyst instead of a Brønstead acid. The aldehyde carries the selectivity enhancing group targeting the enzyme S1 pocket. Thus, various aldehydes were used to extend the chemical space (1–7). The pent-4-yn-1-yl carbamate (8) was prepared from the corresponding alcohol to conjugate the precursor with the reporting tag (Supplementary Figure S1). To add further diversity, triaryl phosphites (9–11) with two different substituents in the *para-*position were prepared from phosphorus trihalides (Supplementary Figures S2, S3).

The Birum-Oleksyszyn step required protected nitrogen aldehydes. Thus, for most products, the commercial alcohols were first protected with a Boc group, followed by Dess Martin periodinane (DMP) oxidation to achieve the desired aldehyde (4–5, 7) (Supplementary Figure S4). However, other protecting or oxidation strategies were needed for the aliphatic amines and the benzyl methyl amine.

**FIGURE 3:**
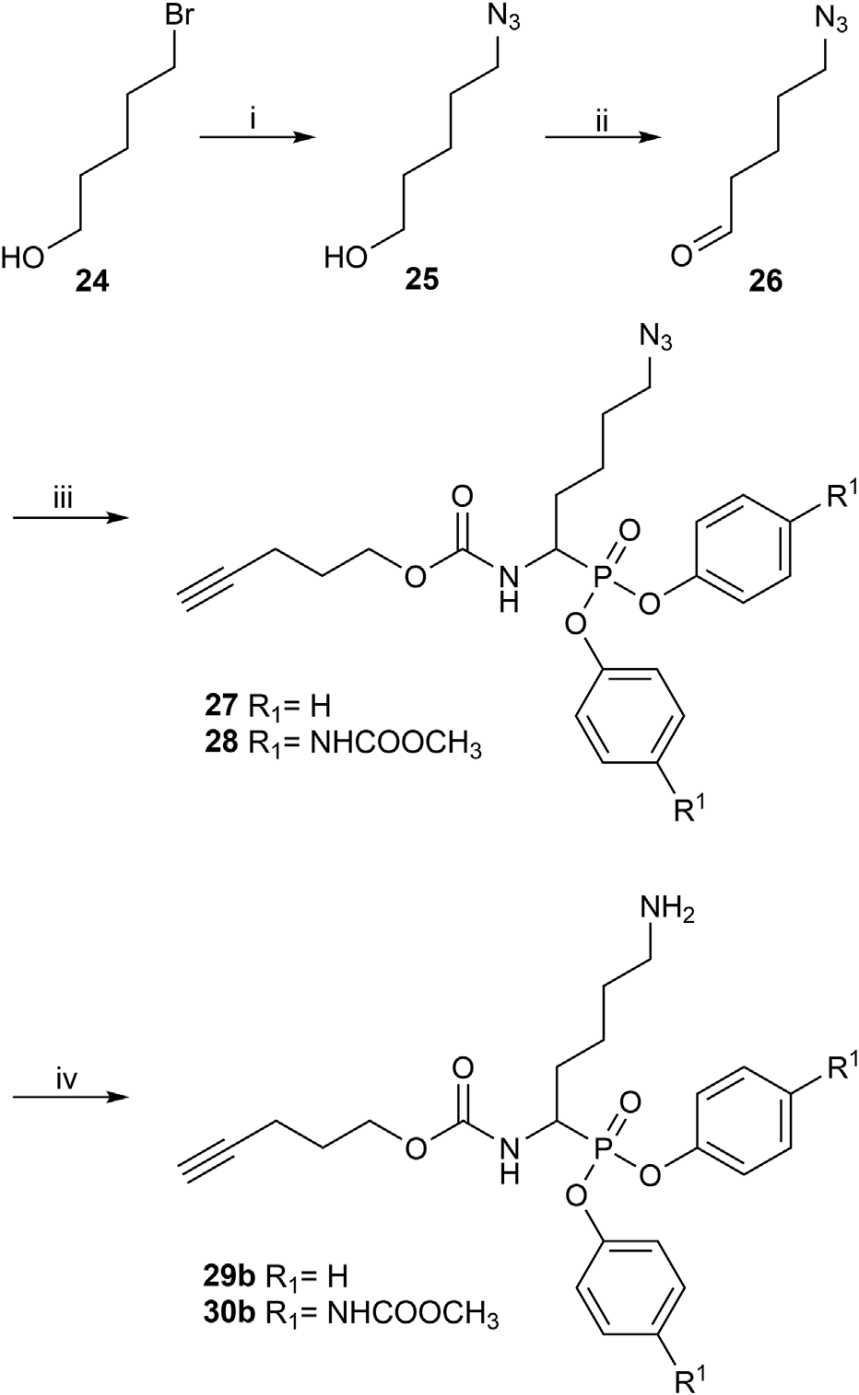
Synthesis of alkyne probes with Lysine as selectivity enhancing moiety (i) NaN_3_, H_2_O, 80°C, 18 h; (ii) DMP, DCM, 0°C to rt, 4 h; (iii) phosphite 9 or 10, carbamate 8, Lewis acid, DCM, rt, 16 h; (iv) Polymer supported triphenylphosphine, THF, rt, 72 h.

The synthesis of 4-aminobutanal (3) required a double Boc protection of the corresponding alcohol before a Swern oxidation (Supplementary Figure S5). In contrast, the double Boc protection of 5-aminopentanal was insufficient to achieve yields higher than 5% in the Birum-Oleksyszyn reaction. It is suggested that the catalytic quantity of Lewis acid is enough to deprotect one of the Boc groups. Since Boc protection was not adequate, other synthetic strategies were assessed. The described synthesis of a diphenyl phosphonate probe with lysine and proline-lysine by Pan et al. (2006) was achieved by phthalimide protection. Unfortunately, in our hands, the phosphonate was readily hydrolyzed when deprotecting with hydrazine. A study by Hamilton et al. (1993) also postulated that they experienced inconsistency with hydrazine-mediated phthalimide deprotection. Therefore, we explored a novel synthetic strategy with the use of an azide group instead of the primary amine. Hence, the desired lysine alkyne probes (29b–30b) were obtained from 5-azidopentanal (26), and the further reduction to a primary amine was best achieved with polymer-supported triphenylphosphine in THF (Figure 3). The same synthetic strategy was used for the proline-lysine alkyne probe (31b). The proline and the alkyne were introduced by peptidic coupling (Supplementary Figure S6). A similar approach was used to obtain the benzylmethylamine aldehyde (6), as the corresponding alcohol was not commercially available (Supplementary Figure S7).

The Boc protecting groups of the diarylphosphonates were removed using acid treatment with hydrogen chloride in dioxane or trifluoroacetic acid in dichloromethane (DCM).

The previously reported synthesis of benzylamine alkyne probes 16 and 17 used Cu(OTf)_2_ as a catalyst in DCM and tetrahydrofuran (THF), respectively. However, for the synthesis of benzylamine 4-acetamidophendiphenyl phosphonate (17), low yields were reported (<5%) (Augustyns et al., 2012). For the synthesis of this library, these catalyst and solvent combinations were kept. However, other Lewis acids and solvents were assessed when low yielding. A summary of the conditions used is shown in Table 1. For example, for the synthesis of 17, the yield was increased from 5% to 19% when using BF_3_(OEt)_2_ in acetonitrile (CH_3_CN). For the benzylamine alkyne probe with unsubstituted diphenyl phosphonate (16), the combination of Cu(OTf)_2_ in DCM worked in a similar yield range as reported (Augustyns et al., 2012). These conditions were used for all the unsubstituted products (*R* ^2^ = H). However, for substituted diaryl phosphonates, either Bi(OTf)_3_ in THF (15), BF_3_(OEt)_2_ in CH_3_CN (17–18, 28), or Cu(OTf)_2_ in THF (20–21) were more successful. Interestingly, Bi(OTf)_3_ has recently been reported as one of the best-performing catalysts for the Birum-Oleksyszyn reaction in an extensive optimization of the synthesis of inhibitor UAMC-00050 (Joossens et al., 2007; Ceradini and Shubin, 2021).

**TABLE 1:** Birum-Oleksyszyn reaction conditions.

#	R^1^	R^2^	Lewis acid	Solvent	Yield, %^a^
12	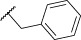	H	Cu(OTf)_2_	DCM	62
13	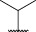	H	Cu(OTf)_2_	DCM	55
14	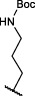	H	Cu(OTf)_2_	DCM	5
15		Cl	Bi(OTf)_3_	THF	10
16	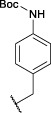	H	Cu(OTf)_2_	DCM	29
17		NHCOCH_3_	BF_3_(OEt)_2_	CH_3_CN	19
18		Cl	BF_3_(OEt)_2_	CH_3_CN	10
19	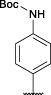	H	Cu(OTf)_2_	DCM	50
20		NHCOCH_3_	Cu(OTf)_2_	THF	34
21		Cl	Cu(OTf)_2_	THF	41
22	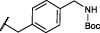	H	Cu(OTf)_2_	DCM	45
23	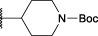	H	Cu(OTf)_2_	DCM	46
27	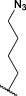	H	Cu(OTf)_2_	DCM	71
28		NHCOCH_3_	BF_3_(OEt)_2_	CH_3_CN	59

^a^ Isolated yield.

**FIGURE 4:**
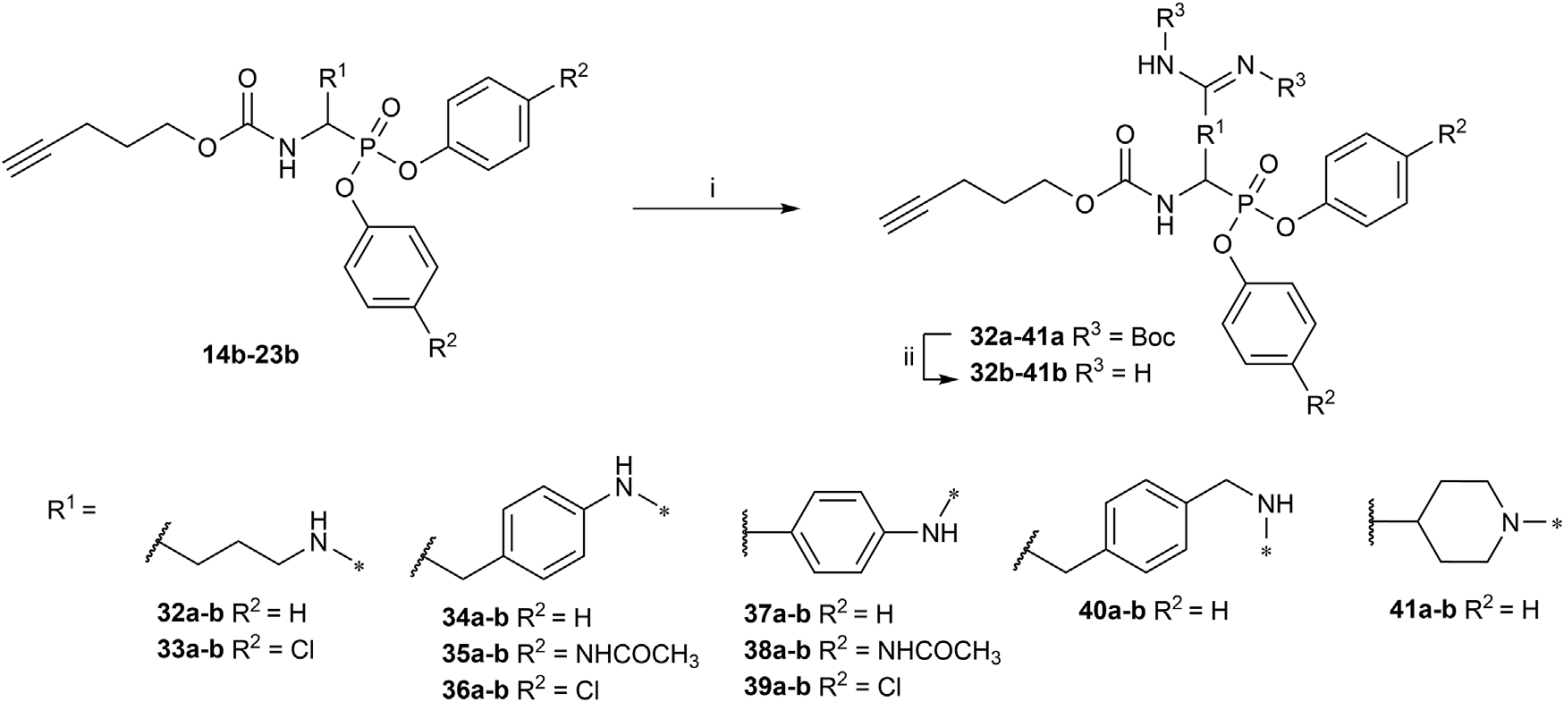
Guanylation of alkyne probes; (i) N, N′-Bis-Boc-1-Guanylpyrazole, Et_3_N, DCM/CH_3_CN (1:1), rt, 16 h; (ii) TFA, DCM, rt, 2–5 h or HCl (4M, dioxane), rt, 2–5 h.

All the alkyne probes with a basic amine, except all the lysine analogs, were elongated for further chemical space exploration by adding a guanidine moiety. The protected guanidine was inserted with *N, N′*-bis(*tert*-butoxycarbonyl)-1-guanylpyrazole in a mixture of 1:1 of CH_3_CN/DCM, reducing the previously reported reaction time from 3 days to 16 h (Augustyns et al., 2012). Later, the guanidine was deprotected by acid treatment before biochemical characterization (32b–41b) (Figure 4).

**FIGURE 5:**
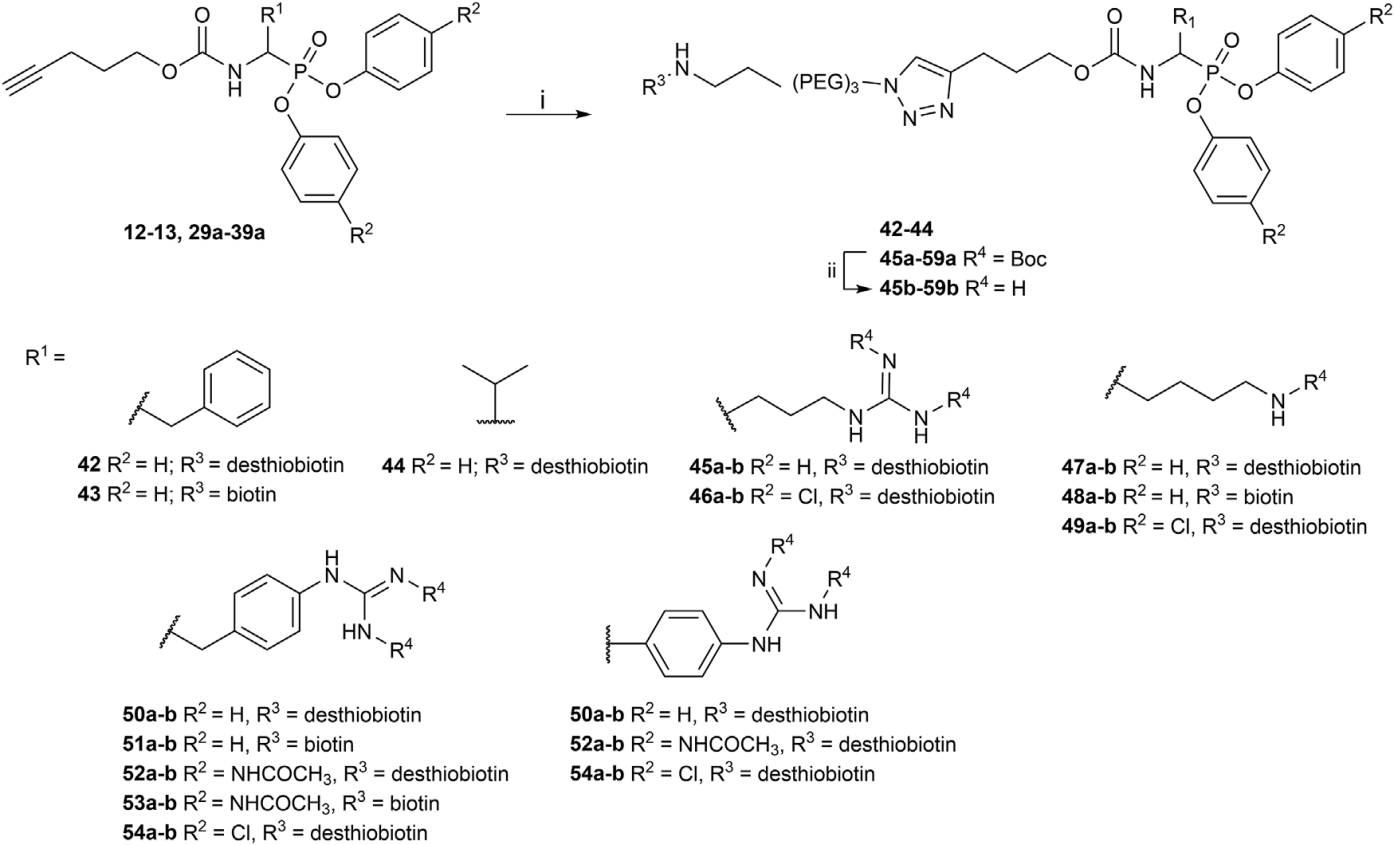
Synthesis of biotin and desthiobiotin probes by click chemistry with reporter tag azide; (i) Biotin/Desthiobiotin-(PEG)_3_-Azide, CuSO_4_·5 H_2_O, Ascorbic acid (Na salt), THF/H_2_O (1:1), rt, 16 h; (ii) TFA, DCM, rt, 2–5 h or HCl (4M, dioxane), rt, 2–5 h.

The obtained alkyne probes can be conjugated to different visualization tags such as rhodamine, biotin, BODIPY, or radiolabeled 4-fluorobenzamide. In this study, we used the affinity tags biotin and desthiobiotin. For the synthesis of the biotin and desthiobiotin probes, the alkyne probe reacted with an azide attached to the reporting tag by click chemistry (Figure 5). This produced full-conversion reactions with easy purifications. When the precursors presented basic nitrogens, these had to be Boc-protected before the cycloaddition to allow easier purifications (29a–39a). In the last step, the clicked probes were deprotected by acid treatment (45b–59b).

### 3.2 Biochemical characterization

The alkyne, biotin, and desthiobiotin probes were tested in a panel of different proteases. The probes were designed to target irreversibly different families of trypsin-like serine proteases. In addition, to test their selectivity, chymotrypsin and elastase-like proteases were also assessed. The panel included four trypsin-like proteases, as well as cathepsin G (CatG), which shares characteristics with chymotrypsin and trypsin-like proteases, and finally, chymotrypsin (ChTryp) and neutrophil elastase (NE).

The IC_50_ values of the alkyne probes were used as a first screening cut-off. All the alkyne probes were initially screened at three concentrations (10, 1, and .1 µM). The probes with an IC_50_ higher than 10 µM were not analyzed further, whereas the rest were submitted to an exact IC_50_ determination. The results of this first screening are presented in Table 2. Based on these results, only the most promising alkyne probes (29–39) were clicked to biotin or desthiobiotin (45–59). These were analyzed in the same manner. Since the alkyne probes share the same warhead and selectivity enhancing group (R^1^) with the biotin and desthiobiotin probes, inhibitory potencies were similar (Table 3), as could be expected. Considering that biotin presents a strong interaction with avidin and may present challenges during the ABP profiling, we have based our study primarily on the desthiobiotin probes. The results of the four biotin probes analyzed are shown in the Supplementary Table S1.

**TABLE 2:** IC_50_ values of diarylphosphonate alkyne probes against a panel of serine proteases.

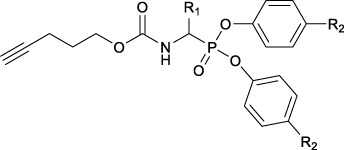									
#	R^1^	R^2^	Trypsin-3	β-tryptase	Thrombin	uPA^a^	CatG^a^	ChTryp^a^	NE^a^
			IC_50_ (μM)^b^						
12	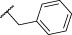	H	>10	>10	>10	>10	>10	1.54 ± 1.34	>10
13	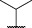	H	>10	>10	>10	>10	>10	>10	>10
14b	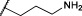	H	>10	>10	>10	>10	>10	>10	>10
16b	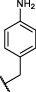	H	6.85 ± 0.80	>10	>10	>10	3.74 ± 0.27	2.67 ± 0.57	>10
17b		NHCOCH_3_	3.71 ± 0.71	>10	2.97 ± 0.07	>10	0.37 ± 0.008	0.49 ± 0.28	>10
18b		Cl	1.72 ± 0.19	>10	7.88 ± 0.61	6.38 ± 0.52	0.36 ± 0.01	0.19 ± 0.14	>10
19b	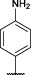	H	9.06 ± 1.10	>10	6.59 ± 3.90	>10	>10	8.52 ± 1.81	>10
20b		NHCOCH_3_	N.D.^c^	>10	N.D.^c^	>10	>10	N.D.^c^	N.D.^c^
21b		Cl	N.D.^c^	>10	N.D.^c^	>10	>10	N.D.^c^	N.D.^c^
22b	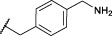	H	3.82 ± 0.19	>10	>10	2.30 ± 0.10	5.26 ± 0.68	>10	>10
23b	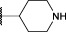	H	>10	>10	>10	>10	>10	>10	>10
29b	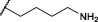	H	0.07 ± 0.02	3.96 ± 0.50	7.36 ± 0.25	5.27 ± 0.15	>10	5.87 ± 3.39	>10
30b		NHCOCH_3_	0.006 ± 0.0006	0.68 ± 0.05	1.18 ± 0.07	2.44 ± 0.35	0.56 ± 0.002	>10	>10
31b	Pro-Lys	H	0.09 ± 0.03	2.98 ± 0.05	0.18 ± 0.003	2.52 ± 0.29	>10	>10	>10
32b	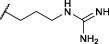	H	0.03 ± 0.007	3.74 ± 0.53	0.85 ± 0.13	4.80 ± 0.08	7.57 ± 0.58	>10	>10
33b		Cl	0.04 ± 0.008	0.17 ± 0.01	0.11 ± 0.007	0.25 ± 0.03	2.76 ± 0.05	>10	N.D.^c^
34b	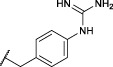	H	0.001 ± 0.0003	0.63 ± 0.03	8.49 ± 2.25	0.008 ± 0.0003	0.45 ± 0.02	>10	>10
35b		NHCOCH_3_	<0.001	0.05 ± 0.003	1.11 ± 0.05	0.005 ± 0.001	0.06 ± 0.006	>10	>10
36b		Cl	<0.001	0.07 ± 0.02	1.77 ± 0.05	0.005 ± 0.0003	0.34 ± 0.008	3.23 ± 1.21	>10
37b	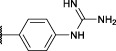	H	0.23 ± 0.01	0.90 ± 0.10	>10	1.79 ± 0.003	0.64 ± 0.03	>10	>10
38b		NHCOCH_3_	0.04 ± 0.015	0.12 ± 0.02	>10	1.52 ± 0.4	0.13 ± 0.01	>10	N.D.^c^
39b		Cl	0.09 ± 0.009	0.07 ± 0.004	>10	0.22 ± 0.06	0.09 ± 0.022	>10	N.D.^c^
40b	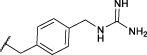	H	>10	>10	>10	>10	6.16 ± 0.48	>10	>10
41b	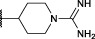	H	0.04 ± 0.002	8.37 ± 0.44	0.03 ± 0.001	>10	>10	>10	>10

^a^ Panel of serine proteases abbreviations: uPA, urokinase plasminogen activator; catG, cathepsin G; ChTryp, chymotrypsin; NE, neutrophil elastase.

^b^ Half maximal inhibitory concentration (IC_50_) value is the concentration of inhibitor required to reduce the enzyme activity to 50% after a 15 min preincubation with the enzyme at 37°C and activity measurements. IC_50_ are calculated from two independent experiments; when SD was higher than three times the average value, a third independent experiment was run (mean ± SD).

^c^ N.D., not determined.

**TABLE 3:** IC_50_ values of diarylphosphonates desthiobiotin probes against a panel of serine proteases.

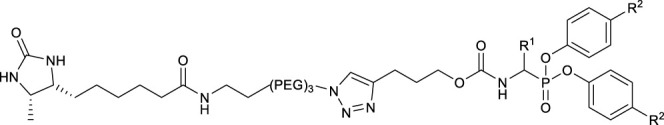									
#	R^1^	R^2^	Trypsin-3	β-tryptase	Thrombin	uPA^a^	CatG^a^	ChTryp^a^	NE^a^
			IC_50_ (μM)^b^						
42	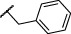	H	>10	>10	>10	>10	3.23 ± 0.29	2.07 ± 0.85	>10
44	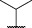	H	>10	>10	>10	>10	>10	>10	4.70 ± 0.20
45b	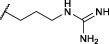	H	0.22 ± 0.02	0.80 ± 0.05	0.61 ± 0.17	>10	>10	>10	N.D.^c^
46b		Cl	0.07 ± 0.002	0.04 ± 0.006	0.04 ± 0.006	0.42 ± 0.05	>10	>10	N.D.^c^
47b	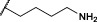	H	0.52 ± 0.24	2.49 ± 0.19	3.55 ± 0.17	4.62 ± 0.17	>10	>10	>10
49b		NHCOCH_3_	0.09 ± 0.005	1.11 ± 0.05	1.18 ± 0.04	6.75 ± 0.52	4.47 ± 0.51	>10	>10
50b	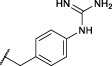	H	0.03 ± 0.01	0.36 ± 0.07	3.52 ± 0.39	0.02 ± 0.0001	0.36 ± 0.004	1.64 ± 0.50	>10
52b		NHCOCH_3_	0.01 ± 0.001	0.07 ± 0.003	0.76 ± 0.02	0.01 ± 0.003	0.05 ± 0.007	>10	>10
54b		Cl	0.008 ± 0.002	0.11 ± 0.009	0.20 ± 0.02	0.006 ± 0.0001	0.12 ± 0.01	5.60 ± 1.06	>10
55b	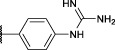	H	0.24 ± 0.01	0.37 ± 0.09	>10	2.22 ± 0.07	1.62 ± 0.09	>10	N.D.^c^
56b		NHCOCH_3_	0.22 ± 0.004	0.39 ± 0.10	>10	5.19 ± 0.08	0.62 ± 0.009	>10	N.D.^c^
57b		Cl	0.18 ± 0.07	0.02 ± 0.0002	>10	0.16 ± 0.04	0.12 ± 0.04	>10	N.D.^c^
58b	Pro-Lys	H	0.16 ± 0.004	6.41 ± 0.15	0.16 ± 0.004	6.51 ± 1.15	2.90 ± 0.15	>10	N.D.^c^

^a^ Panel of serine proteases abbreviations: uPA, urokinase plasminogen activator; catG, cathepsin G; ChTryp, chymotrypsin; NE, neutrophil elastase.

^b^ Half maximal inhibitory concentration (IC_50_) value is the concentration of inhibitor required to reduce the enzyme activity to 50% after a 15 min preincubation with the enzyme at 37°C and activity measurements as mentioned in the Experimental section. IC_50_ are calculated from two independent experiments; when SD was higher than three times the average value, a third independent experiment was run (mean ± SD).

^c^ N.D., not determined.

**FIGURE 6:**
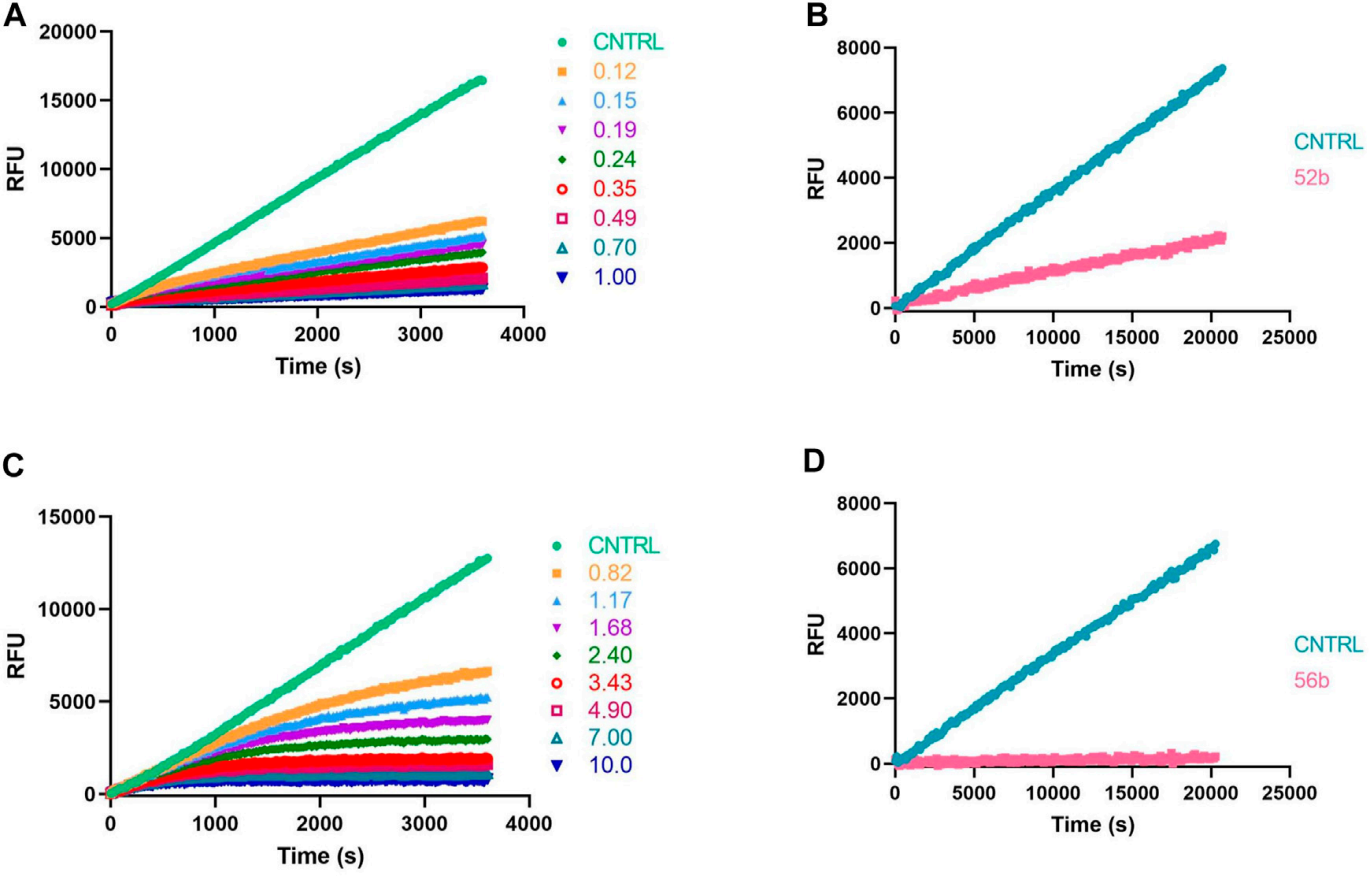
Kinetic progress curves; legend concentrations in µM **(A,C)** and jump dilutions **(B,D)** of β-tryptase; **(A,B)** Reversible compound 52b; **(C,D)** Irreversible compound 56b.

Because ABPs are designed to be irreversible serine protease inhibitors, the IC_50_ is exclusively used as a first discriminatory evaluation. When an inhibitor is irreversible, its IC_50_ value not only depends on the substrate and enzyme concentration but also on the time of incubation with the enzyme before the reaction is started by adding the substrate. Kinetic progress curves can show whether an inhibitor displays time-dependent inhibition. The progress curve in the presence of an irreversible inhibitor will show final velocities equal to zero (Figure 6C). In contrast, for a reversible inhibitor, progress curves have a non-zero final steady-state velocity (Figure 6A) (McWhirter, 2021). To corroborate the inhibitory mechanism of the compounds, jump-dilution assays were performed. These use an inhibitor concentration of 10-fold the IC_50_ for the incubation, and *a posteriori*, the reaction mixture is diluted 10-fold or 100-fold before adding the substrate. In the case of an irreversible inhibitor, enzymatic activity is not regained (Figure 6D), whereas, in the case of a reversible compound, a slow release of the inhibitor and a gradual increase of the enzymatic activity rate is observed (Figure 6B) (Copeland, 2013; McWhirter, 2021). The jump-dilution assays confirmed the behavior described by the progress curves. The results for all probes and proteases are presented in the Supplementary Table S2.

The apparent second-order rate constants (k_app_) were derived from the kinetic progress curves. The results of the kinetic assays resemble those expected for a two-step reversible slow-binding or irreversible mechanism. Thus, the rate constant k_app_ accounts for the initial reversible enzyme-inhibitor complex (E-I) affinity and the formation rate of the reversible high-affinity complex (E*I) or the irreversible enzyme-inhibitor complex (EI). As k_app_ describes the affinity and the reactivity of the inhibitors it is the best metric to discriminate between the different inhibitors. The k_app_ values are shown in Table 4.

**TABLE 4:** k_app_ values of selected alkyne and desthiobiotin probes against a panel of trypsin-like serine proteases and the description of the mechanism of inhibition defined by the progress curves: slow-binding (Rev.) or irreversible (Irrev.).

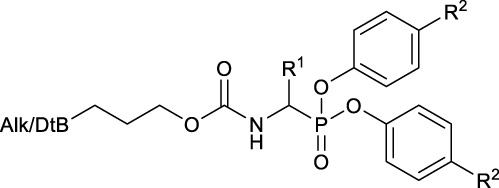								
#	R^1^	R^2^	Alk/DtB^a^	Trypsin-3	β-tryptase	Thrombin	uPA^b^	CatG^b^
				k_app_ (M^−1^ s^−1^)^c^				
32b	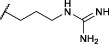	H	Alk	18 × 10^2^ ± 66 Irrev.	41 ± 5 Irrev.	64 ± 8 Rev.	41 ± 4 Irrev.	*^d^ Rev.
45b			DtB	17 × 10^2^ ± 122 Irrev.	237 ± 42 Irrev.	19 ± 2 Rev.	N.D.	N.D.
33b		Cl	Alk	35 × 10^2^ ± 40 Irrev.	28 × 10^2^ ± 82 Irrev.	11 × 10^2^ ± 42 Rev.	15 × 10^2^ ± 12 Irrev.	*^d^ Rev.
46b			DtB	92 × 10^2^ ± 349 Irrev.	75 × 10^2^ ± 1475 Irrev.	63 × 10^2^ ± 290 Rev.	940 ± 37 Irrev.	N.D.
34b	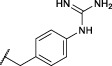	H	Alk	40 × 10^3^ ± 2642 Rev.	712 ± 41 Rev.	17 ± 1 Rev.	75 × 10^2^ ± 725 Irrev.	*^d^ Rev.
50b			DtB	49 × 10^3^ ± 2979 Rev.	32 × 10^2^ ± 613 Rev.	47 ± 5 Rev.	33 × 10^3^ ± 12 × 10^3^ Irrev.	*^d^ Rev.
35b		NHCOCH_3_	Alk	*^d^ Rev.	70 × 10^2^ ± 582 Rev.	128 ± 5 Rev.	13 × 10^4^ ± 30 × 10^3^ Irrev.	477 ± 67 Rev.
52b			DtB	86 × 10^3^ ± 8250 Rev.	18 × 10^3^ ± 1184 Rev.	49 ± 5 Rev.	38 × 10^3^ ± 4175 Irrev.	*^d^ Rev.
36b		Cl	Alk	29 × 10^3^ ± 6499 Rev.	61 × 10^3^ ± 6860 Rev.	251 ± 16 Rev.	32 × 10^4^ ± 29 × 10^3^ Irrev.	*^d^ Rev.
54b			DtB	*^d^ Rev.	*^d^ Rev.	*^d^ Rev.	23 × 10^4^ ± 36 × 10^3^ Irrev.	*^d^ Rev.
37b	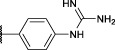	H	Alk	293 ± 34 Irrev.	375 ± 34 Irrev.	N.D.	124 ± 23 Irrev.	582 ± 2 Irrev.
55b			DtB	231 ± 18 Irrev.	347 ± 19 Irrev.	N.D.	77 ± 1 Irrev.	301 ± 85 Irrev.
38b		NHCOCH_3_	Alk	358 ± 6 Irrev.	20 × 10^2^ ± 54 Irrev.	N.D.	215 ± 7 Irrev.	14 × 10^2^ ± 52 Irrev.
56b			DtB	334 ± 85 Irrev.	321 ± 2 Irrev.	N.D.	62 ± 2 Irrev.	420 ± 37 Irrev.
39b		Cl	Alk	11 × 10^2^ ± 164 Irrev.	45 × 10^2^ ± 547 Irrev.	N.D.	25 × 10^2^ ± 86 Irrev.	23 × 10^2^ ± 347 Rev.
57b			DtB	13 × 10^2^ ± 340 Irrev.	65 × 10^2^ ± 1,203 Irrev.	N.D.	25 × 10^2^ ± 11 Irrev.	11 × 10^2^ ± 117 Rev.
41b	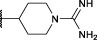	H	Alk	17 × 10^2^ ± 142 Irrev.	27 ± 2 Irrev.	41 × 10^2^ ± 38 Irrev.	N.D.	N.D.
31b	Pro-Lys	H	Alk	13 × 10^2^ ± 28 Irrev.	136 ± 3 Irrev.	13 × 10^2^ ± 5 Irrev.	188 ± 1 Irrev.	N.D.

^a^ Alk, alkyne probe; DtB, desthiobiotin probe.

^b^ Panel of serine proteases abbreviations: uPA, urokinase plasminogen activator; catG, cathepsin G.

^c^ k_app_ are calculated from two independent experiments; when SD was higher than three times the average value, a third independent experiment was run (mean ± SD).

^d^ k_app_ determination is not possible by curve fitting.

N.D.: IC_50_ is greater than 10 μM, and progress curves were not performed.

The equilibrium association constants were derived from the progress curves of trypsin-3, β-tryptase, and thrombin to better describe the affinity of the reversible compounds. The equilibrium constant K1 describes the formation of E-I, whereas the overall process is defined by K_I_*(Copeland, 2013). The equilibrium constants are shown in Table 5.

**TABLE 5:** Apparent equilibrium constants of the first step of slow-binding (K1) and the overall process (K_I_*) for the reversible probes.

#	Trypsin-3		β-tryptase		Thrombin	
	K1 (μM)^a^	K_I_* (μM)^a^	K1 (μM)^a^	K_I_* (μM)^a^	K1 (μM)^a^	K_I_* (μM)^a^
32b	N.D.^b^	N.D.^b^	N.D.^b^	N.D.^b^	22.6 ± 5	0.45 ± 0.03
45b	N.D.^b^	N.D.^b^	N.D.^b^	N.D.^b^	9.97 ± 1.0	1.14 ± 0.11
33b	N.D.^b^	N.D.^b^	N.D.^b^	N.D.^b^	4.13 ± 0.7	0.28 ± 0.04
46b	N.D.^b^	N.D.^b^	N.D.^b^	N.D.^b^	0.95 ± 0.2	0.07 ± 0.009
34b	0.029 ± 0.005	0.003 ± 0.0003	15.8 ± 5	1.11 ± 0.09	134 ± 26	4.93 ± 0.51
50b	0.098 ± 0.022	0.003 ± 0.0002	3.28 ± 0.5	0.41 ± 0.03	*^c^	1.55 ± 0.25
35b	0.001 ± 0.0001	1 × 10^−4^ ± 1 × 10^−5^	3.20 ± 1.8	0.12 ± 0.01	32.7 ± 4	0.63 ± 0.04
52b	0.017 ± 0.0005	0.001 ± 7 × 10^−5^	0.68 ± 0.07	0.06 ± 0.003	*^c^	0.62 ± 0.08
36b	0.008 ± 0.0008	0.001 ± 0.0001	0.34 ± 0.07	0.09 ± 0.01	9.03 ± 0.5	0.03 ± 0.002
54b	0.006 ± 0.0005	8 × 10^−4^ ± 3 × 10^−5^	0.13 ± 0.03	0.02 ± 0.001	*^c^	0.06 ± 0.006

^a^K1 and K_I_* are calculated from two independent experiments; when SD was higher than three times the average value, a third independent experiment was run (mean ± SD).

^b^N.D.: compounds have an irreversible mechanism of inhibition, K1 was not calculated.

^c^ K1 determination is not possible by curve fitting.

Since the alkyne probes and their corresponding biotin/desthiobiotin analogs behave similarly, we will limit the discussion on the relationship between structure and activity to the alkyne probes. The IC_50_ values (Table 2) of the benzyl guanidine probes (34b–36b) show their preference for trypsin-3 and uPA (nanomolar range) and, to a lesser extent, for β-tryptase and CatG, whereas the inhibition of thrombin is negligible. Despite the lower potency against β-tryptase and CatG, compound 35b is the most potent probe of the library for β-tryptase (IC_50_ = .05 ± .003 µM) and CatG (IC_50_ = .06 ± .006 µM). On the other hand, for thrombin, the benzyl guanidine probes were only in the micromolar range.

Despite being the most potent compounds, the progress curves of the benzyl guanidines show an unexpected reversible inhibition of all trypsin-like proteases tested, except for uPA, where the inhibition is irreversible (Table 4). The irreversible inhibition of uPA correlates with previous studies on similar diphenyl phosphonates inhibitors (Joossens et al., 2006). The unexpected reversible inhibition is not related to the chemical properties of the probes, such as low stability or low reactivity, but is dependent on their interaction with the protease (van Soom et al., 2015). Diphenyl phosphonates have been previously described as transition state analogs due to the resemblance with the transition state of peptide hydrolysis. For this type of inhibitor, very high-affinity interactions are expected (Oleksyszyn and Powers, 1991). A reason for not being irreversible might be that the warhead proximity and the geometry toward the serine residue on the active site are not ideal for the nucleophilic attack (Borsari et al., 2022). Alternatively, the compounds could behave as reversible covalent inhibitors where a covalent bond is formed and subsequently hydrolyzed (Tuley and Fast, 2018; McWhirter, 2021).

The k_app_ values of the benzyl guanidines derived from the progress curves correlate with the trends observed for the IC_50_ values. The benzyl guanidine probes show strong inhibition of trypsin-3, β-tryptase, and uPA, weaker for CatG and much weaker for thrombin. For trypsin-3, these probes are at least 10-fold more potent than any of the other probes. The same occurs for β-tryptase, where the k_app_ of the bis(4-chlorophenyl)phosphonate probe 36b (k_app_ = 61 × 10^3^ ± 6860 M^−1^ s^−1^) is 10-fold higher than the second best probes, arginine 33b, and phenyl guanidine 39b. Noteworthy, for uPA, the probe 36b with k_app_ = 32 × 10^4^ ± 29 × 10^3^ M^−1^ s^−1^ is at least 100-fold more potent than the other probes with different side chains. For all reversible benzyl guanidine probes, the values of K_I_* are at least 10-fold lower than the corresponding K1 for trypsin-3 and β-tryptase (Table 5). This is consistent with high-affinity E*I complexes and slow dissociation off-rates. For example, the bis(4-acetamidophenyl)phosphonate alkyne probe (35b) and the bis(4-chlorophenyl)phosphonate desthiobiotin probes (54b) presented a sub-nanomolar affinity for trypsin-3 with K_I_*= 1 × 10^−4^ ± 1 × 10^−5^ and 8 × 10^−4^ ± 3 × 10^−5^ μM, respectively. The rest of the benzyl guanidine probes showed nanomolar affinities for trypsin-3. On β-tryptase, sub-micromolar affinities are achieved with bis(4-chlorophenyl)phosphonate alkyne probe (36b) and both substituted diphenyl phosphonate desthiobiotin probes (52b, 54b).

The phenyl guanidine probes (37b–39b) also demonstrated strong sub-micromolar inhibitory potencies (Table 2). For β-tryptase and CatG, the IC_50_ values are in the same range as for the benzyl guanidine probes (34b–36b). For instance, the IC_50_ for β-tryptase and CatG for the bis(4-chlorophenyl)phosphonate alkyne probe (39b) are .07 ± .004 and .09 ± .022 µM, respectively, which is in the same range as for the best-performing benzyl guanidine probe 35b. For trypsin-3 and uPA, the phenyl guanidine probes lost about 100-fold potency compared to the benzyl guanidine analogs. At the concentrations tested, there was no inhibition of thrombin.

However, in contrast to the benzyl guanidine probes that were only irreversible for uPA, the compounds with phenyl guanidine as a side chain demonstrate irreversible inhibition for all proteases evaluated, excluding the bis(4-chlorophenyl)phosphonate probes (39b, 57b), which, surprisingly, act reversibly on CatG (Table 4). For this series of compounds, the phosphonate aryl substituents (*R* ^2^) have a significant influence on the k_app_, with the bis(4-chlorophenyl)phosphonate probe 39b as the best-performing for all the proteases. This probe has k_app_ values around 20 × 10^2^ M^−1^ s^−1^ for trypsin-3, β-tryptase, uPA, and CatG.

Whereas the benzyl and phenyl guanidine probes did not show potency towards thrombin, the piperidine-1-carboximidamide 41b is the most potent thrombin inhibitor with an IC_50_ = .03 ± .001 µM. It is an equipotent inhibitor for trypsin-3 and has weak inhibition for β-tryptase. There is no inhibition for uPA and CatG. This is also reflected in the k_app_ values obtained from the progress curves with irreversible inhibition of thrombin, trypsin-3, and β-tryptase (Table 4).

Among the probes mimicking the natural amino acids, the arginine side chain proved to be the more interesting for most proteases. In particular, the bis(4-chlorophenyl)phosphonate probes 33b showed high potency and irreversibility for trypsin-3, β-tryptase, and uPA. A similar k_app_ value was obtained for thrombin, although with a reversible inhibition. The lysine-mimicking probes (29b, 30b) had nanomolar inhibition for trypsin-3 but were less potent for β-tryptase, thrombin, uPA, and CatG. The ornithine probe (14b) was inactive in all the proteases tested.

In previous studies, the Pro-Lys mimicking probe was reported to be promising for trypsin-3 with higher inhibitory potency than the Lys probe, but it was less active on β-tryptase (Pan et al., 2006). In our study, the proline-lysine diphenyl phosphonate alkyne (31b) probe was used as a reference compound. This probe showed indeed potent, irreversible inhibition of trypsin-3 and thrombin, whereas the inhibition of β-tryptase and uPA was also irreversible but less potent. However, compared to this reference compound, it is possible to select a more potent probe from our library for each investigated protease.

In general, our results demonstrate that compounds with a terminal guanidine showed significantly higher potencies than their amine analogs. For example, the less basic aniline probes 16b–18b and 19b–21b were inactive or presented IC_50_ higher than 1 µM. The only exceptions are the submicromolar IC_50_ values of 17b and 18b for CatG and chymotrypsin, reflecting the preference of these proteases for aromatic amino acids. In addition, the optimal length of the selectivity enhancing group (R^1^) for this panel of trypsin-like proteases lies between 6 and 8 atoms from the α-carbon. Generally, compounds with shorter or longer R^1^ groups significantly have reduced inhibitory potency. For instance, the benzyl methyl guanidine alkyne probe (40b) only showed affinity for uPA with an IC_50_ = 6.16 ± .48 µM.

The compounds were tested in chymotrypsin and neutrophil elastase to test selectivity over other protease families. All compounds were not potent for neutrophil elastase, including compound 13, designed to target elastase-like proteases. Likewise, most compounds were not potent or only slightly potent for chymotrypsin, with the exception of the above mentioned 17b and 18b.

Concludingly, under these assay conditions, the most potent alkyne probe for trypsin-3 based on the k_app_ values is the benzyl guanidine 34b, with a 5-fold selectivity for uPA, 55-fold over β-tryptase and over 2000-fold for thrombin. Noteworthy, this probe shows a reversible slow-binding inhibitory mechanism. In case that an irreversible probe would be needed, the arginine 32b combines high potency with at least 30-fold selectivity over the other tested proteases. For β-tryptase, several potent reversible probes, such as 36b were obtained, but these probes are not selective for trypsin-3 and uPA. In contrast, probes 33b and 39b are also potent for β-tryptase and show an irreversible mechanism of inhibition. The preferred probe for thrombin is the 4-piperidine-1-carboximidamide (41b), with potent irreversible inhibition and excellent selectivity over β-tryptase, uPA, and CatG. All the probes described in this study are irreversible with uPA. Furthermore, this enzyme has a clear preference for the benzyl guanidine side chain. Last, CatG does not have a clear preference between benzyl guanidine and phenyl guanidine probes. However, benzyl guanidine probes have a reversible mechanism, whereas phenyl guanidine probes are generally irreversible.

### 3.3 Labeling and detection of recombinant proteases

**FIGURE 7:**
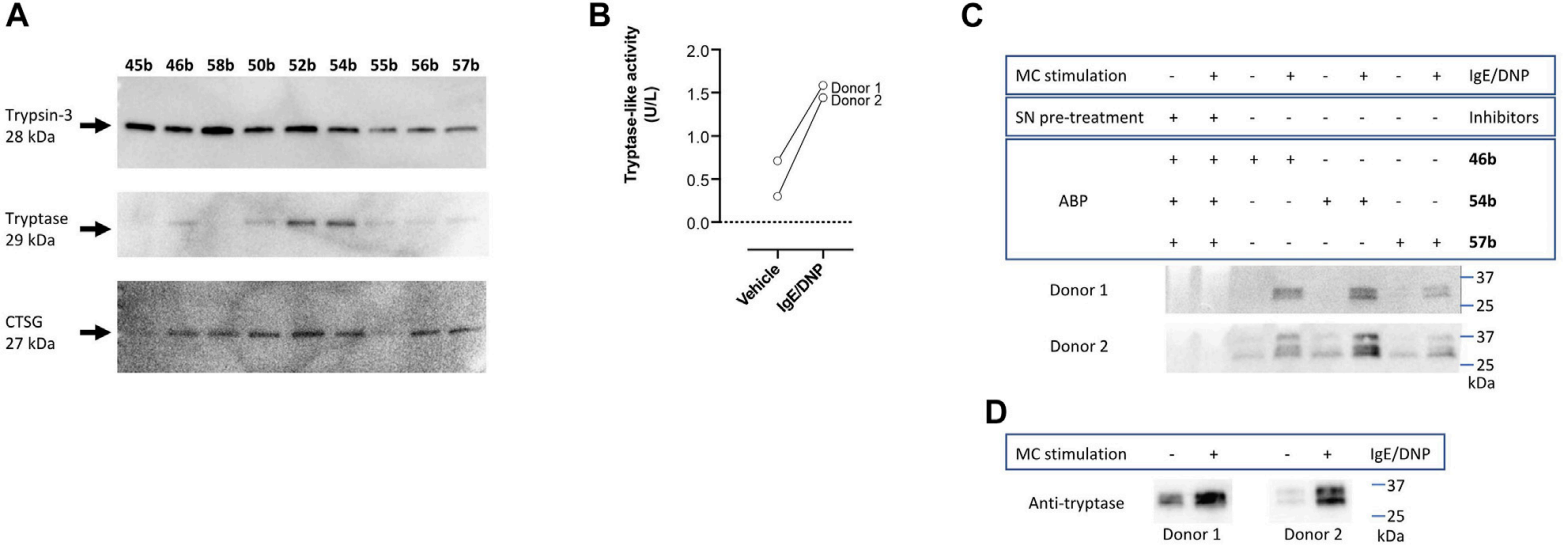
Validation of newly synthesized desthiobiotin probes for detection of trypsin-like proteases. **(A)** Labeling and detection of recombinant proteases in a SDS-PAGE-Blot. **(B)** Tryptase-like activity was measured from the stimulated mast cells of two donors by a spectrophotometric assay with a fluorogenic substrate. IgE/DNP stimulated mast cells were used. The tryptase-like activity is expressed as U/L. **(C)** Labeling of mast cell supernatants and identification of proteases. **(D)** Detection of tryptase in mast cell supernatants with anti-mast cell tryptase antibody, visualized by Western blot.

To further characterize the newly synthesized compounds and their potential to be used as ABPs, nine desthiobiotin probes were used for the labeling and detection of recombinant proteases. The arginine (45b–46b) and the proline-lysine (58b) desthiobiotin probes were used as reference compounds. Whereas the benzyl guanidine (50b, 52b, 54b) and phenyl guanidine probes (55b–57b) were used to determine whether the mechanism of inhibition (reversible or irreversible) is an obstacle to visualize the proteases. Samples of human recombinant proteases (trypsin-3, β-tryptase, and CatG) were incubated with the different probes before electrophoresis and detection with NeutrAvidin-HRP on a Western blot. Figure 7A illustrates the ability of the desthiobiotin probes to indicate the presence of the corresponding trypsin-like enzyme. Surprisingly, the reversible probes with benzyl guanidine as a side-chain (50b, 52b, 54b) can label trypsin-3, β-tryptase, and CatG under denaturing conditions. This effect can be explained due to the strong interactions between the active site of the protease and the probes. The high affinity and the slow off-rates were described in Table 5 by the equilibrium constants K_I_*. Assuming that there is an excess of inhibitor over enzyme, the free inhibitor concentration must be very low, lower than the K_I_*, in order for the complex to dissociate. Therefore, the high affinity complex E*I survives during the whole process of labeling, gel electrophoresis, and visualization.

All the probes tested can label recombinant trypsin-3. However, the phenyl guanidine side chain is the least promising for this enzyme (55b–57b). For β-tryptase, the probes with natural amino acids as a side chain (45b, 46b, 58b) are not good ABP candidates. Both benzyl guanidine and phenyl guanidine show similar labeling intensity. Nevertheless, the probes with bis(4-chlorophenyl)phosphonate (54b, 57b) showed a slightly better resolution for the β-tryptase bands. Last, CatG could be labeled by most of the probes, except for the arginine (45b) and phenylguanidine (55b) unsubstituted diphenyl phosphonate probes.

In summary, the benzyl guanidine probes are compounds with a high affinity for trypsin-like serine proteases. Although they showed a reversible mechanism of inhibition for trypsin-3, β-tryptase, and CatG, surprisingly, these can label the recombinant proteases in a Western blot assay.

### 3.4 Labeling of mast cell supernatants and functional proteomic profiling

The last step to characterize the newly synthesized compounds as potential ABPs was to perform an experiment on biological samples and exemplify the ability of these ABPs to label proteases within a complex proteome. Human mast cells from two donors were stimulated to degranulate with IgE/DNP. Mast cells are immune cells that, upon degranulation, release mediators and play pivotal roles in allergic and inflammatory diseases (Benoist and Mathis, 2002; Theoharides et al., 2012; Aguilera-Lizarraga et al., 2021). Mast cell granules predominantly contain histamine and tryptase. Other serine proteases that are associated with mast cell degranulation, include soluble and transmembrane chymases and CatG (Pejler et al., 2007; Caughey, 2016). First, tryptase-like activity was measured from the stimulated mast cells by a spectrophotometric assay with a fluorogenic substrate (Figure 7B), which was increased compared to the vehicle. Then, mast cell supernatants were labeled with arginine (46b), benzyl guanidine (54b), and phenyl guanidine (57b) bis(4-chlorophenyl)phosphonate desthiobiotin probes. Probes 54b and 57b were chosen due to the good labeling of recombinant tryptase. Again, arginine probe 46b was used as a reference compound. Figure 7C shows that the three probes can label proteases in a complex proteome. The absence of bands when the samples are pre-treated with a cocktail of protease inhibitors indicates that the probes only target active proteases. It is worth noticing that samples from non-stimulated mast cells also can be labeled. Thereby, these probes are also successful at highlighting the constitutive release of proteases, which is relevant in a physiological setting (Caughey, 2016). For both donors, there are two overlapping bands around 30 kDa. The second donor shows an extra band at 37 kDa. After staining with an anti-tryptase antibody, only the lower bands are observed (Figure 7D). These most likely represent α- and β-tryptase (Le et al., 2019). The additional band at 37 kDa might be related to another protease. The benzyl guanidine bis(4-chlorophenyl)phosphonate desthiobiotin (54b) which is the most potent probe for β-tryptase, displayed the best resolution, even though it shows a reversible slow-binding mechanism.

## 4 Discussion

ABPP is a powerful proteomic tool which enables researchers to detect and visualize active enzymes within a complex proteome. ABPP uses chemical probes designed to react covalently with the target enzyme. This tool is attractive in target and biomarker identification and drug discovery programs.

This study focuses on the serine protease family, specifically trypsin-like serine proteases. Most ABPs previously reported targeting this family used a diphenyl phosphonate as a warhead. However, there is a lack of chemical diversity as most of the reported probes contain natural amino acid mimetics as selectivity enhancing moieties. This study aimed to develop an extensive library of ABPs targeting trypsin-like serine proteases with a wide-ranging chemical diversity.

An efficient synthetic route has been implemented for probes bearing a diphenyl phosphonate warhead. A combination of different side chains targeting trypsin-like proteases and the modification of the electrophilicity of the diphenyl phosphonate warhead achieved the desired extensive library of probes. First, the alkyne probes were synthesized, and then the most promising ones were clicked to biotin and desthiobiotin as reporter tags. Furthermore, this synthetic route allows a straightforward approach to couple diverse reporter tags. Thus, alternative tags will enable other applications, such as visualization by fluorescence or PET.

To determine the inhibitory potency of the synthesized probes, their IC_50_ values were determined as a first measurement. However, since the probes were designed to be irreversible inhibitors, the IC_50_ value is not a good parameter to describe their potency. Therefore, kinetic progress curves were measured to determine the mechanism and the kinetic constants of inhibition.

For trypsin-3, β-tryptase, and uPA, the benzyl guanidine probes presented the highest inhibitory activities. Surprisingly, only for uPA, the interaction was irreversible, whereas a reversible slow-binding mechanism with trypsin-3, β-tryptase, thrombin, and CatG was observed. The phenyl guanidines, on the other hand, are good irreversible probes for trypsin-3, β-tryptase, and CatG. The preferred probe for thrombin is the 4-piperidine-1-carboximidamide, with potent irreversible inhibition and excellent selectivity over β-tryptase, uPA, and CatG.

Last, we demonstrated that our probes can label recombinant proteases and tryptase released from mast cell degranulation. Surprisingly, not only the irreversible ABPs but also the probes that presented a reversible slow-binding mechanism can label proteases under denaturing conditions.

Concludingly, these new probes for trypsin-like proteases offer significant advantages in terms of potency over the traditional probes mimicking the natural amino acids arginine and lysine. Detailed enzyme kinetic studies learn that, surprisingly, not all diaryl phosphonates show irreversible inhibition and that the inhibitory mechanism of a specific probe can differ from one protease to another. Even more surprising is the observation that irreversible binding is not needed to maintain protease labeling under denaturing conditions but that also reversible slow-tight binding is sufficient.

## Data Availability

The original contributions presented in the study are included in the article/Supplementary Material, further inquiries can be directed to the corresponding author.
