# Supplementary material for: Chemically diverse activity-based probes with unexpected inhibitory mechanisms targeting trypsin-like serine proteases
Source: Front Chem. 2023 Jan 5;10:1089959. doi: 10.3389/fchem.2022.1089959 (PMC9849758; doi:10.3389/fchem.2022.1089959)
Supplement: Supplementary file 1 [file DataSheet1.PDF]

Supplementary Material

1 Synthetic Schemes

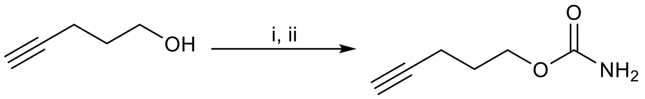

8

Supplementary Figure 1. Synthesis alkyne carbamate 8; (i) CCl_3CONCO, DCM, 0 °C, 1 h; (ii) K_2CO_3, CH_3OH/H_2O (10:1), rt, 16 h.

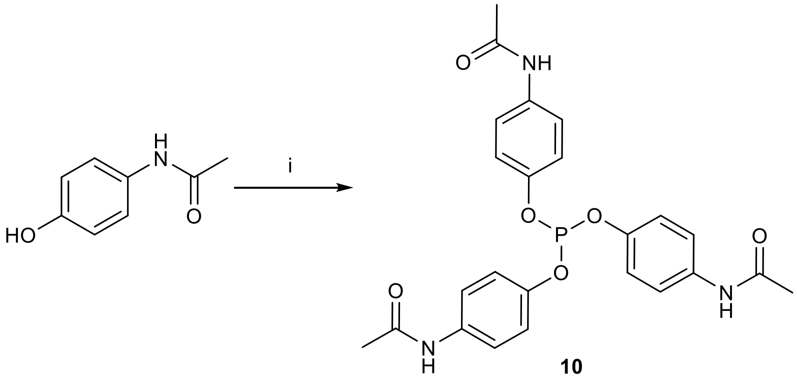

10

Supplementary Figure 2. Synthesis tris(4-acetamidophenyl)phosphite; (i) PCl_3, Et_3N, THF, 0 ^oC to rt, 1 h.

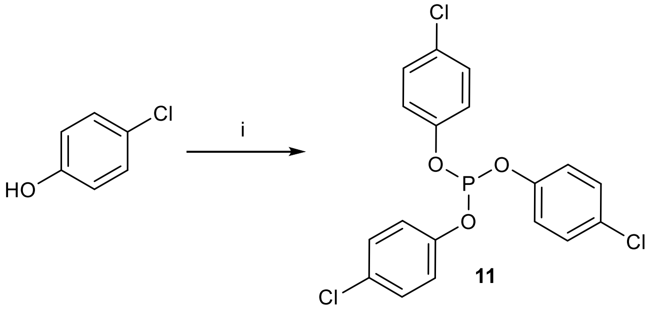

11

Supplementary Figure 3. Synthesis tris(4-chlorophenyl)phosphite; (i) PBr_3, Et_3N, Et_2O, 0 ^oC to rt, 1 h.

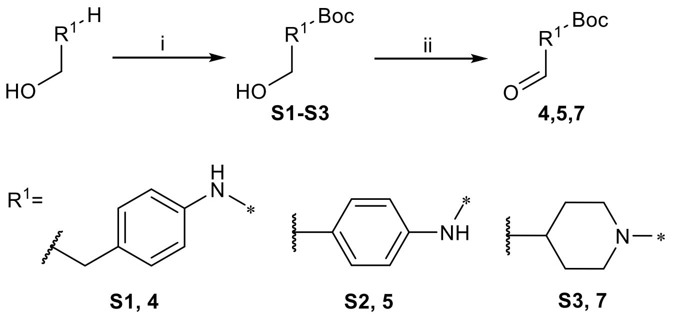

Supplementary Figure 4. Synthesis protected aldehydes (i) Boc_2O, Et_3N, EtOH, rt, 3 h; (ii) DMP, DCM, 0 °C to rt, 4 h.

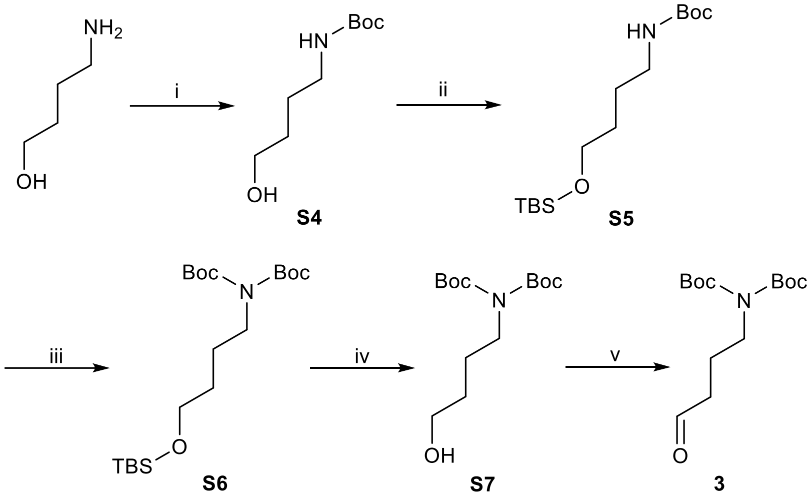

Supplementary Figure 5. Synthesis of protected aldehyde to synthesize arginine analog; (i) Boc_2O, Et_3N, EtOH, rt, 4 h; (ii) Imidazole, TBSCl, DCM, 0 °C, 2 h; (iii) n-BuLi, Boc_2O, THF, 0 °C to rt, 1 h; (iv) TBAF, THF, rt, 5 h; (v) $(COCl)_2$, DMSO, Et_3N, DCM, -78 °C to 0 °C, 2 h.

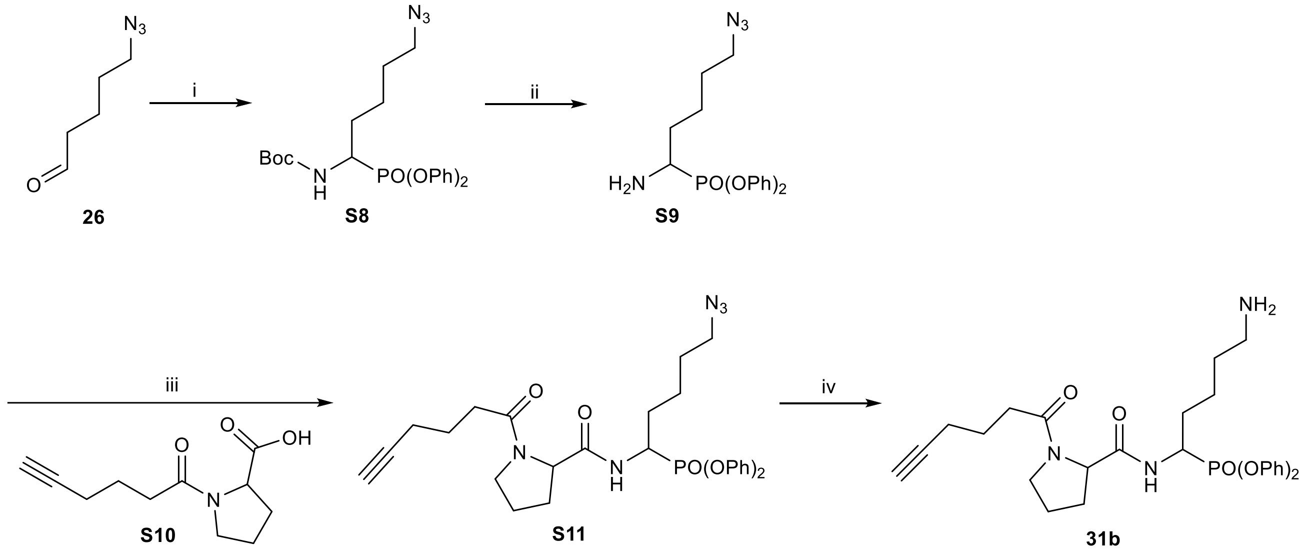

Supplementary Figure 6. Synthesis Pro-Lys ABP precursor; (i) NH_2Boc, $Cu(OTf)_2$, DCM, rt, 16 h; (ii) TFA, DCM, rt, 2 h; (iii) **S10**, HOBt, DIPEA, EDC, DMP, rt, 16 h; (iv) Polymer supported triphenylphosphine, THF, rt, 24 h.

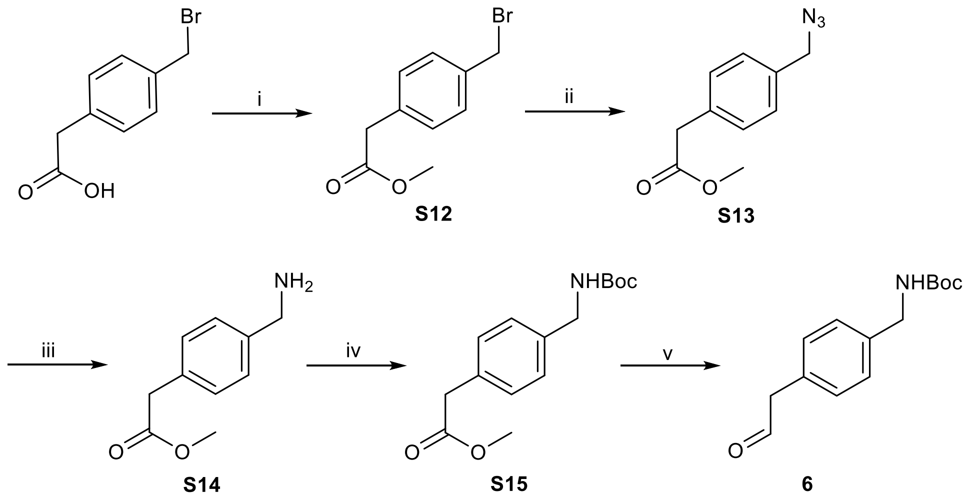

Supplementary Figure 7. Synthesis of protected aldehyde used to synthesize methyl benzyl analogues; (i) $SOCl_2$, CH_3OH, rt, 2 h; (ii) NaN_3, H_2O, 80 °C, 18 h; (iii) Polymer supported triphenylphosphine, THF, rt, 24 h (iv) Boc_2O, Et_3N, EtOH, rt, 16 h; (v) DIBAL-H, toluene, -78°C, 1 h.

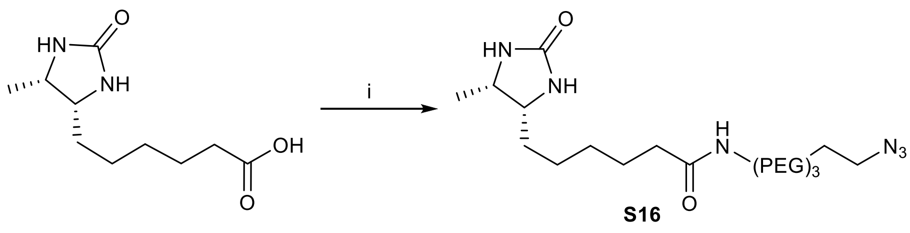

Supplementary Figure 8. Synthesis desthiobiotin azide; (i) 11-Azido-3,6,9-trioxaundecan-1-amine, HOBt, EDC, DIPEA, DMF, rt, 16 h.

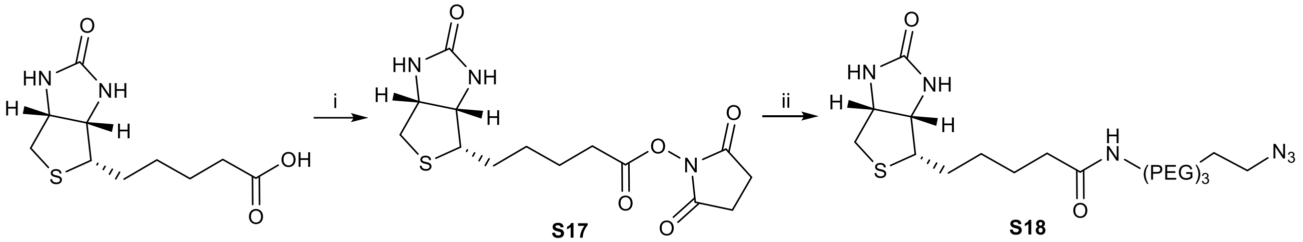

Supplementary Figure 9. Synthesis biotin azide; (i) NHS, DCC, DMF, 70°C, 16 h; (ii) 11-Azido-3,6,9-trioxaundecan-1-amine, Et_3N, DMF, rt, 16 h.

2 Experimental Section

2.1 Chemistry

Every reaction was performed under an N_2 atmosphere if not stated otherwise. Several synthetic procedures used to prepare intermediates and final products are summarised here as “General Procedures”. Target compounds were obtained with a purity >95% and as amorphous solids unless stated otherwise.

2.1.1 General procedures

GENERAL PROCEDURE A: Boc protection; Di-*tert*-butyl dicarbonate (1.1 eq.) was added to a solution of **selected amine** (1 eq.) in EtOH (1M) at room temperature and was stirred for 3 h. The reaction mixture was concentrated under reduced pressure to yield the desired **protected amine**.

GENERAL PROCEDURE B: Dess Martin periodinane oxidation; Dess-Martin periodinane (1.2 eq.) was added portion-wise to a solution of selected **primary alcohol** (1 eq.) in DCM (0.2 M) at 0 °C. The mixture was stirred at room temperature for 4 h. Then, the solvent was removed under reduced pressure. The crude product was purified by column chromatography on silica gel (SiO_2, EtOAc in heptane, 0/100 to 100/0). The desired fractions were collected and concentrated to yield the corresponding **aldehyde**.

GENERAL PROCEDURE C: Birum-Oleksyzyn reaction; Selected **aldehyde** (1 eq.), pent-4-yn-1-yl carbamate (1 eq.), and triphenyl phosphite (if not stated otherwise) (1.1 eq.) were dissolved in anhydrous solvent (0.3 M). Then the Lewis acid (0.1 eq.) was added, and the mixture was stirred at room temperature for 16 h. Then the solvent was evaporated and the residue dissolved in the minimum amount of CH_3OH. The solution was kept at −20 °C for 48 h and then filtrated. When precipitation did not succeed, the crude was purified by flash column chromatography (SiO_2, EtOAc in heptane, 0/100 to 100/0) and, if still not pure, by reverse phase column chromatography (C18, CH_3CN in H_2O, 20/80 to 100/0). The desired fractions were collected and concentrated to yield the corresponding **α-amino diarylphosphonate** as a racemic mixture.

GENERAL PROCEDURE D: Boc deprotection with TFA; TFA (100 eq.) was added to a solution of the selected **protected amine** (1 eq.) in DCM (0.02 M) at room temperature and was stirred for 1-5 h. The volatiles were removed under reduced pressure to yield the corresponding **deprotected amine** as a trifluoroacetate salt.

GENERAL PROCEDURE E: Boc deprotection with HCl; The selected **protected amine** was stirred in HCl (4 M in dioxane) (0.03 M) at room temperature for 1-5 h. The volatiles were removed under reduced pressure to yield the corresponding **deprotected amine** as a hydrochloride salt.

GENERAL PROCEDURE F: Azide deprotection; Polymer supported triphenylphosphine (2 eq.) was suspended in anhydride THF (0.1 M) and stirred for 30 min, then the **azide** (1 eq.) was added. The mixture was stirred at rt for 24 h. Then, water (0.05 M) was added, and the mixture was stirred at rt for 3 h. The polymer was removed by filtration and washed with THF and H_2O. The filtrate was concentrated under reduced pressure and then diluted in EtOH. Di-*tert*-butyl dicarbonate (2 eq.) and triethylamine (2 eq.) were added to the solution. The reaction mixture was stirred at rt for 40 min. The solvent was removed under reduced pressure. The crude product was purified by column chromatography on silica gel (SiO_2, EtOAc in Heptane, 0/100 to 20/80) to yield the corresponding **protected amine**.

GENERAL PROCEDURE G: Guanylation; Triethylamine (6 eq.) was added to a solution of N, N'-bis-Boc-1-guanylpyrazole (2 eq.) and **selected amine** (1 eq.) in DCM/CH_3CN (1:1, 0.1 M) at room temperature. The mixture was stirred for 2-48 h, and the solvent was removed under reduced pressure. EtOAc was added, and the mixture was washed with a solution of HCl (1N), saturated $NaHCO_3$ and brine. The organic layer was dried over Na_2SO_4 and concentrated. The crude product was purified by flash chromatography (SiO_2, EtOAc in heptane, 0/100 to 100/0) to yield the corresponding **protected guanidine**.

GENERAL PROCEDURE H: Click chemistry; L-Ascorbicacidsodium salt (0.6 eq.), BlueCopper (2 eq.) and the selected **alkyl carbamate** (1 eq.) were added to a solution of biotin or desthiobiotin azide (1 eq.) in THF/H_2O (1:1, 0.02 M). The reaction mixture was stirred at room temperature for 16 h. After reaction completion, excess copper was removed by adding Chelex and subsequent filtration. The crude product was purified by reverse phase column chromatography (C18, CH_3CN in H_2O, 20/80 to 100/0). The desired fractions were collected and concentrated to yield the desired **activity-based probe**.

2.1.2 Synthesis and chemical characterization

tert*-butyl (4-hydroxybutyl)carbamate (S4)** General procedure ***A with 4-aminobutan-1-ol (5.17 mL, 56.1 mmol), the residue was purified through a silica plug (50/50 EtOAc in heptane to 100/0 EtOAc). The desired fractions were combined and concentrated *under vacuo* to yield **S4**, which was used without further purification for the next step. ^{1}H NMR (400 MHz, $CDCl_3$) δ (ppm) 4.69 (br s, 1H), 3.64 (d, J = 4.2 Hz, 2H), 3.13 (d, J = 5.6 Hz, 2H), 1.66-1.51 (m, 4H), 1.42 (s, 9H). MS (ESI) *m/z* 212.2 $[M+Na]^+$.

***tert*-butyl (4-((*tert*-butyldimethylsilyl)oxy)butyl)carbamate (S5)** Imidazole (8.62 g, 127 mmol) and *tert*-butyldimethylsilylchlroide (9.16 g, 60.8 mmol) were added to a solution of **S4** in DCM (100 mL) at 0 °C. The reaction mixture was stirred at 0 °C for 2 h. Then Et_2O was added to dilute the reaction mixture and further washed with water and brine. The organic solution was dried over Na_2SO_4, filtered and concentrated to give **S5**, which was used for the next operation directly without further purification. ^{1}H NMR (400 MHz, $CDCl_3$) δ (ppm) 4.69 (s, 1H), 3.68 – 3.58 (m, 2H), 3.14 (d, J = 6.0 Hz, 2H), 1.55 (dt, J = 6.5, 3.5 Hz, 4H), 1.45 (s, 9H), 0.90 (s, 9H), 0.06 (s, 6H). MS (ESI) *m/z* 304.2 $[M+H]^+$.

***tert*-butyl (*tert*-butoxycarbonyl)(4-((*tert*-butyldimethylsilyl)oxy)butyl)carbamate (S6)** *n*-Butyllithium (38.0 mL, 60.8 mmol) was added to a stirred solution of **S5** in THF (250 mL) at 0 ˚C. The

reaction mixture was stirred for 15 min at 0 ˚C before adding di-*t*-butyl dicarbonate (13.27 g, 60.8 mmol). The resulting reaction mixture was warmed to room temperature and stirred for 1 h. Then Et_2O was added to dilute the reaction mixture and washed with water and brine. The organic solution was dried over Na_2SO_4, filtered, and concentrated, giving **S6** used for the next step without further purification. ^{1}H NMR (400 MHz, $CDCl_3$) δ (ppm) 3.62 - 3.49 (m, 4H), 1.64 - 1.52 (m, 2H), 1.51 - 1.41 (m, 20H), 0.85 (s, 9H), 0.00 (s, 6H). No ionization was found.

***N, N'*-bis-*tert*-butyloxycarbonyl 5-aminobutan-1-ol (S7)** Tetrabutylammoniumfluoride (70.0 mL, 70.0 mmol) was added slowly to a stirred solution of **S6** in THF (112 mL) at room temperature. The resulting solution was stirred at room temperature for 5 h. Then Et_2O (100 mL) was added to dilute the reaction mixture and washed with water (100 mL). The aqueous layer was extracted with Et_2O (3 x 80 mL). The combined organic layers were washed with brine, dried over Na_2SO_4, filtered and concentrated under reduced pressure. The crude product was purified by flash chromatography (SiO_2, EtOAc in heptane, 0/100 to 100/0) to yield **S7** (12 g, 41.5 mmol, 74% yield). ^{1}H NMR (400 MHz, $CDCl_3$) δ (ppm) 3.61 (t, J = 6.0 Hz, 2H), 3.56 (t, J = 7.6 Hz, 2H), 1.97 (br s, 1H), 1.66-1.48 (m, 4H), 1.46 (s, 18H). No ionization was found.

***N, N'*-bis-*tert*-butyloxycarbonyl 5-aminobutan-1-al (3)** A solution of DMSO (2.0 mL, 27.6 mmol) in DCM (14 mL) was added over 30 min to a stirred solution of oxalyl chloride (1.2 mL, 13.82 mmol) in DCM (52.5 mL) at -78 °C. Upon completion of the addition, the mixture was stirred at -78 °C for 5 min, followed by adding **S7** (2 g, 6.91 mmol) in DCM (14 mL) over 30 min at -78 °C. The solution was stirred for 40 min after addition completion. Then, triethylamine (5.78 mL, 41.5 mmol) was added dropwise over 10 min. The reaction mixture was let warm to 0 °C and stirred for 1 h. Water was added, followed by ether. The organic layer was washed with water (2x) and brine (2x). The organic layer was dried over Na_2SO_4, filtered and concentrated under reduced pressure to yield **3** (1.98 g, 6.89 mmol, 100% yield) as a yellow oil. ^{1}H NMR (400 MHz, $CDCl_3$) δ (ppm) 9.71 (t, J = 1.3 Hz, 1H), 3.54 (t, J = 7.1 Hz, 2H), 2.40 (td, J = 7.3, 1.3 Hz, 2H), 1.83 (q, J = 7.2 Hz, 2H), 1.43 (s, 18H). No ionization was found.

Tert*-butyl (4-(2-hydroxyethyl)phenyl)carbamate (S1)** General procedure ***A with *p*-aminophenylethanol (4 g, 29.2 mmol) to yield **S1** (6.91 g, 29.1 mmol, 100% yield). ^{1}H NMR (400 MHz, $CDCl_3$) δ (ppm) 7.30 (d, J = 8.3 Hz, 1H), 7.18 – 7.12 (m, 1H), 3.82 (t, J = 6.5 Hz, 1H), 2.82 (t, J = 6.5 Hz, 1H), 1.51 (s, 9H). No ionization was found.

Tert*-butyl (4-(2-oxoethyl)phenyl)carbamate (4)** General procedure ***B with *tert*-butyl (4-(2-hydroxyethyl)phenyl)carbamate (2 g, 8.43 mmol) to yield **4** (1.66 g, 7.06 mmol, 84% yield). ^{1}H NMR (400 MHz, $CDCl_3$) δ 9.71 (t, J = 2.5 Hz, 1H), 7.38 (t, J = 12.5 Hz, 2H), 7.17–7.08 (m, 2H), 3.63 (d, J = 2.5 Hz, 2H), 1.53 (d, J = 7.0 Hz, 9H). No ionization was found.

Tert*-butyl (4-(hydroxymethyl)phenyl)carbamate (S2)** General procedure ***A with *p*-aminobenzylalcohol (1.5 g, 12.18 mmol) to yield **S2** (2.7 g, 12.09 mmol, 99% yield). ^{1}H NMR (400 MHz, $CDCl_3$) δ (ppm) 7.32 (d, J = 8.3 Hz, 2H), 7.27-7.22 (m, 2H), 6.58 (br s, 1H), 4.59 (d, J = 5.6 Hz, 2H), 1.50 (s, 9H). No ionization was found.

Tert*-butyl (4-formylphenyl)carbamate (5)** General procedure ***B with *tert*-butyl (4-(hydroxymethyl)phenyl)carbamate (2.7 g, 12.09 mmol) to yield **5** (1.67 g, 7.55 mmol, 62% yield). ^{1}H NMR (400 MHz, $CDCl_3$) δ (ppm) 9.88 (s, 1H), 7.81 (d, J = 8.5 Hz, 2H), 7.54 (d, J = 8.5 Hz, 2H), 7.00 (br s, 1H), 1.51 (s, 9H). MS (ESI) *m/z* 222.2 $[M+H]^+$.

Methyl 2-(4-(bromomethyl)phenyl)acetate (S12) Sulfurchlorideoxide (0.068 mL, 0.873 mmol) was added to a solution of 2-(4-(bromomethyl)phenyl)acetic acid (2 g, 8.73 mmol) in CH_3OH (30 mL) at room temperature and the mixture was stirred for 1 h. The reaction mixture was concentrated under reduced pressure and neutralized with a saturated solution of $NaHCO_3$. The product was extracted with EtOAc (100 mL). The organic layer was washed with a saturated solution of $NaHCO_3$ (20mL) and brine (20mL), dried, filtered and concentrated under reduced pressure to yield **S12** (2.07 g, 8.52 mmol, 98% yield). ^{1}H NMR (400 MHz, $CDCl_3$) δ (ppm) 7.35 (d, J = 8.0 Hz, 2H), 7.26 (d, J = 8.0 Hz, 2H), 4.48 (s, 2H), 3.69 (s, 3H), 3.62 (s, 2H). MS (ESI) *m/z* 260.0 [M+H_2O].

Methyl 2-(4-(azidomethyl)phenyl)acetate (S13) Sodiumazide (1.498 g, 23.04 mmol) was added to a solution of **S12** (2.8 g, 11.52 mmol) in H_2O (40 mL) at room temperature. Following the addition, the solution was heated to 80 ^oC and stirred for 18 h. The reaction mixture was cooled down to room temperature, and the product was extracted with DCM (3 x 50 mL). The combined organic layers were dried over anhydrous $MgSO_4$, filtered and concentrated under reduced pressure to yield **S13** (2.21 g, 10.77 mmol, 93% yield). ^{1}H NMR (400 MHz, $CDCl_3$) δ (ppm) 7.32-7.24 (m, 4H), 4.31 (s, 2H), 3.69 (s, 3H), 3.63 (s, 2H). MS (ESI) *m/z* 178.1 [M-N_2].

Methyl 2-(4-(aminomethyl)phenyl)acetate (S14) Polymer supported triphenylphosphine (5.63 g, 21.54 mmol) was suspended in anhydride THF (100 mL) and stirred for 30 min, then **S13** (2.21 g, 10.77 mmol) was added. The mixture was stirred at room temperature for 21 h. H_2O (20 mL) was added, and the mixture was stirred for an additional 5 h. The polymer was removed by filtration and washed with THF (2 x 20 mL) and H_2O (20 mL). The filtrate was concentrated under reduced pressure and freeze-dried. The crude product was diluted in DCM (30 mL) and washed with brine (30 mL). The aqueous layer was extracted with DCM (2 x 30 mL), and the combined organic layers were dried over sodium sulfate, filtered and concentrated under reduced pressure to yield **S14** (1.66 g, 9.26 mmol, 86% yield). ^{1}H NMR (400 MHz, $CDCl_3$) δ (ppm) 7.30-7.22 (m, 4H), 3.85 (s, 2H), 3.68 (s, 3H), 3.62 (s, 2H). MS (ESI) *m/z* 357.2 [2xM].

Methyl 2-(4-(((tert-butoxycarbonyl)amino)methyl)phenyl)acetate (S15) General procedure ***A*** with **S14** (1.66 g, 9.26 mmol). The mixture was diluted in EtOAc (100 mL) and washed with a solution of HCl (2N, 100 mL), saturated solution of $NaHCO_3$ (100 mL) and brine. The organic layer was dried over Na_2SO_4, filtered and concentrated under reduced pressure. The crude product was purified by chromatography on silica gel (SiO_2, EtOAc in Heptane, 0/100 to 20/80) to yield **S15** (1.83 g, 6.55 mmol, 71% yield). ^{1}H NMR (400 MHz, $CDCl_3$) δ (ppm) 7.23 (s, 4H), 5.03 (br s, 1H), 4.27 (d, J = 5.9 Hz, 2H), 3.67 (s, 3H), 3.59 (s, 2H), 1.45 (s, 9H). MS (ESI) 302.1 $[M+Na]^+$.

***Tert*-butyl (4-(2-oxoethyl)benzyl)carbamate (6)** A solution of diisopropyl aluminium hydride (10 mL, 1M, 9.29 mmol) was added dropwise to a solution of **S15** (1.73 g, 6.19 mmol) in anhydrous toluene (30 mL) at -78 ^oC. The solution was stirred at -78 ^oC for 1 h and was quenched by adding CH_3OH, followed by adding 10% solution of Rochelle salt. The suspension was diluted in EtOAc, and the layers were separated. The aqueous layer was extracted with EtOAc (2 x 50 mL), and the combined organic layers were dried over $MgSO_4$, filtered and concentrated under reduced pressure to yield **6** (1.30 g, 5.21 mmol, 84% yield). ^{1}H NMR (400 MHz, $CDCl_3$) δ (ppm) 9.73 (t, J = 2.3 Hz, 1H), 7.28 (d, J = 8.0 Hz, 2H), 7.18 (d, J = 8.1 Hz, 2H), 4.93 (br s, 1H), 4.31 (d, J = 6.0 Hz, 2H), 3.68 (d, J = 2.5 Hz, 2H), 1.46 (s, 9H). MS (ESI) *m/z* 523.3 $[2xM+Na]^+$.

Tert*-butyl 4-(hydroxymethyl)piperidine-1-carboxylate (S3)** General procedure ***A with piperidin-4-ylmethanol (2 g, 17.36 mmol) to yield **S3** (3.7 g, 17.19 mmol, 99% yield). ^{1}H-NMR (400 MHz, $CDCl_3$) δ (ppm) 4.08 (br s, 2H), 3.45 (t, J = 5.80, 2H), 2.76-2.58 (m, 2H), 2.12 (br s, 1H), 1.68 (d, 2H, J =

14.04), 1.64-1.55 (m, 1H), 1.4 (s, 9H), 1.10 (ddd, *J* = 4.1, 12.3, 24.4 Hz, 2H). MS (ESI) *m/z* 238.2 $[M+Na]^+$.

Tert*-butyl 4-formylpiperidine-1-carboxylate (7)** General procedure ***B with **S3** (1.0 g, 4.64 mmol) to yield ***7*** (0.99 g, 4.64 mmol, 99% yield). ^{1}H-NMR (400 MHz, $CDCl_3$) δ (ppm) 9.65 (s, 1H), 3.97 (br s, 2H), 2.97-2.87 (m, 2H), 2.45-2.35 (m, 1H), 1.89 (d, *J* = 13.0 Hz, 2H), 1.61-1.52 (m, 2H), 1.45 (s, 9H). No ionization was found.

Pent-4-yn-1-yl carbamate (8) Trichloroacetylisocyanate (5 mL, 42.0 mmol) was added to a solution of 4-pentyn-1-ol (3.23 mL, 35.0 mmol) in dry DCM (70 mL) at 0 °C. The reaction mixture was allowed to warm up to room temperature and was stirred for 1h. The solvent was removed *under vacuo,* and the mixture was dissolved in CH_3OH (70 mL) and water (10 mL). Potassium carbonate (7.25 g, 52.5 mmol) was added portion-wise, and the mixture was stirred overnight at room temperature. The solvent was removed *under vacuo,* and water (100 mL) was added. The aqueous layer was extracted with EtOAc (3 x 100mL). The combined organic layers were washed with brine solution, dried over $MgSO_4$, and removed the solvent *under vacuo*. The crude product was recrystallized from DCM/heptane to yield **8** (4.289 g, 33.7 mmol, 96.0% yield). ^{1}H NMR (400 MHz, $CDCl_3$) δ (ppm) 4.76 (s, 2H), 4.16 (t, *J* = 6.3 Hz, 2H), 2.29 (td, *J* = 7.1, 2.7 Hz, 1H), 1.97 (t, *J* = 2.7 Hz, 1H), 1.89 – 1.80 (m, 2H). MS (ESI) *m/z* 150 $[M+Na]^+$.

Tris (4-acetamidophenyl) phosphite (10) Triethylamine (0.95 mL, 6.86 mmol) was added dropwise to a stirred solution of phosphorus trichloride (0.2 mL, 2.287 mmol) and acetaminophen (1.037 g, 6.86 mmol) in THF (20 mL) at 0 °C. Upon addition completion, the reaction mixture was stirred at 0 °C for 15 min and then was slowly left to reach room temperature and stirred for 2 h. The mixture was filtered through a celite pad, and the filtrate was concentrated under reduced pressure. The crude product **10** was obtained as a white foam (1.1 g, 2.28 mmol, 100%) and used for the next synthetic step without further purification. MS (ESI) *m/z* 482.2 $[M+H]^+$.

Tris(4-chlorophenyl) phosphite (11) Phosphorustribromide (0.6 mL, 6.32 mmol) was added to a solution of 4-chlorophenol (2.436 g, 18.95 mmol) and triethylamine (3.52 mL, 25.3 mmol) in dry diethyl ether (20 mL) at 0 ^{o}C. Following the addition, the mixture was warmed to room temperature and stirred for 1h. The mixture was filtered through a celite pad. The filtrate was washed with a solution of HCl (1N, 20 mL), a saturated solution of $NaHCO_3$ (20 mL) and brine. The organic layer was concentrated under reduced pressure. The crude product was purified by flash column chromatography (SiO_2, Heptane 100%) to yield **11** (1.76 g, 4.26 mmol, 67% yield). ^{1}H NMR (400 MHz, $CDCl_3$) δ 7.04 (dd, *J* = 8.9, 1.1 Hz, 6H), 7.29 (dt, *J* = 8.9, 2.0 Hz, 6H). No ionization was found.

Pent-4-yn-1-yl (1-(diphenoxyphosphoryl)-2-phenylethyl)carbamate (12) General procedure ***C*** with phenylethanal (0.483 mL, 4.16 mmol) and copper (II) trifluoromethanesulfonate in DCM to yield **12** (1.21 g, 2.59 mmol, 62% yield). ^{1}H NMR (400 MHz, $CDCl_3$) δ (ppm) 7.38-7.09 (m, 15H), 5.08 (d, *J* = 10.5 Hz, 1H), 4.86-4.72 (m, 1H), 4.13-3.99 (m, 2H), 3.48-3.36 (m, 1H), 3.13-2.97 (m, 1H), 2.16 (td, *J* = 10.5, 2.3 Hz, 2H), 1.96 (t, *J* = 2.6 Hz, 1H), 1.75-1.67 (m, 2H). ^{13}C NMR (100 MHz, $CDCl_3$) δ (ppm) 155.7, 155.6, 150.3, 150.2, 150.0, 149.9, 135.8, 135.7, 129.8, 129.7, 129.3, 128.5, 127.0, 125.5, 125.3, 120.6, 120.5, 120.4, 120.3, 83.0, 68.9, 63.9, 50.0, 48.4, 36.0, 35.9, 27.7, 15.0. MS (ESI) *m/z* 464.2 $[M+H]^+$. HRMS for $C_{26}H_{26}N_1O_5P_1$, mass calculated 464.1621, mass measured 464.1634.

Pent-4-yn-1-yl (1-(diphenoxyphosphoryl)-2-methylpropyl)carbamate (13) General procedure ***C*** with isobutyraldehyde (0.25 mL, 2.77 mmol) and copper (II) trifluoromethanesulfonate in DCM to

yield **13** (0.63 g, 1.52 mmol, 55% yield). ^{1}H NMR (400 MHz, $CDCl_3$) *δ* (ppm) 7.39-7.27 (m, 4H), 7.25-7.10 (m, 6H), 5.24 (d, *J* = 10.4 Hz, 1H), 4.44 (ddd, *J* = 19.6, 12.9, 4.0 Hz, 1H), 4.21 (td, *J* = 6.4, 3.2 Hz, 2H), 2.50-2.37 (m, 1H), 2.27 (td, *J* = 7.0, 2.8 Hz, 2H), 2.00 (t, *J* = 2.8 Hz, 1H), 1.84 (p, *J* = 6.6 Hz, 2H), 1.16-1.08 (m, 6H). ^{13}C NMR (100 MHz, $CDCl_3$) *δ* (ppm) 156.3, 150.3, 150.0, 129.8, 125.4, 120.6, 83.0, 69.2, 64.4, 53.3, 29.2, 27.8, 20.4, 17.7, 15.1. MS (ESI) m/z 416.2 $[M+H]^+$. HRMS for $C_{22}H_{26}N_1O_5P_1$, mass calculated 416.1621, mass measured 416.1611.

Tert*-butyl (*tert*-butoxycarbonyl)(4-(diphenoxyphosphoryl)-4-(((pent-4-yn-1-yloxy)carbonyl)amino) butyl) carbamate (14a)** General procedure ***C with **3** (2.2 g, 7.66 mmol) and copper (II) trifluoromethanesulfonate in DCM to yield **14a** (215 mg, 0.34 mmol, 5% yield). ^{1}H NMR (400 MHz, $CDCl_3$) *δ* (ppm) 7.31 (q, *J* = 7.9 Hz, 4H), 7.20-7.13 (m, 4H), 7.12 (d, *J* = 8.5 Hz, 2H), 5.15 (d, *J* = 10.3 Hz, 1H), 4.56-4.43 (m, 1H), 4.23-4.10 (m, 2H), 3.63 (t, *J* = 6.6 Hz, 2H), 2.25 (td, *J* = 7.0, 2.6 Hz, 2H), 1.97 (t, *J* = 2.6 Hz, 1H), 1.88-1.77 (m, 4H), 1.48 (s, 18H). MS (ESI) *m/z* 653.3 $[M+Na]^+$.

Pent-4-yn-1-yl (4-amino-1-(diphenoxyphosphoryl)butyl)carbamate trifluoroacetate salt (14b) General procedure ***D*** with **14a** (70 mg, 0.13 mmol) to yield **14b** (71 mg, 0.13 mmol, 99% yield). ^{1}H NMR (400 MHz, Methanol-*d*$_4$) *δ* (ppm) 7.89 (d, *J* = 9.84 Hz, 1H), 7.39 (t, *J* = 7.9 Hz, 4H), 7.28-7.22 (m, 2H), 7.19 (d, *J* = 7.8 Hz, 4H), 4.51-4.39 (m, 1H), 4.25-4.12 (m, 2H), 3.07-2.97 (m, 2H), 2.32-2.26 (m, 3H), 2.21-2.10 (m, 1H), 2.01-1.89 (m, 2H), 1.89-1.77 (m, 3H). ^{13}C NMR (100 MHz, Methanol-*d*$_4$) *δ* (ppm) 157.3, 157.2, 150.2, 150.1, 150.0, 149.9, 129.6, 129.6, 125.5, 125.4, 120.4, 120.3, 120.2, 120.1, 82.4, 68.9, 63.8, 38.6, 27.7, 26.0, 25.9, 23.8, 23.7, 14.3. MS (ESI) *m/z* 431.3 $[M+H]^+$. HRMS for $C_{22}H_{27}N_2O_5P_1$, mass calculated 431.1730, mass measured 431.1747.

Tert*-butyl (4-(bis(4-chlorophenoxy)phosphoryl)-4-(((pent-4-yn-1-yloxy)carbonyl)amino)butyl) (*tert*-butoxycarbonyl)carbamate (15a)** General procedure ***C with **3** (1 g, 3.48 mmol), **11**, and bismuth(III) trifluoromethanesulfonate in THF to yield **15a** (210 mg, 0.35 mmol, 10% yield). ^{1}H NMR (400 MHz, $CDCl_3$) *δ* (ppm) 7.31-7.26 (m, 4H), 7.10 (dd, *J* = 12.8, 8.5 Hz, 4H), 5.10 (d, *J* = 10.0 Hz, 1H), 4.59 (br s, 1H), 4.52 – 4.38 (m, 1H), 4.19 (t, *J* = 6.2 Hz, 2H), 3.18 (br d, *J* = 5.7 Hz, 2H), 2.26 (td, *J* = 7.0, 2.5 Hz, 2H), 2.10 (m, 1H), 1.98 (t, *J* = 2.6 Hz, 1H), 1.87 – 1.61 (m, 5H), 1.43 (s, 9H). MS (ESI) *m/z* 621.1 $[M+Na]^+$.

Pent-4-yn-1-yl (4-amino-1-(bis(4-chlorophenoxy)phosphoryl)butyl)carbamate trifluoroacetate salt (15b) General procedure ***D*** with **15a** (150 mg, 0.25 mmol) to yield **15b** (123 mg, 0.246 mmol, 98% yield). ^{1}H NMR (400 MHz, Methanol-*d*$_4$) *δ* (ppm) 7.88 (d, *J* = 9.8 Hz, 1H), 7.41 (d, *J* = 8.8 Hz, 4H), 7.24 – 7.16 (m, 4H), 4.52 – 4.40 (m, 1H), 4.26 – 4.10 (m, 2H), 3.08 – 2.94 (m, 2H), 2.33 – 2.25 (m, 3H), 2.22 – 2.05 (m, 1H), 2.02 – 1.75 (m, 5H). MS (ESI) *m/z* 499.1 $[M+H]^+$.

Pent-4-yn-1-yl (2-(4-((tert-butoxycarbonyl)amino)phenyl)-1-(diphenoxyphosphoryl)ethyl)carbamate (16a) General procedure ***C*** with **4** (1.40 g, 5.99 mmol) and copper (II) trifluoromethanesulfonate in DCM to yield **16a** (1.0 g, 1.72 mmol, 29% yield). ^{1}H NMR (400 MHz, DMSO-*d*$_6$) *δ* 9.28 (s, 1H), 8.00 (d, *J* = 9.5 Hz, 1H), 7.45 – 7.32 (m, 6H), 7.27 – 7.14 (m, 8H), 4.40 (dd, *J* = 26.5, 9.5 Hz, 1H), 3.90 (dtd, *J* = 17.0, 10.8, 6.2 Hz, 2H), 3.17 (d, *J* = 14.6 Hz, 1H), 2.96 – 2.84 (m, 1H), 2.77 (t, *J* = 2.5 Hz, 1H), 2.13 (td, *J* = 7.0, 2.5 Hz, 2H), 1.66 – 1.54 (m, 2H), 1.46 (s, 9H). MS (ESI) *m/z* 601.3 $[M+Na]^+$.

Pent-4-yn-1-yl (2-(4-aminophenyl)-1-(diphenoxyphosphoryl)ethyl)carbamate hydrochloride salt (16b) General procedure ***E*** with **16a** (120 mg, 0.207 mmol) to yield **16b** (98 mg, 0.207 mmol, 99% yield). ^{1}H NMR (400 MHz, Methanol-*d*$_4$) *δ* (ppm) 7.89 (d, *J* = 9.9 Hz, 1H), 7.50 (d, *J* = 8.2 Hz, 2H), 7.38 (q, *J* = 8.1 Hz, 6H), 7.26 (d, *J* = 7.2 Hz, 2H), 7.24-7.17 (m, 4H), 4.73-4.61 (m, 1H), 4.08-3.94 (m,

2H), 3.46 (dt, J = 14.0, 4.3 Hz, 1H). 3.17-3.05 (m, 1H), 2.26 (t, J = 2.6 Hz, 1H), 2.19 (td, J = 7.1, 2.6 Hz, 2H), 1.71 (p, J = 6.7 Hz, 2H). ^{13}C NMR (100 MHz, Methanol-d_4) δ (ppm) 156.9, 150.3, 150.2, 150.0, 149.9, 137.8, 137.6, 130.8, 129.6, 129.6, 125.5, 125.4, 122.5, 120.4, 120.3, 120.2, 120.1, 82.4, 68.8, 63.5, 34.4, 34.3, 27.7, 14.2. MS (ESI) *m/z* 479.3 $[M+H]^+$. HRMS for $C_{26}H_{27}N_2O_5P_1$, mass calculated 479.1730, mass measured 479.1727.

Pent-4-yn-1-yl (1-(bis(4-acetamidophenoxy)phosphoryl)-2-(4-((tert-butoxycarbonyl)amino) phenyl)ethyl) carbamate (17a) General procedure ***C*** with **4** (0.75 g, 3.19 mmol), **10** and boron trifluoride etherate in CH_3CN to yield **17a** (420 mg, 0.60 mmol, 19% yield). ^{1}HNMR (400 MHz, $CDCl_3$) δ (ppm) 8.35 (s, 2H), 7.33 (t, J= 7.54 Hz, 4H), 7.29-7.26 (m, 2H), 7.11 (d, J=8.08, 2H), 6.96 (d, J=8.25 Hz, 2H), 6.91 (d, J=8.35 Hz, 2H), 6.76 (br s, 1H), 4.75-4.61 (m, 1H), 4.14-3.97 (m, 2H), 3.35-3.22 (m, 1H), 3.05-2.92 (m, 1H), 2.12 (t, J=6.54 Hz, 2H), 2.04 (s, 6H), 1.97-1.91 (m, 1H), 1.72-1.62 (m, 2H), 1.50 (s, 9H). MS (ESI) *m/z* 710.0 $[M+NH_4]^+$.

Pent-4-yn-1-yl (2-(4-aminophenyl)-1-(bis(4-acetamidophenoxy)phosphoryl)ethyl)carbamate trifluoroacetate salt (17b) General procedure ***D*** with **17a** (120 mg, 0.173 mmol) to yield **17b** (100 mg, 0.169 mmol, 97% yield). ^{1}H NMR (400 MHz, Methanol-d_4) δ (ppm) 7.54 (d, J = 8.8 Hz, 4H) 7.38 (d, J = 8.4 Hz, 2H), 7.18 (d, J = 8.4 Hz, 2H), 7.17-7.11 (m, 4H), 4.71-4.59 (m, 1H), 4.08-3.96 (m, 2H), 3.49-3.41 (m, 1H), 3.15-3.04 (m, 1H), 2.25 (t, J = 2.4 Hz, 1H), 2.22-2.17 (m, 2H), 2.11 (s, 6H), 1.74-1.66 (m, 2H). ^{13}C NMR (100 MHz, Methanol-d_4) δ (ppm) 170.2, 130.8, 122.6, 121.0, 120.9, 120.6, 120.5, 120.3, 120.2, 82.4, 68.8, 63.5, 27.7, 22.3, 14.2. MS (ESI) *m/z* 593.2 $[M+H]^+$. HRMS for $C_{30}H_{33}N_4O_7P_1$, mass calculated 593.2160, mass measured 593.2155.

Pent-4-yn-1-yl (1-(bis(4-chlorophenoxy)phosphoryl)-2-(4-((tert-butoxycarbonyl)amino)phenyl)ethyl) carbamate (18a) General procedure ***C*** with **4** (0.88g, 3.74 mmol), **11** and boron trifluoride etherate in CH_3CN to yield **18a** (234 mg, 0.361 mmol, 10%). ^{1}H NMR (400 MHz, $CDCl_3$) δ 7.34-7.2234 (m, 6H), 7.14 (d, J = 8.6 Hz, 2H), 7.09 (dd, J = 8.8, 1.2 Hz, 2H), 7.02 (dd, J = 8.7, 1.2 Hz, 2H), 6.68 (br s, 1H), 4.93 (d, J = 10.3 Hz, 1H,), 4.77-4.59 (m, 1H), 4.10-3.98 (m, 2H), 3.32 (dd, J = 14.7, 12.9, 4.6 Hz, 1H), 2.97 (dt, J = 14.3, 10.3 Hz, 1H), 2.14 (td, J = 6.9, 2.6 Hz, 2H), 1.96 (t, J = 2.7 Hz, 1H), 1.73 (p, J = 6.7 Hz, 2H), 1.68-1.54 (m, 2H), 1.49 (s, 9H). MS (ESI) *m/z* 669.2 $[M+Na]^+$.

Pent-4-yn-1-yl 2-(4-aminophenyl)-1-(bis(4-chlorophenoxy)phosphoryl)ethyl carbamate hydrochloride salt (18b) General procedure ***E*** with **18a** (230 mg, 0.355 mmol) to yield **18b** (200 mg, 0.343 mmol, 96% yield). ^{1}H NMR (400 MHz, Methanol-d_4) δ (ppm) 7.99 (br d, J = 9.3 Hz, 1H), 7.50 (d, J = 8.0 Hz, 2H), 7.43-7.34 (m, 6H), 7.20 (td, J = 8.3, 1.2 Hz, 4H), 4.75-4.59 (m, 1H), 4.07-3.93 (m, 2H), 3.44 (dt, J = 14.0, 4.1 Hz, 1H), 3.10 (ddd, J = 25.1, 14.4, 9.9 Hz, 1H), 2.26 (t, J = 2.4 Hz, 1H), 2.17 (td, J = 7.4, 2.6 Hz, 2H), 1.69 (p, J = 6.6 Hz, 2H). ^{13}C NMR (100 MHz, Methanol-d_4) δ (ppm) 158.3, 150.3, 150.2, 139.4, 132.3, 131.0, 130.9, 124.2, 123.4, 123.1, 83.8, 70.3, 64.9, 51.0, 35.7, 29.1, 15.6. MS (ESI) *m/z* 547.1 $[M+H]^+$.

Pent-4-yn-1-yl ((4-((tert-butoxycarbonyl)amino)phenyl)(diphenoxyphosphoryl)methyl)carbamate (19a) General procedure ***C*** with **5** (1.67 g, 7.55 mmol) and copper(II) trifluoromethanesulfonate in DCM to yield **19a** (2.12 g, 3.76 mmol, 50% yield). ^{1}H NMR (400 MHz, $CDCl_3$) δ 7.48-7.01 (m, 12H), 6.89 (d, J = 8.3 Hz, 2H), 6.55 (s, 1H), 5.75 (dd, J = 9.9, 5.7 Hz, 1H), 5.50 (dd, J = 22.4, 10.2 Hz, 1H), 4.26-4.06 (m, 2H), 2.23 (t, J = 6.5 Hz, 2H), 1.95 (t, J = 2.5 Hz, 1H), 1.80 (p, J = 6.5 Hz, 2H), 1.51 (s, 9H). MS (ESI) *m/z* 587.2 $[M+Na]^+$.

Pent-4-yn-1-yl ((4-aminophenyl)(diphenoxyphosphoryl)methyl)carbamate trifluoroacetate salt (19b) General procedure ***D*** with **19a** (57 mg, 0.101 mmol) to yield **19b** (57 mg, 0.099 mmol, 98% yield). ^{1}H NMR (400 MHz, Methanol-d_4) δ (ppm) 8.66 (br d, J = 10.4 Hz, 1H), 7.70 (d, J = 7.7 Hz, 2H), 7.41-7.25 (m, 6H), 7.24-7.14 (m, 2H), 7.08 (d, J = 7.3 Hz, 2H), 7.00 (d, J = 8.1 Hz, 2H), 5.70 (br d, J = 23.2 Hz, 1H), 4.27-4.08 (m, 2H), 2.33-2.20 (m, 3H), 1.83 (br t, J = 6.1 Hz, 2H). ^{13}C NMR (100 MHz, Methanol-d_4) δ (ppm) 158.3, 151.6, 151.3, 135.4, 134.5, 131.3, 131.0, 126.9, 123.7, 121.5, 83.9, 70.3, 65.4, 53.8, 29.2, 15.7. MS (ESI) *m/z* 465.2 [M+H]$^+$. HRMS for $C_{25}H_{25}N_2O_5P_1$, mass calculated 465.1574, mass measured 465.1583.

Pent-4-yn-1-yl ((bis(4-acetamidophenoxy)phosphoryl)(4-((tert-butoxycarbonyl)amino) phenyl)methyl) carbamate (20a) General procedure ***C*** with **5** (1.68 g, 7.59 mmol), **10**, and copper (II) trifluoromethanesulfonate in THF to yield **20a** (1.73 g, 2.56 mmol, 34% yield). ^{1}H NMR (400 MHz, DMSO-d_6) δ (ppm) 9.98 (s, 1H), 9.43 (s, 1H), 8.66 (d, J = 9.51 Hz, 1H), 7.53-7.42 (m, J = 8.8 Hz, 8H), 6.99 (d, J = 8.8 Hz, 2H), 6.90 (d, J = 6.8 Hz, 2H), 5.40 (dd, J = 22, 10.3 Hz, 1H), 4.12-3.96 (m, 2H), 2.80 (s, 1H), 2.23 (td, J = 7.2, 2.6 Hz, 2H), 2.01 (s, 6H), 1.72 (p, J = 6.7 Hz, 2H), 1.47 (s, 9H). MS (ESI) *m/z* 701.4 [M+Na]$^+$.

Pent-4-yn-1-yl ((4-aminophenyl)(bis(4-acetamidophenoxy)phosphoryl)methyl)carbamate trifluoroacetate salt (20b) General procedure ***D*** with **20a** (0.8 g, 1.179 mmol) to yield **20b** (0.68 g, 1.173 mmol, 99% yield). ^{1}H NMR (400 MHz, Methanol-d_4) δ (ppm) 7.73 (dd, J = 8.7, 2.3 Hz, 1H), 7.50 (t, J = 9.0 Hz, 2H), 7.42 (d, J = 8.6 Hz, 1H), 7.02 (d, J = 7.7 Hz, 1H), 6.95 (d, J = 7.6 Hz, 1H), 5.69 (d, J = 8.3 Hz, 1H), 4.26 – 4.08 (m, 1H), 2.29 (d, J = 9.8 Hz, 1H), 2.10 (s, 3H), 1.82 (p, J = 6.4 Hz, 1H). ^{13}C NMR (100 MHz, Methanol-d_4) δ (ppm) 170.2, 145.8, 145.7, 136.2, 134.9, 131.9, 130.0, 129.9, 122.7, 121.0, 120.9, 120.3, 120.3, 120.2, 82.3, 68.8, 64.0, 53.0, 51.4, 39.0, 27.7, 22.3, 14.3. MS (ESI) *m/z* 601.2 [M+Na]$^+$. HRMS for $C_{29}H_{31}N_4O_7P_1$, mass calculated 579.2003, mass measured 579.2013.

Pent-4-yn-1-yl ((bis(4-chlorophenoxy)phosphoryl)(4-((tert-butoxycarbonyl)amino)phenyl) methyl)carbamate (21a) General procedure ***C*** with **5** (1.69 g, 7.64 mmol), **11**, and copper (II) trifluoromethanesulfonate in THF to yield **21a** (2 g, 3.16 mmol, 41% yield). ^{1}H NMR (400 MHz, CDCl$_3$) δ (ppm) 7.48–7.32 (m, 4H), 7.29 (d, J = 8.2 Hz, 2H), 7.18 (d, J = 9.0 Hz, 2H), 7.08 (d, J = 9.0 Hz, 2H), 6.81 (d, J = 8.4 Hz, 2H), 6.61 (s, 1H), 5.52 (dd, J = 21.9, 10.2 Hz, 1H), 4.22-4.10 (m, 2H), 2.27-2.18 (m, 2H), 1.95 (t, J = 2.7 Hz, 1H), 1.79 (p, J = 6.7 Hz, 2H), 1.54 (s, 9H). MS (ESI) *m/z* 655.2 [M+Na]$^+$.

Pent-4-yn-1-yl ((4-aminophenyl)(bis(4-chlorophenoxy)phosphoryl)methyl)carbamate trifluoroacetate salt (21b) General procedure ***D*** with **21a** (0.8 g, 1.263 mmol) to yield **21b** (0.65 g, 1.216 mmol, 96% yield). ^{1}H NMR (400 MHz, Methanol-d_4) δ 7.72 (dd, J = 8.7, 2.3 Hz, 2H), 7.40 (d, J = 8.4 Hz, 2H), 7.34 (t, J = 9.5 Hz, 4H), 7.10 (d, J = 8.8 Hz, 2H), 7.01 (d, J = 10.4 Hz, 2H), 5.73 (d, J = 21.8 Hz, 1H), 4.26 – 4.09 (m, 2H), 2.34 – 2.17 (m, 3H), 1.87 – 1.76 (m, 2H). ^{13}C NMR (100 MHz, Methanol-d_4) δ (ppm) 156.7, 148.6, 148.5, 133.6, 133.1, 130.9, 130.8, 129.9, 129.8, 129.6, 129.5, 122.2, 121.7, 121.6, 121.6, 121.6, 82.4, 68.9, 64.0, 53.0, 51.4, 29.5, 29.4, 27.7, 22.3, 14.3. MS (ESI) *m/z* 555.1 [M+Na]$^+$. HRMS for $C_{25}H_{23}N_2O_5Cl_2P_1$, mass calculated 533.0794, mass measured 533.0775.

Pent-4-yn-1-yl (2-(4-(((*tert*-butoxycarbonyl)amino)methyl)phenyl)-1 (diphenoxyphosphoryl)ethyl) carbamate (22a) General procedure ***C*** with **6** (1.30 g, 5.21 mmol) and copper(II) trifluoromethanesulfonate in DCM to yield **22a** (1.4 g, 2.362 mmol, 45% yield). ^{1}H NMR (400 MHz, CDCl$_3$) δ (ppm) 7.37-7.27 (m, 4H), 7.24-7.08 (m, 10H), 5.23-5.06 (m, 1H), 4.96-4.67 (m,

2H), 4.29 (d, *J* = 5.6 Hz, 2H), 4.14-3.98 (m, 2H), 3.45-3.31 (m, 1H), 3.02 (dt, *J* = 14.5, 10.0 Hz, 1H), 2.13 (t, *J* = 6.7 Hz, 2H), 1.97 (t, *J* = 2.7 Hz, 1H), 1.70 (p, *J* = 6.7 Hz, 2H), 1.46 (s, 9H). MS (ESI) *m/z* 615.2 $[M+Na]^+$.

Pent-4-yn-1-yl (2-(4-(aminomethyl)phenyl)-1-(diphenoxyphosphoryl)ethyl)carbamate hydrochloride salt (22b) General procedure ***E*** with **22a** (42 mg, 0.071 mmol) to yield **22b** (37 mg, 0.070 mmol, 99% yield). ^{1}H NMR (400 MHz, Methanol-*d₄*) δ (ppm) 7.95 (d, *J* = 10.1 Hz, 1H), 7.49-7.32 (m, 8H), 7.28-7.14 (m, 6H), 4.71-4.57 (m, 1H), 4.10 (s, 2H), 4.06-3.91 (m, 2H), 3.78-3.60 (m, 1H), 3.42 (dt, *J* = 14.0, 4.3 Hz, 1H), 3.07 (ddd, *J* = 25.6, 13.3, 8.4 Hz, 1H), 2.26 (t, *J* = 2.2 Hz, 1H), 2.16 (td, *J* = 7.3, 2.4 Hz, 2H), 1.69 (p, *J* = 6.7 Hz, 2H). ^{13}C NMR (100 MHz, Methanol-d_4) δ (ppm) 158.3, 151.7, 151.6, 151.4, 151.3, 139.4, 139.3, 133.1, 131.1, 131.0, 130.2, 126.8, 126.7, 121.8, 121.5, 83.9, 70.2, 64.8, 51.3, 44.0, 35.9, 29.2, 15.6. MS (ESI) *m/z* 493.2 $[M+H]^+$. HRMS for $C_{27}H_{29}N_2O_5P_1$, mass calculated 493.1887, mass measured 493.1881.

Tert*-butyl 4-((diphenoxyphosphoryl)(((pent-4-yn-1-yloxy)carbonyl)amino)methyl)piperidine-1-carboxylate (23a)** General procedure ***C with **7** (0.99 g, 4.69 mmol) and copper(II) trifluoromethanesulfonate in DCM to yield **23a** (1.2 g, 2.15 mmol, 46% yield). ^{1}H-NMR (400 MHz, $CDCl_3$) δ (ppm) 7.35 – 7.27 (m, 4H), 7.21 – 7.14 (m, 4H), 7.14 – 7.10 (m, 2H), 5.24-5.15 (m, 1H), 4.52-4.39 (m, 1H), 4.26-4.09 (m, 4H), 2.79-2.61 (br s, 2H), 2.30-2.12 (m, 3H), 2.01-1.91 (m, 2H), 1.87-1.73 (m, 3H), 1.44 (s, 9H), 1.40-1.32 (m, 1H). MS (ESI) *m/z* 579.2 $[M+Na]^+$.

Pent-4-yn-1-yl ((diphenoxyphosphoryl)(piperidin-4-yl)methyl)carbamate trifluoroacetate salt (23b) General procedure ***D*** with **23a** (32 mg, 0.057 mmol) to yield **23b** (26 mg, 0.057 mmol, 99%). ^{1}H NMR (400 MHz, Methanol-d_4) δ (ppm) 7.40 (t, *J* = 7.8 Hz, 4H), 7.28-7.22 (m, 2H), 7.22-7.16 (m, 4H), 4.47 (dd, *J* = 19.2, 6.3 Hz, 1H), 4.25 - 4.13 (m, 2H), 3.47 (t, *J* = 11.3 Hz, 2H), 3.13 - 3.01 (m, 2H), 2.46 - 2.34 (m, 1H), 2.32 – 2.26 (m, 3H), 2.26 – 2.17 (m, 2H), 1.88 – 1.79 (m, 2H), 1.79 – 1.66 (m, 2H). ^{13}C NMR (100 MHz, Methanol-d_4) δ (ppm) 157.4, 157.3, 150.1, 150.0, 149.9, 149.8, 129.7, 129.6, 125.5, 125.4, 120.4, 120.4, 120.2, 120.1, 82.3, 68.9, 63.9, 52.7, 51.1, 43.4, 43.3, 34.8, 34.7, 27.8, 26.3, 26.2, 24.8, 24.7, 14.3. MS (ESI) *m/z* 457.3 $[M+H]^+$. HRMS for $C_{24}H_{29}N_2O_5P_1$, mass calculated 457.1887, mass measured 457.1903.

5-azidopentan-1-ol (25) Sodiumazide (1.074 g, 16.52 mmol) was added to a solution of pentamethylenebromohydrin (1 mL, 8.26 mmol) in H_2O (15 mL). The reaction mixture was stirred at 80 °C for 18 h. After cooling down to room temperature, the aqueous solution was extracted with DCM (3 x 20 mL). The combined organic layers were dried over anhydrous $MgSO_4$, filtered and concentrated under reduced pressure to yield **25** (1.00 g, 7.80 mmol, 94% yield) as a colourless oil used for the next step without any further purification. ^{1}H NMR (400 MHz, $CDCl_3$) δ (ppm) 1.47-1.36 (m, 2H), 1.65-1.50 (m, 4H), 2.06 (br t, *J* = 4.2 Hz, 1H), 3.26 (t, *J* = 6.7 Hz, 2H), 3.63 (dd, *J* = 6.2, 4.0 Hz, 2H). No ionization was found.

5-azidopentanal (26) General procedure ***B*** with **25** (1 g, 7.74 mmol) to yield **26** (0.48 g, 3.81 mmol, 49% yield). ^{1}H NMR (400 MHz, $CDCl_3$) δ (ppm) 1.64-1.55 (m, 2H), 1.75-1.64 (m, 2H), 2.47 (td, *J* = 6.8, 1.4 Hz, 2H), 3.28 (t, *J* = 7.0 Hz, 2H), 9.74 (t, *J* = 1.5 Hz, 1H). No ionization was found.

Pent-4-yn-1-yl (5-azido-1-(diphenoxyphosphoryl)pentyl)carbamate (27) General procedure ***C*** with **26** (0.99 g, 7.81 mmol) and copper (II) trifluoromethanesulfonate in DCM to yield **27** (2.62 g, 5.57 mmol, 71% yield). ^{1}H NMR (400 MHz, $CDCl_3$) δ (ppm) 7.38-7.27 (m, 4H), 7.22-7.07 (m, 6H), 5.22 (br d, *J* = 10.4 Hz, 1H), 4.74 (br s, 1H), 4.53-4.39 (m, 1H), 4.25-4.11m, 2H), 3.28 (t, *J* = 6.4 Hz, 2H),

2.34-2.18 (m, 2H), 2.13-1.99 (m, 1H), 1.97 (q, *J* = 2.6 Hz, 1H), 1.89-1.43 (m, 6H). MS (ESI) *m/z* 471.3 $[M+H]^+$.

Pent-4-yn-1-yl (5-azido-1-(bis(4-acetamidophenoxy)phosphoryl)pentyl)carbamate (28) General procedure ***C*** with **26** (0.481 g, 3.78 mmol), tris(4-acetamidophenyl) phosphite and Boron trifluoride etherate in CH_3CN to yield **28** (1.3 g, 2.22 mmol, 59% yield). ^1H NMR (400 MHz, $CDCl_3$) δ (ppm) 8.78 (d, *J* = 9.7 Hz, 2H), 7.30 (dd, *J* = 8.7, 5.5 Hz, 4H), 6.89 (t, *J* = 8.3 Hz, 4H) 5.98 (d, *J* = 10.1 Hz, 1H), 4.51-4.35 (m, 1H), 4.23-4.14 (m, 2H), 3.27 (t, *J* = 6.0 Hz, 2H), 2.22 (td, *J* = 6.7, 2.7 Hz, 2H), 2.05-1.94 (m, 9H), 1.79 (p, *J* = 6.5 Hz, 2H), 1.72-1.44 (m, 4H). MS (ESI) *m/z* 585.3 $[M+H]^+$.

Tert*-butyl pent-4-yn-1-yl (1-(diphenoxyphosphoryl)pentane-1,5-diyl)dicarbamate (29a)** General procedure ***F with **27** (0.765 g, 1.627 mmol) to yield **29a** (0.49 g, 0.91 mmol, 64% yield). ^1H NMR (400 MHz, Methanol-d_4) δ (ppm) 7.36 (t, *J* = 7.7 Hz, 4H), 7.22 (td, *J* = 7.4, 3.1 Hz, 2H), 7.19-7.13 (m, 4H), 4.38 (ddd, *J* = 15.7, 13.4, 3.5 Hz, 1H), 4.12 (m, 2H), 4.09 (t, *J* = 6.3 Hz, 2H), 3.06 (t, *J* = 6.0 Hz, 2H), 2.32-2.23 (m, 4H), 2.10-1.97 (m, 1H), 1.92-1.72 (m, 4H), 1.52-1.45 (m, 2H), 1.43 (s, 9H). MS (ESI) *m/z* 445.3 $[(M-Boc)+H]^+$.

Pent-4-yn-1-yl (5-amino-1-(diphenoxyphosphoryl)pentyl)carbamate trifluoroacetate salt (29b) General procedure ***D*** with **29a** (57 mg, 0.105 mmol) to yield **29b** (52 mg, 0.093 mmol, 89% yield). ^1H NMR (400 MHz, Methanol-d_4) δ (ppm) 7.81 (d, *J* = 9.8 Hz, 1H), 7.37 (t, *J* = 7.5 Hz, 4H), 7.22 (t, *J* = 7.4 Hz, 2H), 7.16 (d, *J* = 8.6 Hz, 4H), 4.48-4.34 (m, 1H), 4.24-4.08 (m, 2H), 2.93 (t, *J* = 6.9 Hz, 2H), 2.31-2.23 (m, 3H), 2.14-1.99 (m, 1H), 1.98-1.45 (m, 7H).^{13}C NMR (100 MHz, Methanol-d_4) δ (ppm) 158.7, 151.6, 151.4, 131.0, 126.7, 121.8, 121.6, 83.9, 70.3, 65.1, 40.5, 29.8, 29.2, 27.9, 23.9, 23.7, 15.7. MS (ESI) *m/z* 445.3 $[M+H]^+$. HRMS for $C_{23}H_{29}N_2O_5P_1$, mass calculated 445.1887, mass measured 445.1899.

Tert*-butyl pent-4-yn-1-yl (1-(bis(4-acetamidophenoxy)phosphoryl)pentane-1,5-diyl)dicarbamate (30a)** General procedure ***F with **28** (0.20 g, 0.342 mmol) to yield **30a** (0.078 g, 0.119 mmol, 35% yield). ^1H NMR (400 MHz, $CDCl_3$) δ (ppm) 8.73 (d, *J* = 14.8 Hz, 2H), 7.93 (d, *J* = 8.1 Hz, 4H), 6.90 (dd, *J* 13.7, 8.7 Hz, 4H), 5.99 (d, *J* = 9.5 Hz, 1H), 4.83 (t, *J* = 5.8 Hz, 1H), 4.48-4.32 (m, 1H), 4.17 (t, *J* = 5.9 Hz, 2H), 3.13-2.97 (m, 2H), 2.27-2.12 (m, 3H), 2.03 (s, 9H), 1.97 (t, *J* = 2.8 Hz, 2H), 1.79 (p, *J* = 6.7 Hz, 2H), 1.36-1.60 (m, 13H). MS (ESI) *m/z* 559.2 $[(M-Boc)+H]^+$.

Pent-4-yn-1-yl (5-amino-1-(bis(4-acetamidophenoxy)phosphoryl)pentyl)carbamate hydrochloride salt (30b) General procedure ***E*** with **30a** (42 mg, 0.064 mmol) to yield **30b** (25.3 mg, 0.043 mmol, 67% yield). ^1H NMR (400 MHz, Methanol-d_4) δ (ppm) 7.55 (d, *J* = 8.5 Hz, 4H), 7.11 (d, *J* = 8.7 Hz, 4H), 4.38 (ddd, *J* = 16.8, 13.4, 3.6 Hz, 1H), 4.24-4.08 (m, 2H), 3.77-3.70 (m, 1H), 3.69-3.67 (m, 2H), 3.60-3.55 (m, 1H), 2.93 (t, *J* = 7.2 Hz, 2H), 2.31-2.24 (m, 3H), 2.11 (s, 6H), 2.06-1.45 (m, 8H). ^{13}C NMR (100 MHz, Methanol-d_4) δ (ppm): 171.6, 158.7, 137.7, 122.4, 122.0, 121.7, 83.9, 73.6, 72.5, 70.3, 68.2, 65.2, 62.2, 40.5, 29.8, 29.2, 27.8, 23.8, 15.7. MS (ESI) *m/z* 559.2 $[M+H]^+$.

***Tert*-butyl (5-azido-1-(diphenoxyphosphoryl)pentyl)carbamate (S8)** Copper (II) trifluoromethanesulfonate (0.65 g, 1.81 mmol) was added to a solution of **26** (2.3 g, 18.09 mmol), *tert*-butyl carbamate (2.12 g, 18.09 mmol) and **9** (5.2 mL, 19.90 mmol) in DCM (40 mL) at room temperature and the mixture was stirred for 16 h. The solvent was removed under reduced pressure. The crude product was purified by flash chromatography (SiO_2, EtOAc in Heptane, 0/100 to 20/80) to yield **S8** (5.7 g, 12.46 mmol, 69% yield). ^1H NMR (400 MHz, Methanol-d_4) δ (ppm) 7.38-7.29 (m, 4H) 7.25-7.10 (m, 6H), 4.36 (ddd, *J* = 15.5, 13.8, 3.9 Hz, 1H), 3.33-3.26 (m, 2H), 2.07-1.95 (m, 1H), 1.91-1.76 (m, 1H), 1.73-1.46 (m, 4H), 1.44 (s, 9H). MS (ESI) *m/z* 483.2 $[M+Na]^+$.

Diphenyl (1-amino-5-azidopentyl)phosphonate hydrochloric salt (S9) General procedure ***E*** with **S8** (3.65 g, 7.93 mmol) to yield **S9** (3.15 g, 7.94 mmol, 99% yield). ^{1}H NMR (400 MHz, $CDCl_3$) δ (ppm) 10.05 (br s, 2H), 7.34-7.26 (m, 4H), 7.20-7.16 (m, 2H), 7.12-6.99 (m, 4H), 4.06-3.90 (m, 1H), 3.37-3.16 (m, 2H), 2.37-1.95 (m, 2H), 1.82-1.47 (m, 4H). MS (ESI) *m/z* 361.8 $[M+H]^+$.

Hex-5-ynoylproline (S10) DIPEA (12.6 mL, 72.5 mmol) was added to a solution of H-Pro-OtBu·HCl (8.28 g, 39.9 mmol), hex-5-ynoic acid (4 mL, 36.2 mmol) EDC (8.34 g, 43.5 mmol) and HOBt (6.66 g, 43.5 mmol) in DMF (50 mL). The reaction mixture is stirred for 2 h at room temperature. Upon completion, it is diluted with EtOAc (100 mL) and washed with 1M HCl (50 mL). The aqueous layer is extracted with EtOAc (3x50 mL) and the combined organic layers were washed with saturated $NaHCO_3$ (60 mL) and brine (60 mL). The organic layer is dried over anhydrous magnesium sulfate, filtered and the solvent evaporated under *vacuo*. The crude was purified by flash chromatography (SiO_2, EtOAc in heptane, 0/100 to 100/0) to yield *tert*-butyl hex-5-ynoylprolinate (6.97 g, 26.3 mmol, 72% yield). ^{1}H NMR (400 MHz, $CDCl_3$) δ (ppm) 4.34 (dd, *J* = 8.6, 3.4 Hz, 2H), 3.65-3.42 (m, 2H), 2.42 (td, *J* = 7.39, 2.4 Hz, 2H), 2.28-2.21 (m, 2H), 1.97-1.80 (m, 6H), 1.42 (s, 9H). MS (ESI) *m/z* 288.2 $[M+Na]^+$. General procedure ***D*** with *tert*-butyl hex 5-ynoylprolinate (6.97 g, 26.3 mmol) to yield **S10** (5.5 g, 26.3 mmol, 99% yield). ^{1}H NMR (400 MHz, $CDCl_3$) δ (ppm) 1.89-1.78 (m, 2H), 2.34-1.93 (m, 7H), 2.53 (t, *J* = 6.9 Hz, 2H), 3.63-3.51 (m, 1H), 3.77-3.63 (m, 1H,), 4.52 (dd, *J* = 7.9, 4.7 Hz, 1H). MS (ESI) *m/z* 210.2 $[M+H]^+$.

Diphenyl (5-azido-1-(1-(hex-5-ynoyl)pyrrolidine-2-carboxamido)pentyl)phosphonate (S11) N,N-Di-iso-propylethylamine (5.5 mL, 31.8 mmol) was added to a solution of **S9** (3.15 g, 7.94 mmol), **S10** (2.66 g, 12.70 mmol), 1-hydroxybenzotriazolehydrate (1.46 g, 9.53 mmol), EDC (1.83 g, 9.53 mmol) in DMF (50 mL). The mixture was stirred at room temperature for 16 h. The solvent was coevaporated with toluene and the resulting mixture was diluted in EtOAc (100 mL), washed with a solution of HCl (1N, 50 mL), saturated aqueous solution of $NaHCO_3$ (60 mL) and brine. The organic layer was dried over anhydrous magnesium sulfate, filtered, and concentrated under reduced pressure. The crude product was purified by column chromatography on silica gel (SiO_2, EtOAc in Heptane, 0/100 to 70/30) to yield **S11** (2.15 g, 3.90 mmol, 49% yield). ^{1}H NMR (400 MHz, $CDCl_3$) δ (ppm): 7.81 (br d, *J* = 10.2 Hz, 1H), 7.53 (br d, *J* = 10.2 Hz, 1H), 7.27-7.35 (m, 4H), 7.20-7.26 (m, 2H), 7.12-7.20 (m, 4H), 7.07-7.12 (m, 1H), 4.66-4.83 (m, 2H), 4.50 (dd, *J* = 8.3, 1.5 Hz, 1H), 3.55-3.65 (m, 1H), 3.34-3.51 (m, 2H), 3.26 (dd, *J* = 12.8, 6.3 Hz, 2H), 2.48 (td, *J* = 7.4, 1.7 Hz, 2H), 2.32-2.42 (m, 1H), 2.29 (td, *J* = 7.2, 2.6 Hz, 2H), 2.20 (td, *J* = 6.8, 2.6 Hz, 1H), 1.34-2.17 (m, 9H). MS *m/z* 552.3 $[M+H]^+$.

Tert*-butyl (5-(diphenoxyphosphoryl)-5-(1-(hex-5-ynoyl)pyrrolidine-2-carboxamido)pentyl) carbamate (31a)** General procedure ***F with **S11** (1.0 g, 1.810 mmol) to yield **31a** (112 mg, 0.179 mmol, 10% yield). ^{1}H NMR (400 MHz, $CDCl_3$) δ (ppm): 7.70 (br d, *J* = 10.1 Hz, 1H), 7.38-7.25 (m, 4H), 7.25-7.18 (m, 2H), 7.18-7.11 (m, 4H), 7.09 (d, *J* = 8.2 Hz, 1H), 5.05 (br t, *J* = 5.4 Hz, 1H), 4.82-4.65 (m, 2H), 4.44 (d, *J* = 8.0 Hz, 1H), 3.67-3.57 (m, 1H), 3.50-3.31 (m, 2H), 3.14-2.98 (m, 2H), 2.48 (td, *J* = 7.2, 3.3 Hz, 1H), 2.44-2.30 (m, 1H), 2.27 (td, *J* = 6.8, 2.6 Hz, 1H), 2.24-1.45 (m, 13H), 1.41 (s, 9H). MS *m/z* 648.3 $[M+Na]^+$.

(5-(diphenoxyphosphoryl)-5-(1-(hex-5-ynoyl)pyrrolidine-2-carboxamido)pentyl) carbamate trifluoroacetate salt (31b) General procedure ***D*** with **31a** (42.6 mg, 0.07 mmol) to yield **31b** (41 mg, 0.06 mmol, 94% yield). ^{1}H NMR (400 MHz, Methanol-d_4) δ (ppm) 7.44-7.30 (m, 4H), 7.30-7.05 (m, 6H), 4.79-4.68 (m, 2H), 4.47 (dd, *J* = 8.7, 5.3 Hz, 1H), 3.79-3.54 (m, 4H), 3.32 (t, *J* = 1.7 Hz, 1H), 3.06-2.87 (m, 2H), 2.53 (t, *J* = 7.3 Hz, 1H), 2.34-1.38 (m, 15H). ^{13}C NMR (100 MHz, Methanol-d_4) δ (ppm) 175.1, 173.8, 151.6, 151.4, 131.0, 126.9, 121.9, 121.7, 121.6, 84.3, 70.4, 61.2, 47.5, 45.9, 40.7,

34.1, 30.9, 29.2, 27.4, 25.9, 25.8, 25.0, 23.5, 18.6. MS *m/z* 526.3 $[M+H]^+$. HRMS for $C_{29}H_{36}N_3O_5P_1$, mass calculated 526.2465, mass measured 526.2457.

N, N'-bis-tert-butoxycarbonyl pent-4-yn-1-yl (1-(diphenoxyphosphoryl)-4-guanidinobutyl) carbamate (32a) General procedure ***G*** with **14b** (173 mg, 0.32 mmol) to yield **32a** (128 mg, 0.19 mmol, 60% yield). ^{1}HNMR (400 MHz, $CDCl_3$) δ (ppm) 11.49 (s, 1H), 8.37 (t, *J* = 5.27 Hz, 1H), 7.31 (q, *J* = 8.0 Hz, 4H), 7.18 (t, *J* = 6.7 Hz, 4H), 7.13 (d, *J* = 8.5 Hz, 2H), 5.42 (d, *J* = 10.3 Hz, 1H), 4.56-4.43 (m, 1H), 4.25-4.13 (m, 2H), 3.59-3.36 (m, 2H), 2.26 (dt, *J* = 7.0, 2.6 Hz, 2H), 2.16 -2.04 (m, 1H), 1.97 (t, *J* = 2.6 Hz, 1H), 1.88-1.78 (m, 4H), 1.48 (s, 9H), 1.47 (s, 9H). MS (ESI) *m/z* 673.1 $[M+H]^+$.

Pent-4-yn-1-yl (1-(diphenoxyphosphoryl)-4-guanidinobutyl)carbamate trifluoroacetate salt (32b) General procedure ***D*** with **32a** (34 mg, 0.05 mmol) to yield **32b** (29 mg, 0.05 mmol, 98% yield). ^{1}H NMR (400 MHz, Methanol-d_4) δ (ppm) 7.85 (d, *J* = 9.8 Hz, 1H), 7.57 (t, *J* = 5.4 Hz, 1H), 7.37 (t, *J* = 7.9 Hz, 4H), 7.26-7.19 (m, 2H), 7.16 (d, *J* = 8.2 Hz, 4H), 4.5-4.36 (m, 1H), 4.23-4.10 (m, 2H), 3.28-3.19 (m, 2H), 2.30-2.23 (m, 3H), 2.16-2.05 (m, 1H), 1.97-1.65 (m, 5H). ^{13}C NMR (100 MHz, Methanol-d_4) δ (ppm) 157.4, 157.2, 150.2, 150.1, 150.0, 149.9, 129.6, 129.6, 125.5, 125.4, 120.4, 120.4, 120.2, 120.1, 82.4, 68.9, 63.8, 40.3, 27.8, 26.0, 25.0, 25.0, 14.3. MS (ESI) *m/z* 473.1 $[M+H]^+$. HRMS for $C_{23}H_{29}N_4O_5P_1$, mass calculated 473.1948, mass measured 473.1940.

N, N'-bis-tert-butoxycarbonyl pent-4-yn-1-yl (1-(bis(4-chlorophenoxy)phosphoryl)-4-guanidinobutyl) carbamate (33a) General procedure ***G*** with **15b** (150 mg, 0.245 mmol) to yield **33a** (137 mg, 0.185 mmol, 75% yield). ^{1}H NMR (400 MHz, $CDCl_3$) δ 11.48 (s, 1H), 8.37 (t, *J* = 5.5 Hz, 1H), 7.28 (t, *J* = 7.1 Hz, 4H), 7.11 (dd, *J* = 19.9, 8.0 Hz, 4H), 5.63 (d, *J* = 10.2 Hz, 1H), 4.56-4.42 (m, 1H), 4.19 (t, *J* = 6.2 Hz, 2H), 3.63-3.50 (m, 1H), 3.45-3.33 (m, 1H), 2.27 (td, *J* = 7.0, 2.6 Hz, 2H), 2.13-2.01 (m, 1H), 1.98 (t, *J* = 2.6 Hz, 1H), 1.89 – 1.71 (m, 5H), 1.48 (s, 9H), 1.46 (s, 9H). MS (ESI) *m/z* 763.3 $[M+Na]^+$.

Pent-4-yn-1-yl (1-(bis(4-chlorophenoxy)phosphoryl)-4-guanidinobutyl)carbamate trifluoroacetate salt (33b) General procedure ***D*** with **33a** (30 mg, 0.04 mmol) to yield **33b** (21 mg, 0.039 mmol, 96% yield). ^{1}H NMR (400 MHz, Methanol-d_4) δ 7.84 (d, *J* = 9.8 Hz, 1H), 7.39 (d, *J* = 8.9 Hz, 4H), 7.24 – 7.13 (m, 4H), 4.51 – 4.33 (m, 1H), 4.16 (q, *J* = 6.3 Hz, 2H), 3.31 – 3.17 (m, 2H), 2.30 – 2.24 (m, 3H), 2.16 – 2.02 (m, 1H), 1.97 – 1.65 (m, 5H). ^{13}C NMR (100 MHz, Methanol-d_4) δ (ppm) 158.6, 150.3, 150.1, 150.0, 149.9, 132.3, 132.1, 131.0, 131.0, 123.4, 123.3, 123.1, 123.0, 83.8, 70.2, 65.2, 41.6, 30.9, 30.8, 29.2, 27.4, 26.3, 26.2, 15.7. MS (ESI) *m/z* 541.1 $[M+H]^+$. HRMS for $C_{23}H_{27}N_4O_5Cl_2P_1$, mass calculated 541.1169, mass measured 541.1142.

N, N'-bis-tert-butoxycarbonyl pent-4-yn-1-yl (1-(diphenoxyphosphoryl)-2-(4-guanidinophenyl)ethyl) carbamate (34a) General procedure ***G*** with **16b** (1.21 g, 2.350 mmol) to yield **34a** (910 mg, 1.26 mmol, 54% yield). ^{1}H NMR (400 MHz, DMSO-d_6) δ 11.43 (s, 1H), 9.98 (s, 1H), 8.04 (d, *J* = 9.6 Hz, 1H), 7.49 (d, *J* = 8.5 Hz, 2H), 7.45–7.37 (m, 4H), 7.29 (d, *J* = 8.5 Hz, 2H), 7.27–7.16 (m, 6H), 4.53–4.40 (m, 1H), 3.98–3.85 (m, 2H), 3.24 (d, *J* = 14.1 Hz, 1H), 3.03–2.91 (m, 1H), 2.77 (t, *J* = 2.6 Hz, 1H), 2.13 (td, *J* = 7.1, 2.3 Hz, 2H), 1.64–1.55 (m, 2H), 1.51 (s, 9H), 1.41 (s, 9H). MS (ESI) *m/z* 601.3 $[M+Na]^+$.

Pent-4-yn-1-yl (1-(diphenoxyphosphoryl)-2-(4-guanidinophenyl)ethyl)carbamate trifluoroacetate salt (34b) General procedure ***D*** with **34a** (70 mg, 0.097 mmol) to yield **34b** (50 mg, 0.096 mmol, 99% yield). ^{1}H NMR (400 MHz, $CDCl_3$) δ (ppm) 9.80 (s, 1H), 7.36 – 7.24 (m, 7H), 7.23–7.12 (m, 6H), 7.08 (t, *J* = 6.9 Hz, 5H), 5.71 (d, *J* = 10.2 Hz, 1H), 4.77–4.62 (m, 1H), 4.12–3.93 (m, 2H), 3.41–3.27 (m, 1H), 3.06 (dd, *J* = 24.5, 10.4 Hz, 1H), 2.15 (td, *J* = 6.9, 2.4 Hz, 2H), 1.95 (t, *J* = 2.5

Hz, 1H), 1.71 (p, *J* = 6.6 Hz, 2H). ^{13}C NMR (101 MHz, $CDCl_3$) δ (ppm) 156.6, 156.1, 156.0, 150.0, 149.9, 149.7, 149.6, 136.2, 133.0, 131.1, 130.0, 129.9, 125.9, 125.8, 125.7, 120.5, 120.5, 120.3, 120.3, 82.9, 77.2, 69.2, 64.2, 27.6, 14.9. MS (ESI) *m/z* 521.3 $[M+H]^+$. HRMS for $C_{27}H_{29}N_4O_5P_1$, mass calculated 521.1948, mass measured 521.1936.

N,N'*-bis-*tert*-butyloxycarbonyl pent-4-yn-1-yl (1-(bis(4-acetamidophenoxy)phosphoryl)-2-(4-guanidinophenyl)ethyl) carbamate (35a)** General procedure ***G with **17b** (68.5 mg, 0.097 mmol) to yield **35a** (80 mg, 0.096 mmol, 99% yield). ^1H NMR (400 MHz, DMSO-d_6) δ (ppm) 11.43 (s, 1H), 9.98 (s, 1H), 8.04 (d, *J* = 9.6 Hz, 1H), 7.49 (d, *J* = 8.5 Hz, 2H), 7.45–7.37 (m, 4H), 7.29 (d, *J* = 8.5 Hz, 2H), 7.27–7.16 (m, 6H), 4.53–4.40 (m, 1H), 3.98–3.85 (m, 2H), 3.24 (d, *J* = 14.1 Hz, 1H), 3.03–2.91 (m, 1H), 2.77 (t, *J* = 2.6 Hz, 1H), 2.13 (td, *J* = 7.1, 2.3 Hz, 2H), 1.64–1.55 (m, 2H), 1.51 (s, 9H), 1.41 (s, 9H). MS (ESI) *m/z* 835.0 $[M+H]^+$.

Pent-4-yn-1-yl (1-(bis(4-acetamidophenoxy)phosphoryl)-2-(4-guanidinophenyl)ethyl) carbamate trifluoroacetate salt (35b) General procedure ***D*** with **35a** (70 mg, 0.084 mmol) to yield **35b** (51 mg, 0.080 mmol, 96% yield). ^1H NMR (400 MHz, Methanol-d_4) δ (ppm) 7.55 (m, 4H), 7.40 (d, *J* = 8.4 Hz, 2H), 7.22 (d, *J* = 8.4 Hz, 2H), 7.13 (m, 4H), 4.68-4.54 (m, 1H), 4.02 (m, 1H), 3.40 (m, 1H), 3.12 (m, 1H), 2.23 (t, *J* = 2.8 Hz, 1H), 2.18 (m, 2H), 2.11 (s, 6H), 1.71 (m, 2H). ^{13}C NMR (100 MHz, Methanol-d_4) δ (ppm) 170.3, 157.1, 157.0, 156.6, 146.1, 146.0, 145.8, 145.7, 136.4, 136.3, 136.2, 133.6, 130.5, 125.2, 121.0, 120.9, 120.7, 120.6, 120.4, 120.3, 82.4, 68.8, 63.6, 34.5, 27.8, 22.4, 14.2. MS (ESI) *m/z* 635.3 $[M+H]^+$. HRMS for $C_{31}H_{35}N_6O_{57}P_1$, mass calculated 635.2378, mass measured 635.2382.

N,N'*-bis-*tert*-butyloxycarbonyl pent-4-yn-1-yl (1-(bis(4-chlorophenoxy)phosphoryl)-2-(4-guanidinophenyl) ethyl) carbamate (36a)** General procedure ***G with **18b** (0.170 g, 0.291 mmol) to yield **36a** (165.4 mg, 0.209 mmol, 81% yield). ^1H NMR (400 MHz, $CDCl_3$) δ (ppm) 11.63 (br s, 1H), 10.31 (br s, 1H), 7.54 (d, *J* = 8.5 Hz, 2H), 7.29 (d, *J* = 9.3 Hz, 2H), 7.23 (d, *J* = 7.3 Hz, 2H), 7.17 (d, *J* = 7.3 Hz, 2H), 7.11 (d, *J* = 8.1 Hz, 2H), 7.03 (d, *J* = 8.0 Hz, 2H), 5.71-5.38 (m, 1H), 4.83-4.63 (m, 1H), 4.14-3.94 (m, 2H), 3.35-3.21 (m, 1H), 2.99 (dd, *J* = 12.3, 10.2 Hz, 1H), 2.15 (br s, 2H), 1.98 (t, *J* = 2.6 Hz, 1H), 1.70 (p, *J* = 6.0 Hz, 2H), 1.52 (s, 9H), 1.49 (s, 9H). MS (ESI) *m/z* 791.2 $[M+H]^+$.

Pent-4-yn-1-yl (1-(bis(4-chlorophenoxy)phosphoryl)-2-(4-guanidinophenyl)ethyl)carbamate trifluoroacetate salt (36b) General procedure ***D*** with **36a** (52.7 mg, 0.067 mmol) to yield **36b** (47 mg, 0,067 mmol, 100% yield). ^1H NMR (400 MHz, Methanol-d_4) δ (ppm) 7.44-7.34 (m, 6H), 7.28-7.15 (m, 6H), 4.64 (ddd, *J* = 15.8, 13.5, 3.8 Hz, 1H), 4.02 (t, *J* = 6.2 Hz, 2H), 3.40 (dt, *J* = 14.2, 4.3 Hz, 1H), 3.13-3.00 (m, 1H), 2.25 (t, *J* = 2.8 Hz, 1H), 2.19 (td, *J* = 7.1, 2.4 Hz, 2H), 1.70 (p, *J* = 6.4 Hz, 2H). ^{13}C NMR (100 MHz, Methanol-d_4) δ (ppm) 161.3, 158.1, 150.2, 149.9, 137.5, 135.1, 132.3, 131.9, 131.1, 126.6, 123.4, 123.1, 83.8, 70.3, 65.0, 51.3, 35.8, 29.2, 27.8, 15.7. MS (ESI) *m/z* 589.1 $[M+H]^+$. HRMS for $C_{27}H_{27}N_4O_5Cl_2P_1$, mass calculated 589.1169, mass measured 589.1188.

N,N'*-bis-*tert*-butyloxycarbonyl pent-4-yn-1-yl ((diphenoxyphosphoryl)(4-guanidinophenyl)methyl)carbamate (37a)** General procedure ***G with **19b** (1.75 g, 3.49 mmol) to yield **37a** (1.38 g, 1.953 mmol, 56% yield). ^1H NMR (400 MHz, $CDCl_3$) δ (ppm) 11.62 (s, 1H), 10.38 (s, 1H), 7.64 (d, *J* = 8.0 Hz, 2H), 7.46 (d, *J* = 8.3 Hz, 2H), 7.31 (t, *J* = 8.0 Hz, 2H), 7.24 (t, *J* = 7.9 Hz, 2H), 7.21-7.04 (m, 4H), 6.91 (d, *J* = 8.3 Hz, 2H), 5.70 (dd, *J* = 9.9, 3.0 Hz, 1H), 5.52 (dd, *J* = 22.8, 5.5 Hz, 1H), 4.26-4.08 (m, 2H), 2.25 (t, *J* = 6.2 Hz, 2H), 1.96 (t, *J* = 2.5 Hz, 1H), 1.81 (p, *J* = 6.8 Hz, 2H), 1.53 (s, 9H), 1.50 (s, 9H). MS (ESI) *m/z* 729.3 $[M+Na]^+$.

Pent-4-yn-1-yl ((diphenoxyphosphoryl)(4-guanidinophenyl)methyl)carbamate trifluoroacetate salt (37b) General procedure ***D*** with **37a** (57 mg, 0.081 mmol) to yield **37b** (50.6 mg, 0.081 mmol, 100% yield). ^{1}H NMR (400 MHz, Methanol-*d$_4$*) δ (ppm) 8.65 (br d, *J* = 9.6 Hz, 1H), 7.69 (dd, *J* = 8.2, 2.0 Hz, 2H), 7.29-7.38 (m, 6H), 7.21 (t, *J* = 7.6 Hz, 2H), 7.10 (d, *J* = 8.2 Hz, 2H), 7.05 (d, *J* = 7.8 Hz, 2H), 5.74-5.59 (m, 1H), 4.25-4.08 (m, 2H), 2.32-2.22 (m, 3H), 1.82 (p, *J* = 6.5 Hz, 2H). ^{13}C NMR (100 MHz, Methanol-*d$_4$*) δ (ppm) 158.0, 151.4, 136.7, 134.9, 131.0, 126.9, 126.5, 121.6, 83.8, 70.3, 65.3, 29.2, 15.7. MS (ESI) *m/z* 507.2 [M+H]$^+$. HRMS for $C_{26}H_{27}N_4O_5P_1$, mass calculated 507.1792, mass measured 507.1796.

N,N'*-bis-*tert*-butyloxycarbonyl pent-4-yn-1-yl ((bis(4-acetamidophenoxy)phosphoryl)(4-guanidinophenyl) methyl) carbamate (38a)** General procedure ***G with **20b** (680 mg, 1.173 mmol) to yield **38a** (765 mg, 0.932 mmol, 79% yield). ^{1}H NMR (400 MHz, Methanol-d$_4$) δ (ppm) 7.53 (d, *J* = 8.4 Hz, 2H), 7.57-7.46 (m, 6H), 7.04 (d, *J* = 9.1 Hz, 2H), 6.94 (d, *J* = 8.7 Hz, 2H), 5.59 (d, *J* = 22.3 Hz, 1H), 4.24-4.09 (m, 2H), 2.30-2.22 (m, 3H), 2.09 (d, *J* = 3.4 Hz, 6H), 1.81 (p, *J* = 6.6 Hz, 2H), 1.57 (s, 9H), 1.45 (s, 9H). MS (ESI) *m/z* 821.5 [M+H]$^+$.

Pent-4-yn-1-yl ((bis(4-acetamidophenoxy)phosphoryl)(4-guanidinophenyl) methyl) carbamate (38b) General procedure ***D*** with **38a** (100 mg, 0.122 mmol) to yield **38b** (76 mg, 0.122 mmol, 99% yield). ^{1}H NMR (400 MHz, Methanol-d$_4$) δ (ppm) 7.67 (d, *J* = 6.9 Hz, 2H), 7.51 (dd, *J* = 8.8, 5.2 Hz, 4H), 7.33 (d, *J* = 8.3 Hz, 2H), 7.02 (dd, *J* = 22.3, 8.7 Hz, 4H), 5.64 (d, *J* = 22.8 Hz, 1H), 4.24-4.06 (m, 2H), 2.31-2.23 (m, 3H) 2.10 (s, 6H), 1.86-1.73 (m, 2H). ^{13}C NMR (100 MHz, Methanol-*d$_4$*) δ (ppm) 170.2, 156.9, 156.8, 156.5, 146.1, 146.0, 145.8, 145.7, 136.3, 136.2, 135.3, 135.3, 133.4, 129.8, 129.7, 125.1, 121.0, 120.9, 120.4, 120.4, 120.3, 120.3, 82.4, 68.9, 53.0, 51.4, 27.8, 22.3, 14.3. MS (ESI) *m/z* 621.3 [M+H]$^+$. HRMS for $C_{30}H_{33}N_6O_7P_1$, mass calculated 621.2221, mass measured 621.2243.

N,N'*-bis-*tert*-butyloxycarbonyl pent-4-yn-1-yl ((bis(4-chlorophenoxy)phosphoryl)(4-guanidinophenyl)methyl) carbamate (39a)** General procedure ***G with **21b** (670 mg, 1.25 mmol) to yield **39a** (970 mg, 1.251 mmol, 100% yield). ^{1}H NMR (400 MHz, $CDCl_3$) δ (ppm) 11.62 (s, 1H), 10.39 (s, 1H), 7.64 (d, *J* = 8.4 Hz, 2H), 7.43 (d, *J* = 7.1 Hz, 2H), 7.27 (d, *J* = 8.8 Hz, 2H), 7.20 (d, *J* = 8.4 Hz, 2H), 7.05 (d, *J* = 8.4 Hz, 2H), 6.82 (d, *J* = 8.3 Hz, 2H), 5.68 (dd, *J* = 10.2, 3.0 Hz, 1H), 5.51 (dd, *J* = 21.8, 9.9 Hz, 1H), 4.26-4.08 (m, 2H), 2.29-2.20 (m, 2H), 1.97 (t, *J* = 2.6 Hz, 1H), 1.87-1.76 (m, 2H), 1.53 (s, 9H), 1.50 (s, 9H). MS (ESI) *m/z* 777.2 [M+H]$^+$.

Pent-4-yn-1-yl ((bis(4-chlorophenoxy)phosphoryl)(4-guanidinophenyl)methyl)carbamate (39b) General procedure ***D*** with **39a** (100 mg, 0.129 mmol) to yield **39b** (70 mg, 0.121 mmol, 94% yield). ^{1}H NMR (400 MHz, Methanol-*d$_4$*) δ (ppm) 7.67 (d, *J* = 6.8 Hz, 2H), 7.42–7.29 (m, 6H), 7.13 (d, *J* = 8.6 Hz, 2H), 7.06 (d, *J* = 8.7 Hz, 2H), 5.69 (d, *J* = 22.8 Hz, 1H), 4.24–4.07 (m, 2H), 2.31-2.24 (m, 3H), 1.87–1.71 (m, 1H), 1.18 (t, *J* = 7.0 Hz, 2H). ^{13}C NMR (100 MHz, Methanol-*d$_4$*) δ (ppm) 158.4, 156.8, 156.5, 135.4, 133.07, 129.8, 129.7, 129.6, 129.6, 125.1, 121.8, 121.7, 121.7, 121.6, 68.9, 64.0, 53.0, 51.5, 29.5, 27.7, 14.3. MS (ESI) *m/z* 575.1 [M+H]$^+$. HRMS for $C_{26}H_{25}N_4O_5Cl_2P_1$, mass calculated 575.1012, mass measured 575.1030.

N,N'*-bis-*tert*-butyloxycarbonyl pent-4-yn-1-yl (1-(diphenoxyphosphoryl)-2-(4-(guanidinomethyl)phenyl) ethyl) carbamate (40a)** General procedure ***G with **22b** (0.995 g, 1.640 mmol) to yield **40a** (0.65 g, 0.885 mmol, 54% yield). ^{1}H NMR (400 MHz, $CDCl_3$) δ (ppm) 11.50 (s, 1H), 8.57 (t, *J* = 5.0 Hz, 1H), 7.38-7.07 (m, 14H), 5.02 (d, *J* = 10.5 Hz, 1H), 4.84-4.69 (m, 1H), 4.61 (d, *J* = 5.1 Hz, 1H), 4.14-4.00 (m, 2H), 3.39 (ddd, *J* = 14.4, 9.9, 4.5 Hz, 1H), 3.03 (dt, *J* = 14.5, 10.0 Hz, 1H), 2.15 (t, *J* = 6.7 Hz, 2H), 1.97 (t, *J* = 2.5 Hz, 1H), 1.71 (p, *J* = 6.5 Hz, 2H), 1.51 (s, 9H), 1.48 (s, 9H). MS (ESI) *m/z* 535.1 [(M-2Boc)+H]$^+$.

Pent-4-yn-1-yl (1-(diphenoxyphosphoryl)-2-(4-(guanidinomethyl)phenyl)ethyl)carbamate trifluoroacetate salt (40b) General procedure ***D*** with **40a** (50 mg, 0.068 mmol) to yield **40b** (41 mg, 0,063 mmol, 93% yield). ^{1}H NMR (400 MHz, Methanol-*d$_4$*) δ (ppm) 7.93 (d, *J* = 9.8 Hz, 1H), 7.36 (dd, *J* = 16.7, 7.7 Hz, 6H), 7.29 (d, *J* = 8.3 Hz, 2H), 7.23 (t, *J* = 7.1 Hz, 2H), 7.18 (d, *J* = 7.9 Hz, 4H), 4.69-4.51 (m, 1H), 4.38 (br s, 2H), 4.00 (oct, *J* = 6.0 Hz, 2H), 3.39 (dt, *J* = 13.8, 3.8 Hz, 1H), 3.04 (ddd, *J* = 26.2, 13.5, 8.9 Hz, 1H), 2.25 (t, *J* = 2.7 Hz, 1H), 2.17 (td, *J* = 7.2, 2.7 Hz, 2H), 1.69 (p, *J* = 6.8 Hz, 2H). ^{13}C NMR (100 MHz, Methanol-*d$_4$*) δ (ppm) 158.7, 158.4, 151.7, 151.4, 138.1, 137.9, 136.4, 131.0, 130.9, 128.6, 126.9, 126.8, 124.3, 121.9, 121.6, 83.9, 70.2, 64.9, 51.3, 45.7, 35.9, 29.2, 15.7. MS (ESI) *m/z* 535.2 [M+H]$^{+}$. HRMS for $C_{28}H_{31}N_4O_5P_1$, mass calculated 535.2105, mass measured 535.2081.

N,N'-bis-tert-butyloxycarbonyl pent-4-yn-1-yl ((1-carbamimidoylpiperidin-4-yl)(diphenoxyphosphoryl) methyl) carbamate (41a) General procedure ***G*** with **23b** (1.2 g, 2.10 mmol) to yield **41a** (0.95 g, 1.36 mmol, 65% yield). ^{1}H-NMR (400 MHz, $CDCl_3$) δ (ppm) 10.15 (br s, 1H), 7.39-7.30 (m, 4H), 7.24-7.10 (m, 6H), 5.23 (d, *J* = 10.5 Hz, 1H), 4.50 (ddd, *J* = 20.1, 10.6, 4.4 Hz, 1H), 4.22 (dt, *J* = 6.5, 2.2 Hz, 2H), 3.71 (q, *J* = 7.0 Hz, 1H), 3.02–2.86 (m, 2H), 2.28 (dt, *J* = 7.0, 2.6 Hz, 2H), 2.01 (t, *J* = 2.6 Hz, 1H), 1.89–1.77 (m, 3H), 1.71–1.54 (m, 2H), 1.50 (s, 18H), 1.24 (t, *J* = 7.0 Hz, 2H). MS (ESI) *m/z* 699.4 [M+H]$^{+}$.

Pent-4-yn-1-yl ((1-carbamimidoylpiperidin-4-yl)(diphenoxyphosphoryl)methyl)carbamate trifluoroacetate salt (41b) General procedure ***D*** with **41a** (75 mg, 0.107 mmol) to yield **41b** (52 mg, 0.104 mmol, 97% yield). ^{1}H NMR (400 MHz, Methanol-*d$_4$*) δ (ppm) 7.37 (t, *J* = 7.94 Hz, 4H), 7.26–7.20 (m, 2H), 7.19–7.14 (m, 4H), 4.43 (dd, *J* = 18.6, 6.6 Hz, 1H), 4.23–4.09 (m, 2H), 3.99–3.88 (m, 2H), 3.20–3.06 (m, 2H), 2.43–2.31 (m, 1H), 2.31-2.24 (m, 3H), 2.07 (d, *J* = 13.8 Hz), 1.86–1.76 (m, 2H), 1.65-1.46 (m, 2H). ^{13}C NMR (100 MHz, Methanol-*d$_4$*) δ (ppm) 157.5, 157.4, 156.2, 150.2, 150.1, 149.9, 149.8, 129.7, 129.6, 125.5, 125.4, 120.4, 120.4, 120.1, 120.1, 82.4, 68.8, 63.8, 52.9, 51.4, 45.3, 45.1, 36.3, 36.2, 28.7, 28.6, 27.8, 27.3, 27.2, 14.3. MS (ESI) *m/z* 499.3 [M+H]$^{+}$. HRMS for $C_{25}H_{31}N_4O_5P_1$, mass calculated 499.2105, mass measured 499.2098.

N-(2-(2-(2-(2-azidoethoxy)ethoxy)ethoxy)ethyl)-6-((4R,5S)-5-methyl-2-oxoimidazolidin-4-yl)hexanamide (S16) DIPEA (0.777 mL, 4.46 mmol) was added to a solution of 2-(2-(2-(2-azidoethoxy)ethoxy)ethoxy)ethan-1-amine (0.8 mL, 4.09 mmol), 6-((4R,5S)-5-methyl-2-oxoimidazolidin-4-yl)hexanoic acid (0.79 g, 3.72 mmol), EDC (0.85 g, 4.46 mmol), and HOBt (0.68 g, 4.46 mmol) in DMF (30 mL). The mixture was stirred at room temperature for 16h. The solvent was removed under reduced pressure by co-evaporation with toluene. The mixture was diluted with EtOAc (40 mL) and washed with 1 M HCl (30 mL). The aqueous layer was extracted with EtOAc (3 x 50 mL) and the combined organic layer was washed with saturated solution of $NaHCO_3$ and brine, dried over $MgSO_4$ and concentrated under reduced pressure. The crude product was purified by reverse phase chromatography (C18, MeCN in H_2O, 0/100 to 60/40) to yield **S16** (1.09 g, 2.63 mmol, 71% yield). ^{1}H NMR (400 MHz, $CDCl_3$) δ (ppm) 6.72 (br s, 1H), 6.00 (br s, 1H), 5.17 (br s, 1H), 3.79 (p, *J* = 6.9 Hz, 1H), 3.73-3.56 (m, 11H), 3.53 (t, *J* = 5.0 Hz, 2H), 3.41 (t, *J* = 5.0 Hz, 2H), 3.36 (t, *J* = 5.0 Hz, 2H), 2.16 (t, *J* = 7.3 Hz, 2H), 1.63 (p, *J* = 7.0 Hz, 2H), 1.51-1.15 (m, 6H), 1.08 (d, *J* = 6.4 Hz, 3H. MS (ESI) *m/z* 415.4 [M+H]$^{+}$.

2,5-dioxopyrrolidin-1-yl 5-((3aS,4S,6aR)-2-oxohexahydro-1H-thieno[3,4-d]imidazol-4-yl)pentanoate (S17) D-Biotin (100 mg, 0.41 mmol) and N-hydroxysuccinimide (56.5 mg, 0.49 mmol) were dissolved in anhydrous DMF (10 mL) at 70 °C. 1,3-Dicyclohexylcarbodiimide (101 mg, 0.49 mmol) was added, and the solution was stirred at room temperature for 16 h. The formed DCU was filtered off, and the solution was evaporated to dryness. The residue was taken up into boiling

isopropanol, and the solution was allowed to cool down to room temperature. The target compound was precipitated out, and the product was filtered off to yield **S17** (72 mg, 0.21 mmol, 52% yield). ^{1}H NMR (400 MHz, DMSO-d_6) δ (ppm) 6.40 (d, *J* = 24.3 Hz, 2H), 4.34–4.26 (m, 1H), 4.20–4.10 (m, 1H), 3.14–3.07 (m, 1H), 2.87–2.77 (m, 5H), 2.67 (t, *J* = 7.4 Hz, 2H), 2.58 (d, *J* = 12.4 Hz, 1H), 1.71–1.58 (m, 3H), 1.56–1.35 (m, 3H). MS (ESI) *m/z* 342.2 $[M+H]^+$.

N-(2-(2-(2-(2-azidoethoxy)ethoxy)ethoxy)ethyl)-5-((3aS,4S,6aR)-2-oxohexahydro-1H-thieno[3,4-d]imidazol-4-yl)pentanamide (S18) Triethylamine (0.059 mL, 0.422 mmol) was added to a solution of 1-Amino-11-azido-3,6,9-trioxaundecane (0.042 mL, 0.211 mmol) in DMF (35 mL), followed by the addition of **S17** (72 mg, 0.211 mmol). The resulting solution was stirred at room temperature for 16 h. The solvent was evaporated. The crude product was purified by reverse phase chromatography (C18, CH_3OH in H_2O, 0/100 to 100/0). The desired fractions were collected to yield **S18** (25 mg, 0.056 mmol, 14% yield). ^{1}H NMR (400 MHz, DMSO-d_6) δ (ppm) 4.42-4.31 (m, 1H), 4.19-4.12 (m, 1H), 3.62 (m, 2H), 3.53 (m, 8H), 3.38 (m, 4H), 3.18 (m, 2H), 3.09 (m, 1H), 2.82 (dd, *J* = 12.4, 5.2 Hz, 1H), 2.59 (d, *J* = 12.8 Hz, 1H), 2.07 (t, *J* = 7.2 Hz, 2H), 1.29-1.64 (m, 6H). MS (ESI) *m/z* 445.3 $[M+H]^+$.

3-(1-(18-((4R,5S)-5-methyl-2-oxoimidazolidin-4-yl)-13-oxo-3,6,9-trioxa-12-azaoctadecyl)-1H-1,2,3-triazol-4-yl)propyl (1-(diphenoxyphosphoryl)-2-phenylethyl)carbamate (42) General procedure ***H*** with **12** (112 mg, 0.241 mmol) and **S16** to yield **42** (68 mg, 0.077 mmol, 32% yield). ^{1}H NMR (400 MHz, Methanol-d_4) δ (ppm) 7.61 (s, 1H), 7.42-7.14 (m, 15H), 4.71-4.60 (m, 1H), 4.52 (t, *J* = 5.1 Hz, 2H), 4.01-3.91 (m, 2H) 3.86 (t, *J* = 5.0 Hz, 2H), 3.84-3.75 (m, 1H), 3.71-3.63 (m, 1H), 3.62-3.53 (m, 8H), 3.49 (t, *J* = 5.5 Hz, 2H), 3.39 (dt, *J* = 14.3, 4.3 Hz, 1H), 3.33 (t, *J* = 5.6 Hz, 2H), 3.05 (ddd, *J* = 14.3, 13.1, 9 Hz, 1H), 2.65 (t, *J* = 7.6 Hz, 2H), 2.18 (t, *J* = 7.6 Hz, 2H), 1.85 (p, *J* = 6.9 Hz, 2H), 1.61 (p, *J* = 6.9 Hz, 2H), 1.52-1.24 (m, 6H), 1.08 (d, *J* = 6.6 Hz, 3H).^{13}C NMR (100 MHz, Methanol-d_4) δ (ppm) 176.1, 166.1, 158.3, 151.6, 151.4, 147.8, 138.1, 131.0, 130.3, 129.5, 127.9, 126.8, 124.1, 121.8, 121.6, 121.5, 71.5, 71.4, 71.2, 70.5, 70.4, 69.4, 65.1, 57.3, 51.4, 51.3, 40.3, 36.9, 36.2, 31.1, 30.7, 30.2, 29.7, 27.1, 26.8, 22.6, 15.7. MS (ESI) *m/z* 878.5 $[M+H]^+$. HRMS for $C_{44}H_{60}N_7O_{10}P_1$, mass calculated 878.4212, mass measured 878.4178.

3-(1-(13-oxo-17-((3aS,4S,6aR)-2-oxohexahydro-1H-thieno[3,4-d]imidazol-4-yl)-3,6,9-trioxa-12-zaheptadecyl)-1H-1,2,3-triazol-4-yl)propyl (1-(diphenoxyphosphoryl)-2-phenylethyl)carbamate (43) General procedure ***H*** with **12** (52.1 mg, 0.112 mmol) and **S18** to yield **43** (35 mg, 0.039 mmol, 34% yield). ^{1}H NMR (400 MHz, Methanol-d_4) δ (ppm) 7.68 (s, 1H), 7.41-7.16 (m, 15H), 4.66 (ddd, *J* = 15.5, 12.0, 3.5 Hz, 1H), 4.52 (t, *J* = 5.0 Hz, 2H), 4.46 (dd, *J* = 8.0, 4.5 Hz, 1H), 4.26 (dd, *J* = 8.0, 4.5 Hz, 1H), 4.01–3.91 (m, 2H), 3.89–3.83 (m, 2H), 3.57 (d, *J* = 12.0 Hz, 8H), 3.50 (t, *J* = 5.5 Hz, 2H), 3.39 (ddd, *J* = 12.0, 8.0, 4.0 Hz, 1H), 3.34 (d, *J* = 5.5 Hz, 2H), 3.21-3.12 (m, 1H), 3.05 (ddd, *J* = 14.0, 12.0, 9.0 Hz, 1H), 2.89 (dd, *J* = 12.5, 5.0 Hz, 1H), 2.72-2.61 (m, 3H), 2.18 (t, *J* = 7.5 Hz, 2H), 1.91-1.80 (m, 2H), 1.76-1.50 (m, 4H), 1.46-1.34 (m, 2H). ^{13}C NMR (100 MHz, Methanol-d_4) δ (ppm) 176.1, 166.1, 158.4, 151.7, 151.5, 147.9, 138.1, 131.0, 130.9, 130.3, 129.6, 128.0, 126.8, 126.7, 124.1, 121.9, 121.8, 121.6, 121.5, 71.5, 71.5, 71.4, 71.2, 70.6, 70.4, 65.2, 63.3, 61.6, 57.0, 51.4, 51.3, 41.0, 40.3, 36.7, 36.3, 29.8, 29.8, 29.5, 26.8, 22.6. MS (ESI) m/z 908.5 $[M+H]^+$. HRMS for $C_{44}H_{58}N_7O_{10}S_1P_1$, mass calculated 908.3776, mass measured 908.3735.

3-(1-(18-((4R,5S)-5-methyl-2-oxoimidazolidin-4-yl)-13-oxo-3,6,9-trioxa-12-azaoctadecyl)-1H-1,2,3-triazol-4-yl)propyl (1-(diphenoxyphosphoryl)-2-methylpropyl)carbamate (44) General procedure ***H*** with **13** (90 mg, 0.217 mmol) and **S16** to yield **44** (29.8 mg, 0.036 mmol, 17% yield). ^{1}H NMR (400 MHz, Methanol-d_4) δ (ppm) 7.74 (s, 1H), 7.40-7.30 (m, 4H), 7.25-7.11 (m, 6H), 4.52 (t, *J* = 5.1 Hz, 2H), 4.32 (dd, *J* = 18.4, 5.8 Hz, 1H), 4.12 (qd, *J* = 6.1 Hz, 2H), 3.87 (t, *J* = 5.0 Hz, 2H), 3.84-3.76 (m, 1H), 3.72-3.63 (m, 1H), 3.61-3.54 (m, 8H), 3.50 (t, *J* = 5.4 Hz, 2H), 3.33 (t, *J* = 5.5 Hz, 2H),

2.78 (t, J = 7.8 Hz, 2H), 2.38 (septet, J = 6.6 Hz, 1H), 2.20 (t, J = 7.2 Hz, 2H), 1.99 (p, J = 7.2 Hz, 2H), 1.61 (p, J = 7.2 Hz, 2H), 1.52-1.25 (m, 8H), 1.15 (d, J = 6.6 Hz, 6H), 1.08 (d, J = 6.5 Hz, 3H). ^{13}C NMR (100 MHz, Methanol-d_4) δ (ppm) 176.2, 166.2, 159.1, 151.1, 130.9, 126.4, 124.3, 121.8, 121.6, 71.6, 71.5, 71.4, 71.2, 70.6, 70.3, 65.5, 57.1, 55.4, 52.8, 51.3, 40.3, 36.9, 30.8, 30.3, 30.2, 29.9, 26.9, 22.8, 19.1, 21.0, 15.8. MS (ESI) *m/z* 830.5 [M+H]$^+$. HRMS for $C_{40}H_{60}N_7O_{10}P_1$, mass calculated 830.4212, mass measured 830.4188.

3-(1-(18-((4R,5S)-5-methyl-2-oxoimidazolidin-4-yl)-13-oxo-3,6,9-trioxa-12-azaoctadecyl)-1H-1,2,3-triazol-4-yl)propyl (1-(diphenoxyphosphoryl)-4-guanidinobutyl)carbamate trifluoroacetate salt (45b) General procedure ***H*** with **32a** (16.23 mg, 0.024 mmol) and **S16**, followed by general procedure ***D*** to yield **45b** (18 mg, 0.018 mmol, 75% yield). ^{1}H NMR (400 MHz, DMSO-d_6) 7.97 (d, J = 10.1 Hz, 1H), 7.81 (s, 1H), 7.74-7.60 (m, 2H), 7.38 (q, J = 7.8 Hz, 4H), 7.21 (q, J = 8.1 Hz, 2H), 7.15 (t, J = 8.9 Hz, 4H), 4.46 (t, J = 5.2 Hz, 2H), 4.33-4.21 (m, 3H), 4.11-3.96 (m, 3H), 3.78 (t, J = 5.2 Hz, 2H), 3.59 (t, J = 6.8 Hz, 1H), 3.52-3.42 (m, 8H), 3.36 (t, J = 5.8 Hz, 2H), 3.16 (t, J = 5.4 Hz, 3H), 2.66 (t, J = 7.6 Hz, 2H), 2.04 (t, J = 7.3 Hz, 2H), 1.99-1.92 (m, 1H), 1.88 (t, J = 7.3 Hz, 2H), 1.83-1.28 (m, 9H), 1.28-1.12 (m, 9H), 1.11-1.05 (m, 1H), 1.02 (d, J = 6.4 Hz, 1H), 0.94 (d, J = 6.4Hz, 2H). ^{13}C NMR (100 MHz, DMSO-d_6) δ (ppm) 172.7, 163.4, 157.0, 150.6, 150.2, 146.4, 130.4, 130.3, 130.3, 125.9, 125.7, 122.7, 121.1, 121.1, 120.8, 120.8, 70.2, 70.1, 70.1, 70.0, 69.6, 69.2, 64.4, 55.4, 50.6, 49.7, 49.3, 47.7, 38.9, 38.7, 35.7, 35.6, 30.0, 29.5, 29.1, 29.0, 26.2, 26.0, 25.6, 21.9, 15.9. MS (ESI) *m/z* 887.6 [M+H]$^+$. HRMS for $C_{41}H_{63}N_{10}O_{10}P_1$, mass calculated 887.4539, mass measured 887.4531.

3-(1-(18-((4R,5S)-5-methyl-2-oxoimidazolidin-4-yl)-13-oxo-3,6,9-trioxa-12-azaoctadecyl)-1H-1,2,3-triazol-4-yl)propyl (1-(bis(4-chlorophenoxy)phosphoryl)-4-guanidinobutyl)carbamate trifluoroacetate salt (46b) General procedure ***H*** with **33a** (42 mg, 0.101 mmol) and **S16**, followed by general procedure ***D*** to yield **46b** (57.2 mg, 0.060 mmol, 60% yield). ^{1}H NMR (400 MHz, Methanol-d_4) δ (ppm) 7.85 (d, J = 9.8 Hz, 1H), 7.81 (s, 1H), 7.38 (d, J = 8.9 Hz, 4H), 7.18 (t, J = 7.4 Hz, 4H), 4.55 (t, J = 5.1 Hz, 2H), 4.50–4.38 (m, 1H), 4.12 (t, J = 6.3 Hz, 2H), 3.88 (t, J = 5.1 Hz, 2H), 3.85–3.77 (m, 1H), 3.68 (q, J = 7.5 Hz, 1H), 3.60 (s, 4H), 3.57 (s, 4H), 3.51 (t, J = 5.6 Hz, 2H), 3.34 (t, J = 5.6 Hz, 2H), 3.30–3.21 (m, 2H), 2.78 (t, J = 7.6 Hz, 2H), 2.18 (t, J = 7.5 Hz, 2H), 2.15–2.04 (m, 1H), 1.99 (p, J = 6.5 Hz, 2H), 1.95–1.80 (m, 2H), 1.80–1.67 (m, 1H), 1.61 (p, J = 7.2 Hz, 2H), 1.53–1.42 (m, 2H), 1.42–1.26 (m, 4H), 1.09 (d, J = 6.5 Hz, 3H). ^{13}C NMR (100 MHz, Methanol-d_4) δ (ppm) 174.9, 157.2, 148.5, 129.7, 122.0, 121.7, 121.7, 70.1, 70.1, 70.0, 69.9, 69.2, 69.0, 64.3, 56.0, 51.3, 50.0, 40.3, 39.0, 35.5, 29.5, 29.3, 28.8, 28.5, 26.0, 25.8, 25.4, 25.0, 24.8, 21.3, 14.2. MS (ESI) *m/z* 955.5 [M+H]$^+$. HRMS for $C_{41}H_{61}N_{10}O_{10}Cl_2P_1$, mass calculated 955.3760, mass measured 955.3773.

3-(1-(18-((4R,5S)-5-methyl-2-oxoimidazolidin-4-yl)-13-oxo-3,6,9-trioxa-12-azaoctadecyl)-1H-1,2,3-triazol-4-yl)propyl (5-amino-1-(diphenoxyphosphoryl)pentyl)carbamate trifluoroacetate salt (47b) General procedure ***H*** with **29a** (162 mg, 0.297 mmol) and **S16**, followed by general procedure ***D*** to yield **47b** (242 mg, 0.246 mmol, 83% yield). ^{1}H NMR (400 MHz, Methanol-d_4) δ (ppm) 7.89 (s, 1H), 7.38 (t, J = 7.6 Hz, 4H), 7.27-7.21 (m, 2H), 7.21-7.13 (m, 4H), 4.56 (t, J = 4.9 Hz, 2H), 4.50-4.37 (m, 1H), 4.21-4.07 (m, 2H), 3.90 (t, J = 4.4 Hz, 2H), 3.87-3.78 (m, 1H), 3.75-3.65 (m, 1H), 3.65-3.56 (m, 8H), 3.53 (t, J = 5.7 Hz, 2H), 3.36 (t, J = 5.8 Hz, 2H), 2.97 (t, J = 7.3 Hz, 2H), 2.87-2.73 (m, 2H), 2.20 (t, J = 7.7 Hz, 2H), 2.16-1.85 (m, 4H), 1.84-1.25 (m, 12H), 1.11 (d, J = 6.6 Hz, 3H). ^{13}C NMR (100 MHz, Methanol-d_4) δ (ppm) 131.0, 126.8, 126.7, 121.8, 121.6, 71.5, 71.4, 71.2, 70.6, 70.3, 65.5, 57.4, 49.4, 49.2, 40.5, 40.3, 36.9, 30.7, 30.2, 29.8, 27.8, 27.2, 26.8, 23.7, 15.6. MS (ESI) *m/z* 859.5 [M+H]$^+$. HRMS for $C_{41}H_{63}N_8O_{10}P_1$, mass calculated 859.4477, mass measured 859.4462.

3-(1-(13-oxo-17-((3aS,4S,6aR)-2-oxohexahydro-1H-thieno[3,4-d]imidazol-4-yl)-3,6,9-trioxa-12-azaheptadecyl)-1H-1,2,3-triazol-4-yl)propyl (5-amino-1-(diphenoxyphosphoryl)pentyl) carbamate trifluoroacetate salt (48b) General procedure ***H*** with **29a** (0.163 g, 0.367 mmol) and **S18**, followed by general procedure ***D*** to yield **48b** (121.1 mg, 0.121 mmol, 33% yield). ^{1}H NMR (400 MHz, Methanol-d_4) δ (ppm) 7.98-7.80 (m, 1H), 7.38 (t, *J* = 7.7 Hz, 4H), 7.28-7.12 (m, 6H), 4.56 (t, *J* = 4.8 Hz, 2H), 4.53-4.37 (m, 2H), 4.31 (dd, *J* = 7.8, 4.4 Hz, 1H), 4.20-4.07 (m, 2H), 3.90 (t, *J* = 5.0 Hz, 2H), 3.64-3.57 (m, 8H), 3.53 (t, *J* = 5.6 Hz, 2H), 3.36 (t, *J* = 5.6 Hz, 2H), 3.24-3.17 (m, 1H), 3.02-2.89 (m, 4H), 2.87-2.75 (m, 2H), 2.21 (t, *J* = 7.4 Hz, 2H), 2.16-1.84 (m, 4H), 1.84-1.37 (m, 10H). ^{13}C NMR (100 MHz, Methanol-d_4) δ (ppm) 176.0, 158.7, 151.5, 151.4, 131.0, 126.8, 121.6, 121.5, 71.5, 71.4, 71.2, 70.5, 70.3, 65.5, 63.4, 61.6, 57.0, 51.7, 41.1, 40.5, 40.3, 36.7, 29.7, 29.5, 27.8, 26.8, 23.7, 22.7. MS (ESI) *m/z* 889.5 $[M+H]^+$. HRMS for $C_{41}H_{61}N_8O_{10}S_1P_1$, mass calculated 889.4042, mass measured 889.4017.

3-(1-(18-((4R,5S)-5-methyl-2-oxoimidazolidin-4-yl)-13-oxo-3,6,9-trioxa-12-azaoctadecyl)-1H-1,2,3-triazol-4-yl)propyl (5-amino-1-(bis(4-acetamidophenoxy)phosphoryl)pentyl)carbamate trifluoroacetate salt (49b) General procedure ***H*** with **30a** (40 mg, 0.061 mmol) and **S16**, followed by general procedure ***D*** to yield **49b** (34.5 mg, 0.032 mmol, 53% yield). ^{1}H NMR (400 MHz, Methanol-d_4) δ (ppm) 7.81 (s, 1H), 7.54 (d, *J* = 8.7 Hz, 4H), 7.15-7.08 (m, 4H), 4.53 (t, *J* = 4.9 Hz, 2H), 4.40 (ddd, *J* = 16.5, 13.4, 3.5 Hz, 1H), 4.20-4.04 (m, 2H), 3.87 (t, *J* = 4.9 Hz, 2H), 3.80 (p, *J* = 7.1 Hz, 1H), 3.73-3.45 (m, 11H), 3.33 (t, *J* = 5.4 Hz, 2H), 2.95 (t, *J* = 6.9 Hz, 2H), 2.78 (br s, 2H), 2.24-2.13 (m, 2H), 2.11 (s, 6H), 2.08-1.84 (m, 4H), 2.07-1.16 (m, 15H). ^{13}C NMR (100 MHz, Methanol-d_4) δ (ppm) 176.2, 171.6, 137.7, 122.3, 122.0, 121.8, 71.5, 71.4, 71.2, 70.6, 70.4, 65.6, 57.4, 52.7, 51.3, 40.5, 40.3, 33.9, 30.7, 30.2, 29.9, 27.1, 26.8, 23.8, 15.6. MS (ESI) *m/z* 973.7 $[M+H]^+$. HRMS for $C_{45}H_{69}N_{10}O_{12}P_1$, mass calculated 973.4907, mass measured 973.4898.

3-(1-(18-((4R,5S)-5-methyl-2-oxoimidazolidin-4-yl)-13-oxo-3,6,9-trioxa-12-azaoctadecyl)-1H-1,2,3-triazol-4-yl)propyl (1-(diphenoxyphosphoryl)-2-(4-guanidinophenyl)ethyl)carbamate trifluoroacetate salt (50b) General procedure ***H*** with **34a** (50 mg, 0.069 mmol) and **S16**, followed by general procedure ***D*** to yield **50b** (49.3 mg, 0.052 mmol, 76% yield). ^{1}H NMR (400 MHz, Methanol-d_4) δ (ppm) 8.01 (d, *J* = 9.9 Hz, 1H), 7.75 (s, 1H), 7.43 (d, *J* = 8.3 Hz, 2H), 7.37 (t, *J* = 7.3 Hz, 4H), 7.21 (m, 8H), 4.71–4.57 (m, 1H), 4.53 (t, *J* = 5.0 Hz, 2H), 3.99 (td, *J* = 6.1, 2.4 Hz, 2H), 3.87 (t, *J* = 5.0 Hz, 2H), 3.84–3.76 (m, 1H), 3.71–3.66 (m, 2H), 3.59 (s, 4H), 3.56 (s, 4H), 3.50 (t, *J* = 5.5 Hz, 2H), 3.42 (dt, *J* = 14.3, 4.4 Hz, 1H), 3.34 (d, *J* = 6.5 Hz, 2H), 3.09 (ddd, *J* = 14.0, 11.6, 9.1 Hz, 1H), 2.68 (t, *J* = 7.6 Hz, 2H), 2.18 (t, *J* = 7.4 Hz, 2H), 1.88 (p, *J* = 6.7 Hz, 2H), 1.61 (p, *J* = 6.9 Hz, 2H), 1.51–1.42 (m, 2H), 1.09 (d, *J* = 6.5 Hz, 3H). ^{13}C NMR (100 MHz, Methanol-d_4) δ (ppm) 176.2, 166.2, 158.5, 158.5, 158.0, 151.6, 151.5, 151.4, 151.3, 137.9, 137.7, 134.9, 131.9, 131.0, 126.9, 126.8, 126.6, 124.1, 121.8, 121.8, 121.6, 121.6, 71.5, 71.4, 71.4, 71.2, 70.5, 70.4, 65.4, 57.4, 52.7, 52.0, 51.3, 50.5, 49.8, 40.3, 36.9, 30.7, 30.2, 29.8, 27.1, 26.8, 22.6, 15.6. MS (ESI) *m/z* 935.6 $[M+H]^+$.

3-(1-(13-oxo-17-((3aS,4S,6aR)-2-oxohexahydro-1H-thieno[3,4-d]imidazol-4-yl)-3,6,9-trioxa-12-azaheptadecyl)-1H-1,2,3-triazol-4-yl)propyl(1-(diphenoxyphosphoryl)-2-(4-guanidinophenyl)ethyl) carbamate trifluoroacetate salt (51b) General procedure ***H*** with **34a** (50 mg, 0.069 mmol) and **S18**, followed by general procedure ***D*** to yield **51b** (41 mg, 0.042 mmol, 61% yield). ^{1}H NMR (400 MHz, Methanol-d_4) δ (ppm) 8.00 (d, *J* = 9.8 Hz, 1H), 7.75 (s, 1H), 7.43 (d, *J* = 8.3 Hz, 2H), 7.38 (t, *J* = 7.3 Hz, 4H), 7.26-7.16 (m, 8H), 4.70-4.57 (m, 1H), 4.53 (t, *J* = 5.0 Hz, 2H), 4.47 (dd, *J* = 7.8, 4.9 Hz, 1H), 4.28 (dd, *J* = 7.8, 4.4 Hz, 1H), 4.03-3.95 (m, 2H), 3.88 (t, *J* = 5.0 Hz, 2H), 3.60 (s, 4H), 3.57 (s, 4H), 3.51 (t, *J* = 5.5 Hz, 2H), 3.42 (td, *J* = 13.59, 4.21 Hz, 1H), 3.34-3.33 (m, 2H), 3.21-3.14 (m, 1H), 3.14-3.04 (m, 1H), 2.92 (dd, *J* = 13.0, 4.9 Hz, 1H), 2.70 (s, 1H), 2.68-2.65

(m, 2H), 2.19 (t, *J* = 7.4 Hz, 2H), 1.92-1.82 (m, 2H), 1.79-1.50 (m, 4H), 1.46-1.39 (m, 2H). ^{13}C NMR (100 MHz, Methanol-*d4*) δ (ppm) 176.1, 166.1, 158.5, 158.0, 151.7, 151.6, 151.4, 151.3, 138.0, 137.8, 134.9, 131.9, 131.0, 126.9, 126.8, 126.6, 121.8, 121.8, 121.6, 121.6, 71.5, 71.4, 71.4, 71.2, 70.5, 70.3, 65.4, 63.4, 61.6, 57.0, 51.4, 41.0, 40.3, 36.7, 29.8, 29.5, 26.8, 22.6, 15.4. MS (ESI) *m/z* 965.5 $[M+H]^+$. HRMS for $C_{45}H_{61}N_{10}O_{10}S_1P_1$, mass calculated 965.4103, mass measured 965.4106.

3-(1-(18-((4R,5S)-5-methyl-2-oxoimidazolidin-4-yl)-13-oxo-3,6,9-trioxa-12-azaoctadecyl)-1H-1,2,3-triazol-4-yl)propyl (1-(bis(4-acetamidophenoxy)phosphoryl)-2-(4-guanidinophenyl)ethyl)carbamate trifluoroacetate salt (52b) General procedure ***H*** with **35a** (59.6 mg, 0.144 mmol) and **S16**, followed by general procedure ***D*** to yield **52b** (37 mg, 0.036 mmol, 25% yield). ^{1}H NMR (400 MHz, Methanol-*d4*) δ (ppm) 7.98 (br d, *J* = 9.3 Hz, 1H), 7.75 (br s, 1H), 7.55 (dd, *J* = 9.1, 3.1 Hz, 4H), 7.44 (d, *J* = 8.4 Hz, 2H), 7.24 (d, *J* = 8.3 Hz, 2H), 7.16 (t, *J* = 9.5 Hz, 4H), 4.69-4.56 (m, 1H), 4.53 (t, *J* = 5.1 Hz, 2H), 4.01 (t, *J* = 6.2 Hz, 2H), 3.89 (t, *J* = 5.0 Hz, 2H), 3.80 (p, *J* = 7.3 Hz, 1H), 3.73-3.64 (m, 1H), 3.62-3.52 (m, 8H), 3.50 (t, *J* = 5.6 Hz, 2H), 3.40 (dt, *J* = 14.2, 4.5 Hz, 1H), 3.33 (t, *J* = 5.6 Hz, 2H), 3.07 (q, *J* = 11.9 Hz, 1H), 2.68 (t, *J* = 7.6 Hz, 2H), 2.19 (t, *J* = 7.6 Hz, 2H), 2.11 (s, 6H), 1.88 (p, *J* = 6.8 Hz, 2H), 1.60 (p, *J* = 7.3 Hz, 2H), 1.53-1.19 (m, 7H), 1.08 (d, *J* = 6.3 Hz, 3H). ^{13}C NMR (101 MHz, Methanol-*d4*) δ 176.2, 171.6, 158.0, 134.9, 132.0, 126.7, 122.3, 122.1, 121.8, 71.5, 71.4, 71.3, 70.6, 70.4, 65.4, 57.4, 52.7, 51.4, 40.3, 36.9, 30.8, 30.2, 29.9, 27.2, 26.9, 23.8, 22.7, 15.7. MS (ESI) *m/z* 1049.5 $[M+H]^+$. HRMS for $C_{49}H_{69}N_{12}O_{12}P_1$, mass calculated 1049.4968, mass measured 1049.4980.

3-(1-(13-oxo-17-((3aS,4S,6aR)-2-oxohexahydro-1H-thieno[3,4-d]imidazol-4-yl)-3,6,9-trioxa-12-azaheptadecyl)-1H-1,2,3-triazol-4-yl)propyl(1-(bis(4-acetamidophenoxy)phosphoryl)-2-(4-guanidinophenyl) ethyl)carbamate trifluoroacetate salt (53b) General procedure ***H*** with **35a** (150 mg, 0.180 mmol) and **S18**, followed by general procedure ***D*** to yield **53b** (143.8 mg, 0.120 mmol, 67% yield). ^{1}H NMR (400 MHz, Methanol-*d4*) δ (ppm) 7.98 (d, *J* = 9.8 Hz, 1H), 7.78 (s, 1H), 7.55 (dd, *J* = 2.3, 8.9 Hz, 4H), 7.41 (d, *J* = 8.2 Hz, 2H), 7.21 (d, *J* = 8.2 Hz, 2H), 7.14 (t, *J* = 8.5 Hz, 4H), 4.66-4.58 (m, 1H), 4.53 (t, *J* = 4.8 Hz, 2H), 4.47 (dd, *J* = 4.7, 7.6 Hz, 1H), 4.27 (dd, *J* = 4.4, 7.7 Hz, 1H), 3.98 (t, *J* = 5.5 Hz, 2H), 3.86 (t, *J* = 4.8 Hz, 2H), 3.72-3.48 (s, 8H), 3.49 (t, *J* = 5.3 Hz, 2H), 3.42-3.38 (m, 1H), 3.31-3.28 (m, 2H), 3.17-3.11 (m, 1H), 3.10-3.06 (m, 1H), 2.90 (dd, *J* = 4.9, 12.7 Hz, 1H), 2.71-2.65 (m, 3H), 2.18 (t, *J* = 7.3 Hz, 2H), 2.11 (s, 6H), 1.89-1.85 (m, 2H), 1.62-1.59 (m, 4H), 1.42-1.39 (m, 2H). ^{13}C NMR (101 MHz, Methanol-*d4*) δ (ppm) 176.1, 171.6, 166.1, 158.5, 157.9, 147.4, 147.2, 137.7, 137.6, 134.9, 131.9, 126.6, 124.1, 122.4, 122.0, 121.8, 71.5, 71.4, 71.3, 71.2, 70.5, 70.3, 65.4, 63.3, 61.6, 57.0, 51.5, 51.1, 41.0, 40.2, 36.7, 35.9, 29.7, 29.4, 26.8, 23.7, 22.6. MS (ESI) *m/z* 1079.5 $[M+H]^+$. HRMS for $C_{49}H_{67}N_{12}O_{12}S_1P_1$, mass calculated 1079.4532, mass measured 1079.4541.

3-(1-(18-((4R,5S)-5-methyl-2-oxoimidazolidin-4-yl)-13-oxo-3,6,9-trioxa-12-azaoctadecyl)-1H-1,2,3-triazol-4-yl)propyl (1-(bis(4-chlorophenoxy)phosphoryl)-2-(4-guanidinophenyl)ethyl)carbamate trifluoroacetate salt (54b) General procedure ***H*** with **36a** (156 mg, 0.198 mmol) and **S16**, followed by general procedure ***D*** to yield **54b** (86.8 mg, 0.077 mmol, 39% yield). ^{1}H NMR (400 MHz, Methanol-*d4*) δ (ppm) 8.03 (d, *J* = 9.8 Hz, 1H), 7.81 (br s, 1H), 7.48-7.35 (m, 6H), 7.25-7.15 (m, 6H), 4.65 (ddd, *J* = 15.2, 13.6, 3.6 Hz, 1H), 4.54 (t, *J* = 5.0 Hz, 2H), 3.98 (t, *J* = 5.7 Hz, 1H), 3.88 (t, *J* = 4.8 Hz, 2H), 3.81 (p, *J* = 6.8 Hz, 1H), 3.68 (q, *J* = 7.1 Hz, 1H), 3.64-3.53 (m, 8H), 3.50 (t, *J* = 5.7 Hz, 2H), 3.41 (dt, *J* = 14.3, 4.7 Hz, 1H), 3.33 (t, *J* = 5.7 Hz, 1H), 3.21 (q, *J* = 7.2 Hz, 1H), 3.14-3.02 (m, 1H), 2.68 (br t, *J* = 7.2 Hz, 2H), 2.18 (t, *J* = 7.7 Hz, 2H), 1.87 (br t, *J* = 5.6 Hz, 1H), 1.55-1.21 (m, 2H), 1.51-1.24 (m, 8H), 1.09 (d, *J* = 6.3 Hz, 3H). ^{13}C NMR (100 MHz, Methanol-*d4*) δ (ppm) 176.3, 158.4, 158.0, 150.1, 150.0, 135.1, 132.3, 132.0, 131.1, 126.6, 123.4,

123.2, 71.5, 71.4, 71.3, 70.6, 70.4, 65.5, 57.4, 52.8, 51.5, 51.3, 47.9, 40.3, 36.9, 30.8, 30.2, 29.8, 27.2, 26.9, 22.7, 15.7. MS (ESI) *m/z* 1005.5 $[M+H]^+$. HRMS for $C_{45}H_{61}N_{10}O_{10}Cl_2P_1$, mass calculated 1003.3760, mass measured 1003.3767.

3-(1-(18-((4R,5S)-5-methyl-2-oxoimidazolidin-4-yl)-13-oxo-3,6,9-trioxa-12-azaoctadecyl)-1H-1,2,3-triazol-4-yl)propyl (1-(diphenoxyphosphoryl) (4-guanidinophenyl)carbamate trifluoroacetate salt (55b) General procedure ***H*** with **37a** (150 mg, 0.212 mmol) and **S16**, followed by general procedure ***D*** to yield **55b** (82 mg, 0.089 mmol, 42% yield). ^{1}H NMR (400 MHz, Methanol-*d₄*) δ (ppm) 7.80 (s, 1H), 7.70 (d, *J* = 7.6 Hz, 2H),7.34 (t, *J* = 8.1 Hz, 6H), 7.20 (t, *J* = 7.6 Hz, 2H), 7.10 (d, *J* = 7.7 Hz, 2H), 7.05 (d, *J* = 7.7 Hz, 2H), 5.68 (d, *J* = 22.5 Hz, 1H), 4.53 (t, *J* = 4.9 Hz, 2H), 4.20-4.05 (m, 2H), 3.87 (t, *J* = 4.9 Hz, 2H), 3.80 (t, *J* = 7.5 Hz, 1H), 3.71-3.61 (m, 2H), 3.58 (d, *J* = 12.4 Hz, 8H), 3.50 (t, *J* = 5.5 Hz, 2H), 3.36-3.32 (m, 2H), 2.75 (t, *J* = 7.5 Hz, 2H), 2.18 (t, *J* = 6.9 Hz, 2H), 2.05-1.93 (m, 2H), 1.65-1.54 (m, 2H), 1.52-1.42 (m, 2H), 1.37-1.31 (m, 4H), 1.08 (d, *J* = 6.2 Hz, 3H). ^{13}C NMR (100 MHz, Methanol-*d₄*) δ (ppm) 176.3, 134.9, 131.2, 131.1, 131.0, 131.0, 126.9, 126.8, 126.5, 121.6, 121.6, 121.5, 121.5, 71.5, 71.4, 71.4, 71.2, 70.6, 70.4, 65.8, 57.4, 53.0, 52.7, 51.4, 40.3, 36.9, 30.9, 30.7, 30.2, 29.9, 27.1, 26.9, 22.7, 15.6. MS (ESI) *m/z* 921.5 $[M+H]^+$. HRMS for $C_{44}H_{61}N_{10}O_{10}P_1$, mass calculated 921.4382, mass measured 921.4416.

3-(1-(18-((4R,5S)-5-methyl-2-oxoimidazolidin-4-yl)-13-oxo-3,6,9-trioxa-12-azaoctadecyl)-1H-1,2,3-triazol-4-yl)propyl (1-(bis(4-acetamido-phenoxy)phosphoryl) (4-guanidinophenyl)carbamate trifluoroacetate salt (56b) General procedure ***H*** with **38a** (150 mg, 0.183 mmol) and **S16**, followed by general procedure ***D*** to yield **56b** (41.6 mg, 0.040 mmol, 22% yield). ^{1}H NMR (400 MHz, Methanol-*d₄*) δ (ppm) 7.81 (br s, 1H), 7.68 (d, *J* = 6.9 Hz, 2H), 7.51 (dd, *J* = 8.9 , 5.0 Hz, 4H), 7.34 (d, *J* = 8.3 Hz, 2H), 7.05 (d, *J* = 8.4 Hz, 2H), 6.99 (d, *J* = 8.4 Hz, 2H), 5.65 (d, *J* = 22.8 Hz, 1H), 4.53 (t, *J* = 4.9 Hz, 2H), 4.19-4.02 (m, 2H), 3.87 (t, *J* = 4.9 Hz, 2H), 3.84-3.77 (m, 1H), 3.71-3.65 (m, 2H), 3.57 (d, *J* = 11.4 Hz, 8H), 3.49 (t, *J* = 5.9, 2H), 3.35-3.31 (m, 2H), 2.77 (br s, 2H), 2.18 (t, *J* = 7.1 Hz, 2H), 2.10 (s, 6H), 2.04-1.96 (m, 2H), 1.64-1.54 (m, 2H), 1.51-1.42 (m, 2H), 1.37-1.30 (m, 4H), 1.08 (d, *J* = 6.4 Hz, 3H). ^{13}C NMR (100 MHz, Methanol-*d₄*) δ (ppm) 170.2, 129.8, 129.7, 125.1, 120.9, 120.9, 120.5, 120.4, 120.4, 120.3, 70.1, 70.1, 70.0, 69.8, 69.2, 69.0, 56.0, 51.3, 39.0, 35.5, 29.3, 29.0, 25.8, 25.4, 22.3, 14.2. MS (ESI) *m/z* 1057.6 $[M+Na]^+$.

3-(1-(18-((4R,5S)-5-methyl-2-oxoimidazolidin-4-yl)-13-oxo-3,6,9-trioxa-12-azaoctadecyl)-1H-1,2,3-triazol-4-yl)propyl (1-(bis(4-chlorophenoxy)phosphoryl) (4-guanidinophenyl)carbamate trifluoroacetate salt (57b) General procedure ***H*** with **39a** (105 mg, 0.252 mmol) and **S16**, followed by general procedure to yield **57b** (50.4 mg, 0.050 mmol, 20% yield). ^{1}H NMR (400 MHz, Methanol-*d₄*) δ (ppm) 7.78 (s, 1H), 7.69 (d, *J* = 8.0 Hz, 2H), 7.35-7.30 (m, 6H), 7.12 (d, *J* = 9.1 Hz, 2H), 7.05 (d, *J* = 9.1 Hz, 2H), 5.66 (d, *J* = 23.2 Hz, 1H), 4.50 (t, *J* = 4.7 Hz, 2H), 4.16-4.01 (m, 2H), 3.83 (t, *J* = 4.8 Hz, 2H), 3.84-3.77 (m, 1H), 3.71-3.62 (m, 3H), 3.59 (s, 4H), 3.54 (s, 4H), 3.47 (t, *J* = 6.1 Hz, 2H), 3.30 (d, *J* = 5.8 Hz, 2H), 2.75 (t, *J* = 8.2 Hz, 2H), 2.15 (t, *J* = 6.7 Hz, 2H), 2.04-1.93 (m, 2H), 1.65-1.41 (m, 6H), 1.04 (d, *J* = 6.2 Hz, 3H). ^{13}C NMR (100 MHz, Methanol-*d₄*) δ (ppm) 176.2, 136.8, 136.8, 134.5, 132.3, 132.2, 131.2, 131.1, 131.0, 131.0, 126.5, 123.2, 123.2, 123.1, 123.0, 71.5, 71.5, 71.4, 71.2, 70.6, 70.4, 65.8, 57.4, 53.0, 52.7, 51.3, 40.3, 36.9, 30.9, 30.7, 30.2, 29.9, 27.5, 27.1, 26.8, 22.7, 15.6. MS (ESI) *m/z* 989.4 $[M+H]^+$. HRMS for $C_{44}H_{59}N_{10}O_{10}Cl_2P_1$, mass calculated 989.3603, mass measured 989.3636.

Diphenyl (5-amino-1-(1-(4-(1-(18-((4S,5R)-5-methyl-2-oxoimidazolidin-4-yl)-13-oxo-3,6,9-trioxa-12-azaoctadecyl)-1H-1,2,3-triazol-4-yl)butanoyl)pyrrolidine-2-carboxamido)pentyl)phosphonate trifluoroacetate salt (58b) General procedure ***H*** with **31a** (52,5 mg, 0,127 mmol) and **S16**, followed by general procedure ***D*** to yield **58b** (5.6 mg, 5.31 µmol, 6%

yield). ^{1}H NMR (400 MHz, Methanol-d_4) δ (ppm) 7.86-7.75 (m, 2H), 7.36 (t, J = 7.7 Hz, 4H), 7.27-7.19 (m, 2H), 7.19-7.11 (m, 4H), 4.54 (t, J = 5.1 Hz, 2H), 4.48 (dd, J = 7.7, 4.6 Hz, 1H), 4.45-4.35 (m, 1H), 4.29 (dd, J = 7.7, 4.6 Hz, 1H), 4.19-4.04 (m, 2H), 3.88 (t, J = 5.1 Hz, 2H), 3.63-3.55 (m, 8H), 3.51 (t, J = 5.4 Hz, 2H), 3.34 (t, J = 5.7 Hz, 4H), 3.23-3.15 (m, 1H), 3.02-2.87 (m, 4H), 2.79 (t, J = 7.4 Hz, 2H), 2.72-2.65 (m, 1H), 2.19 (t, J = 7.4 Hz, 2H), 2.15-1.34 (m, 19H). ^{13}C NMR (100 MHz, Methanol-d_4) δ (ppm) 131.1, 121.5, 71.5, 70.6, 52.7, 40.4, 36.9, 30.8, 30.2, 26.9, 15.7. MS m/z 940.6 $[M+H]^+$. HRMS for $C_{46}H_{70}N_9O_{10}P_1$, mass calculated 940.5056, mass measured 940.5059.

2.2 Biochemistry evaluation

2.2.1 Additional IC_{50} values

Supplementary Table 1. IC_{50} values of biotin probes against a panel of serine proteases.

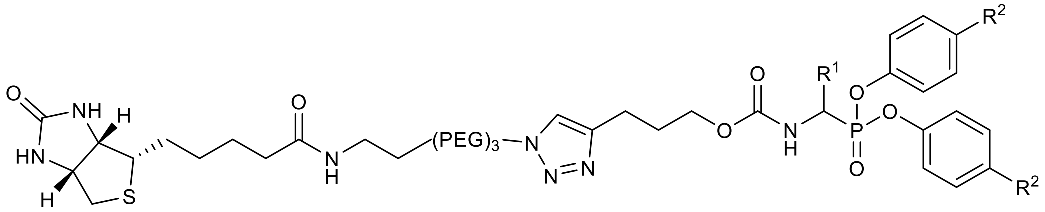

#	R1	R2	Trypsin-3	β-tryptase	Thrombin	uPA[a]	CatG[a]	ChTryp[a]	NE[a]
			IC50 (μM)[b]						
**43**	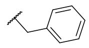	H	>10	>10	>10	>10	2.77 ± 0.07	2.64 ± 0.27	>10
**48**	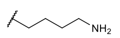	H	0.21 ± 0.005	2.11 ± 0.10	2.73 ± 0.17	3.54 ± 0.23	>10	>10	>10
**51**	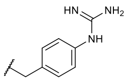	H	0.02 ± 0.003	0.91 ± 0.09	6.04 ± 0.40	0.04 ± 0.001	0.17 ± 0.01	>10	>10
**53**		$NHCOCH_3$	0.01 ± 0.001	0.08 ± 0.003	0.49 ± 0.02	0.01 ± 0.002	0.05 ± 0.001	>10	>10

[a] Panel of serine proteases abbreviations: urokinase plasminogen activator (uPA), cathepsin G (catG), chymotrypsin (ChTryp), neutrophil elastase (NE).
[b] Half maximal inhibitory concentration (IC_{50}) value is the concentration of inhibitor required to reduce the enzyme activity to 50% after a 15 min preincubation with the enzyme at 37 °C. IC_{50} are calculated from two independent experiments; when SD was higher than three times the average value, a third independent experiment was run (mean ± SD).

2.2.2 Jump dilution results

Supplementary Table 2. Jump dilution of selected alkyne and desthiobiotin probes against a panel of trypsin-like serine proteases.

#	R^1	R^2	Alk / DtB[a]	Trypsin-3	β-tryptase	Thrombin	uPA[b]	CatG[b]
**32b**	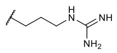	H	Alk	Irrev.	Irrev.	Rev.	Irrev.	Rev.
**45b**		H	DtB	Irrev.	Irrev.	Rev.	N.D.	N.D.
**33b**		Cl	Alk	Irrev.	Irrev.	Rev.	Irrev.	Rev.
**46b**		Cl	DtB	Irrev.	Irrev.	Rev.	N.D.	N.D.
**34b**	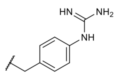	H	Alk	Rev.	Rev.	Rev.	Irrev.	Rev.
**50b**		H	DtB	Rev.	Rev.	Rev.	Irrev.	Rev.
**35b**		NHCOMe	Alk	Rev.	Rev.	Rev.	Irrev.	Rev.
**52b**		NHCOMe	DtB	Rev.	Rev.	Rev.	Irrev.	Rev.
**36b**		Cl	Alk	Rev.	Rev.	Rev.	Irrev.	Rev.
**54b**		Cl	DtB	Rev.	Rev.	Rev.	Irrev.	Rev.
**37b**	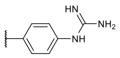	H	Alk	Irrev.	Irrev.	N.D.	Irrev.	Irrev.
**55b**		H	DtB	Irrev.	Irrev.	N.D.	Irrev.	Irrev.
**38b**		NHCOMe	Alk	Irrev.	Irrev.	N.D.	Irrev.	Irrev.
**56b**		NHCOMe	DtB	Irrev.	Irrev.	N.D.	Irrev.	Irrev.
**39b**		Cl	Alk	Irrev.	Irrev.	N.D.	Irrev.	Rev.
**57b**		Cl	DtB	Irrev.	Irrev.	N.D.	Irrev.	Rev.
**41b**	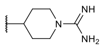	H	Alk	Irrev.	Irrev.	Irrev.	N.D.	N.D.
**31b**	Pro-Lys	H	Alk	Irrev.	Irrev.	Irrev.	Irrev.	N.D.

[a] Alk: ABP precursor bearing an alkyne; DtB: desthiobiotin probe
[b] Panel of serine proteases abbreviations: urokinase plasminogen activator (uPA), cathepsin G (catG)
N.D.: IC_{50} is greater than 10μM, and jump dilution was not performed

2.2.3 Progress curves and Jump dilution experiments

Kinetic assays were performed in the following manner. The inhibitor was mixed with the substrate, and the buffer solution with the enzyme was added at the time zero. The progress curves show the release of *para*-nitroaniline (pNA) or 7-amino-4-methyl coumarin (AMC) as a function of time.

To monitor the dissociation of the inhibitor-enzyme, aliquots of the enzyme were incubated at $37^{o}C$ without and with the inhibitor at a concentration 10 times higher than its IC_{50}. The enzyme concentration was 2.5 times higher than the concentration used for the IC_{50} determinations. After 15 min, the aliquots were diluted 10-fold or 100-fold into the substrate concentration and assay buffer used for the IC_{50} determination. The dissociation of the enzyme-inhibitor complex was monitored by substrate hydrolysis over time. Blue lines represent the control where no inhibitor was present. Pink lines represent the assay with inhibitor.

Compound 32b

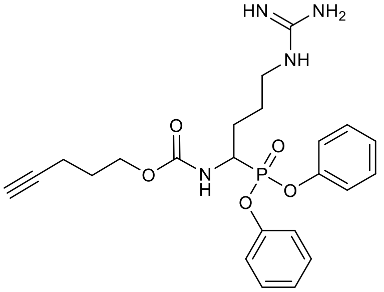

	Progress curve	k_{app} calculation	Jump dilution
**Trypsin-3** **IRREV.**	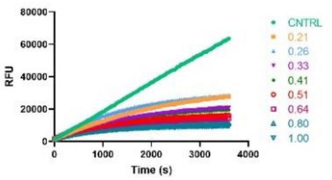	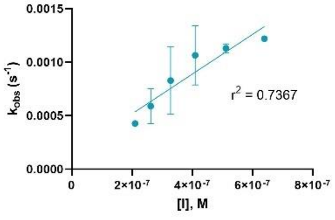	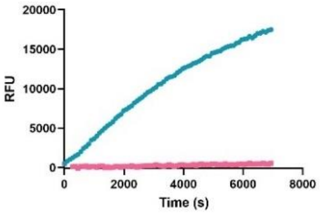
**β-tryptase** **IRREV.**	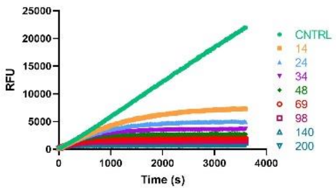	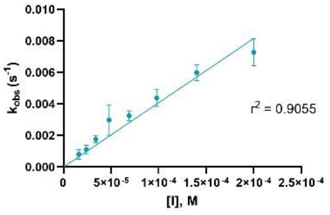	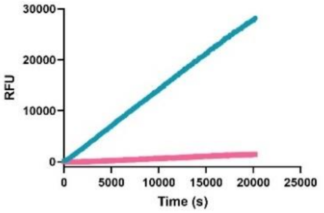
**Thrombin** **REV.**	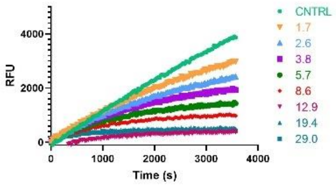	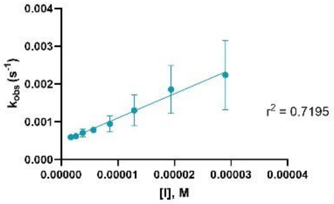	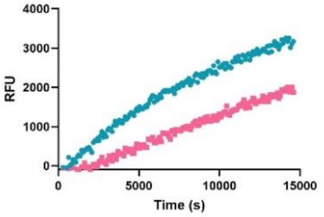
**uPA** **IRREV.**	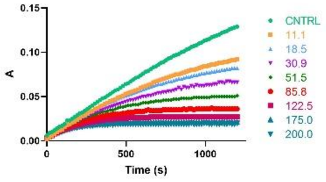	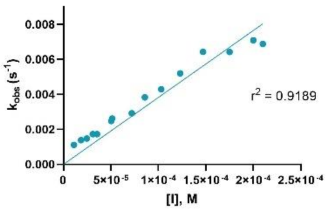	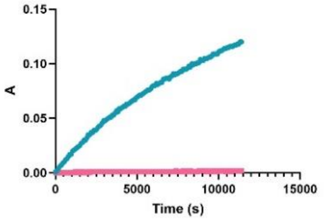
**CatG** **REV.**	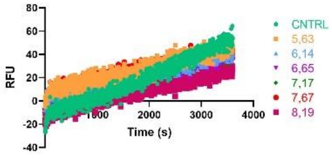	N.D.	v_i/v_0 original mixture – 0,37 v_i/v_0 diuted mixture – 0,91 Activity increases when diluted

<u>**Compound 45b**</u>

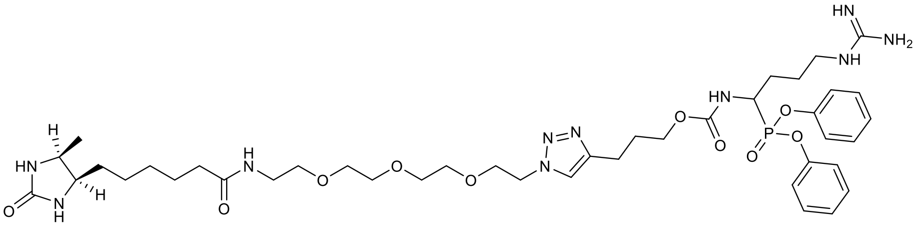

	Progress curve	k_{app} calculation	Jump dilution
**Trypsin-3**<br>**IRREV.**	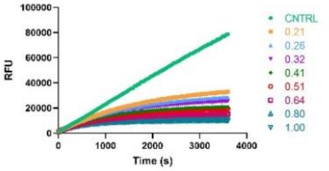	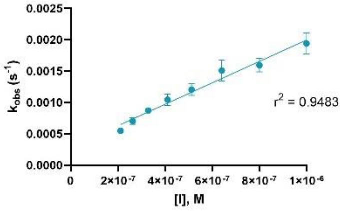	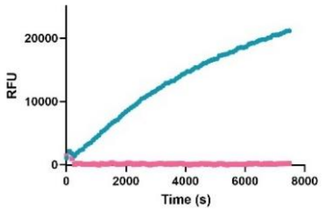
**β-tryptase**<br>**IRREV.**	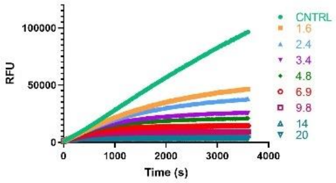	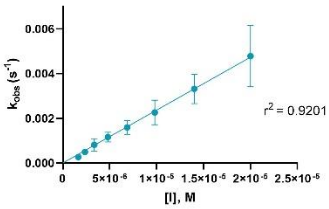	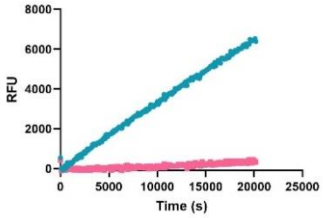
**Thrombin**<br>**REV.**	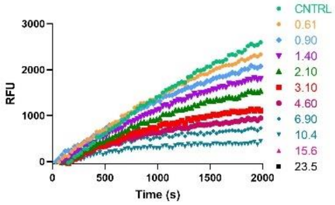	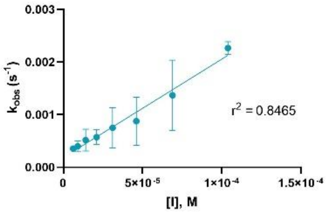	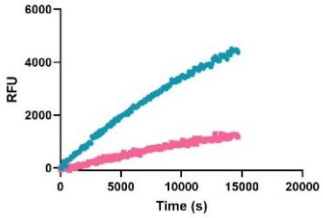

Compound 33b

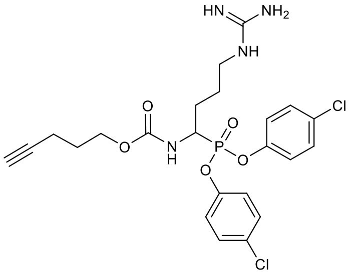

	Progress curve	k_{app} calculation	Jump dilution
**Trypsin-3**<br>**IRREV.**	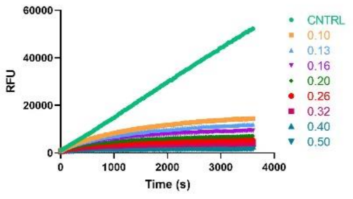	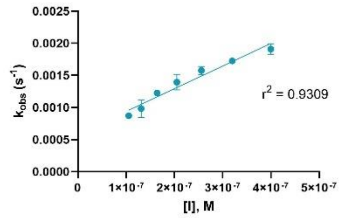	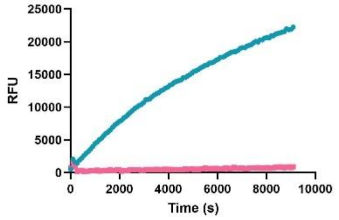
**β-tryptase**<br>**IRREV.**	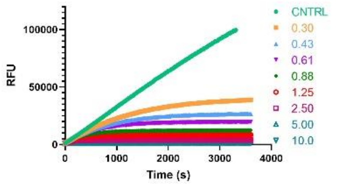	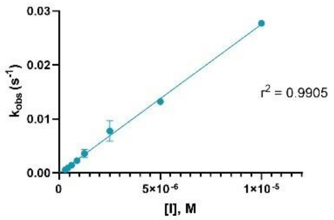	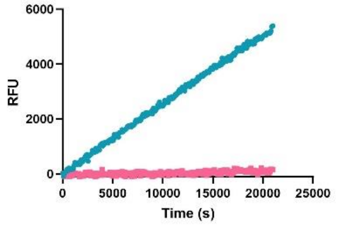
**Thrombin**<br>**REV.**	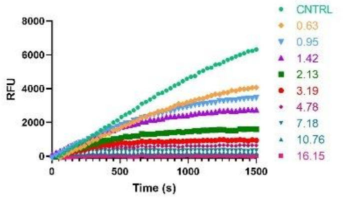	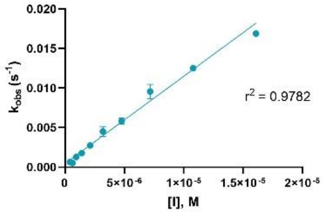	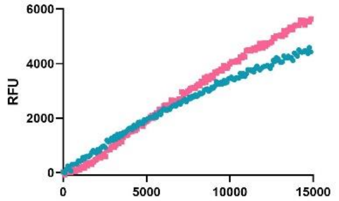
**uPA**<br>**IRREV.**	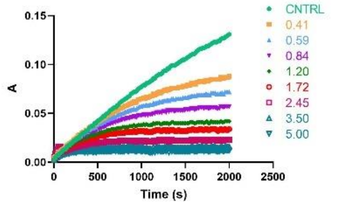	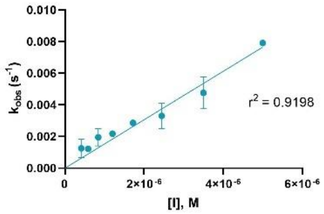	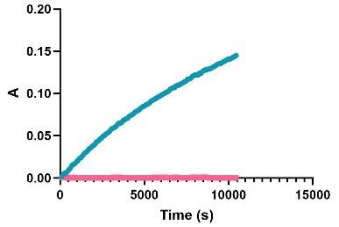
**CatG**<br>**REV.**	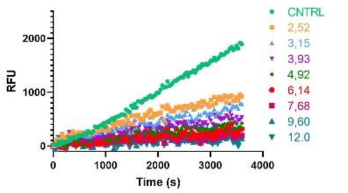	N.D.	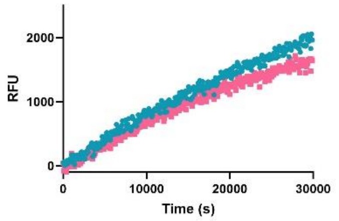

Compound 46b

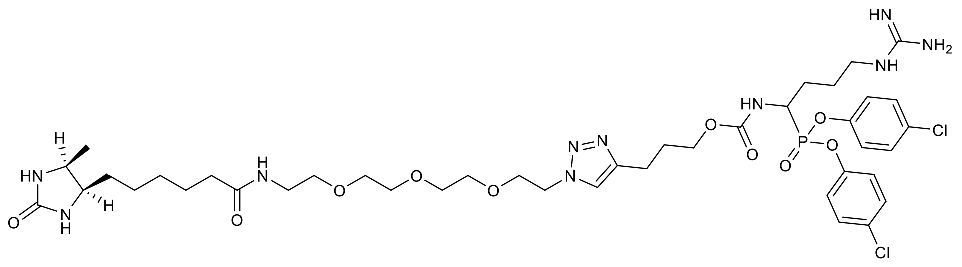

	Progress curve	k_{app} calculation	Jump dilution
Trypsin-3<br>IRREV.	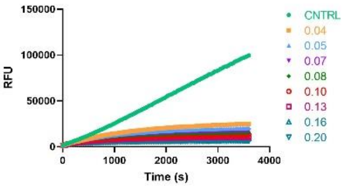	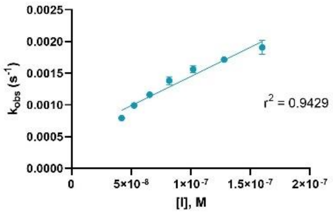	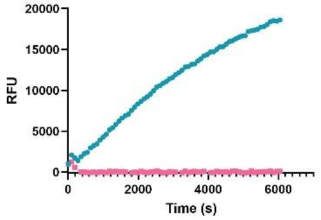
β-tryptase<br>IRREV.	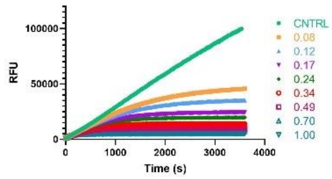	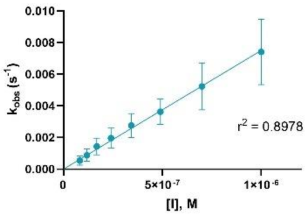	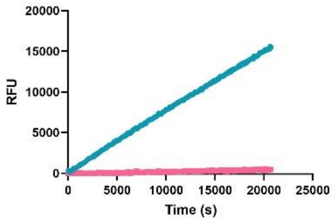
Thrombin<br>REV.	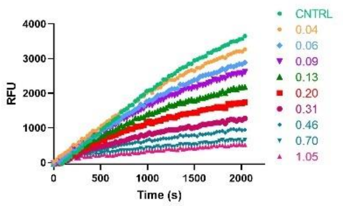	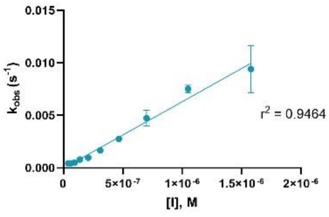	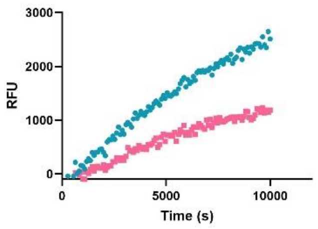
uPA<br>IRREV.	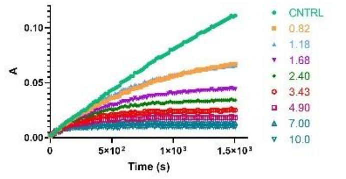	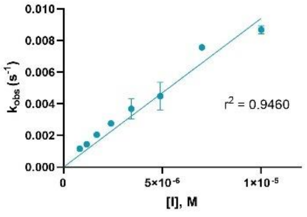	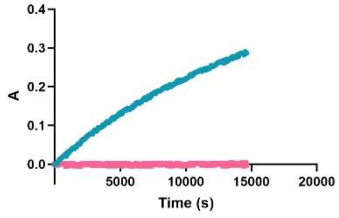

Compound 34b

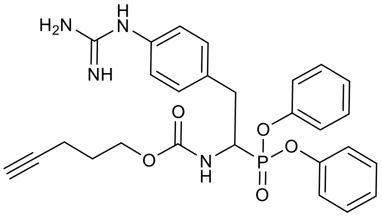

<table>
<tr><th></th><th>Progress curve</th><th>k_{app} calculation</th><th>Jump dilution</th></tr>
<tr><td>Trypsin-3

REV.</td><td>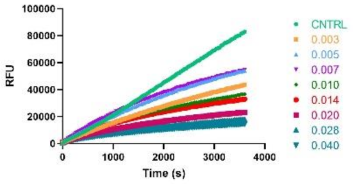</td><td>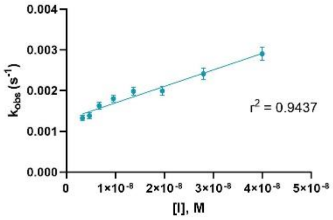</td><td>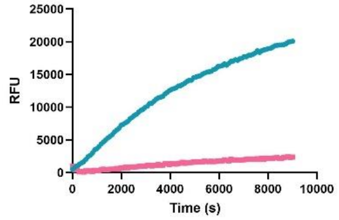</td></tr>
<tr><td>β-tryptase

REV.</td><td>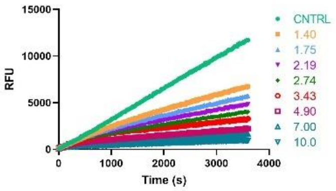</td><td>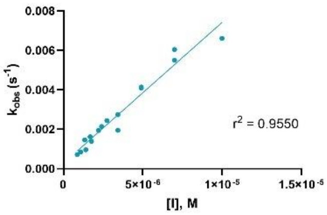</td><td>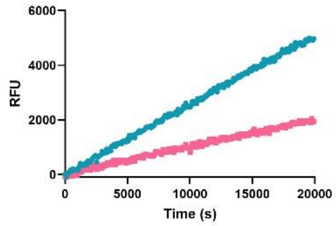</td></tr>
<tr><td>Thrombin

REV.</td><td>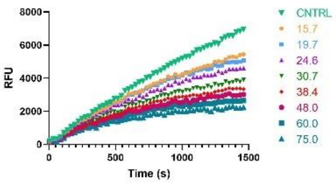</td><td>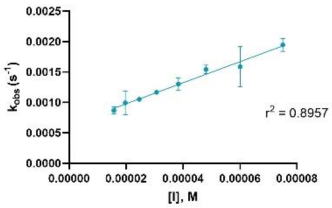</td><td>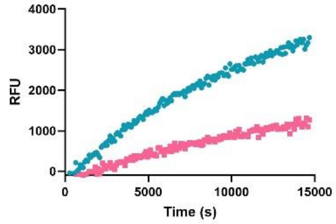</td></tr>
<tr><td>uPA

IRREV.</td><td>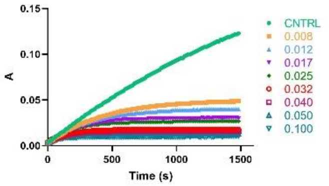</td><td>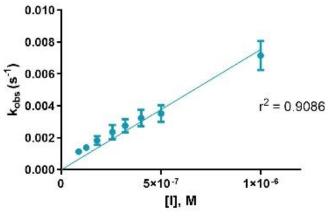</td><td>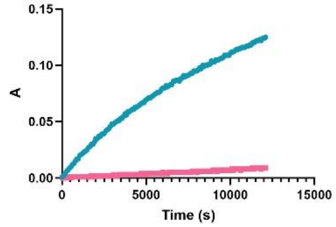</td></tr>
<tr><td>CatG

REV.</td><td>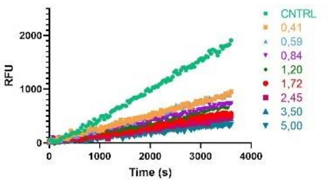</td><td>N.D.</td><td>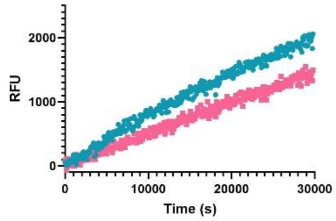</td></tr>
</table>

Compound 50b

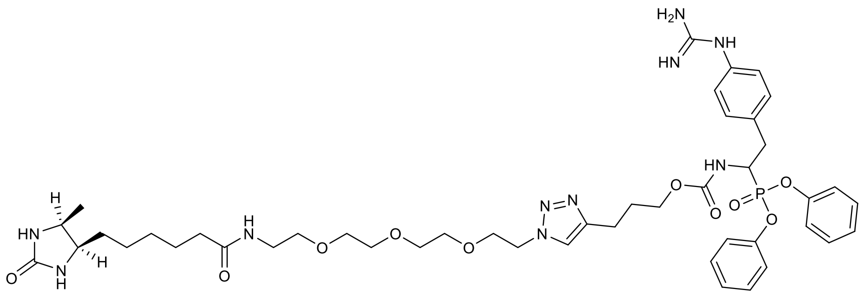

	Progress curve	k_{app} calculation	Jump dilution
**Trypsin-3**<br><br>**REV.**	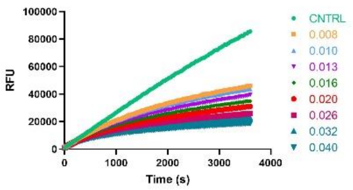	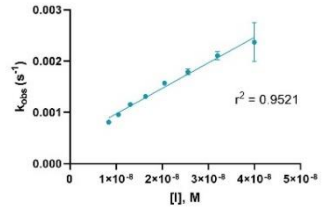	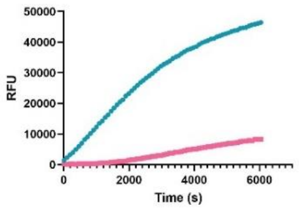
**β-tryptase**<br><br>**REV.**	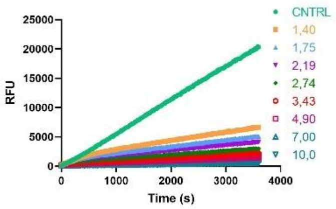	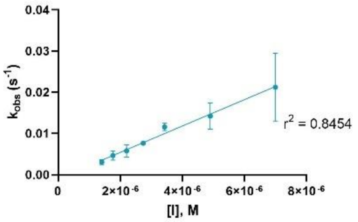	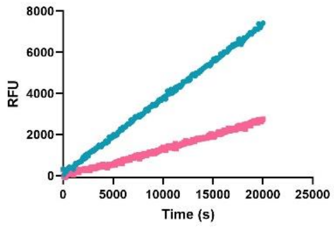
**Thrombin**<br><br>**REV.**	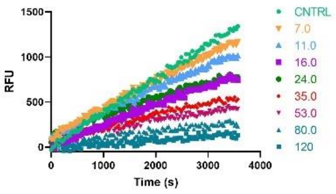	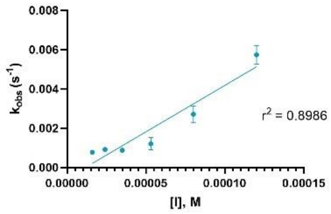	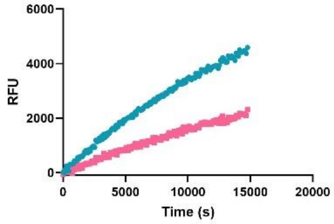
**uPA**<br><br>**IRREV.**	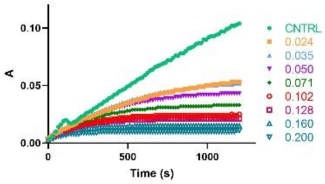	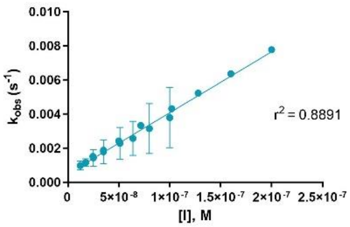	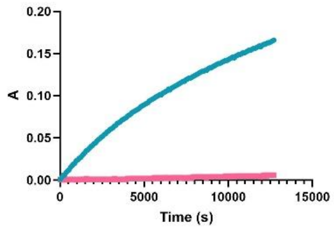
**CatG**<br><br>**REV.**	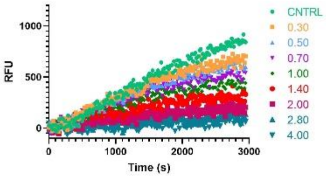	N.D.	v_i/v_0 original mixture – 0,19<br>v_i/v_0 diuted mixture – 0,39<br><br>Activity increases when diluted

Compound 35b

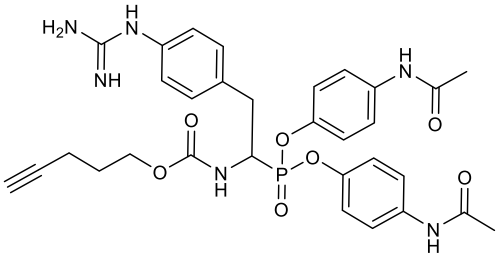

	Progress curve	k_{app} calculation	Jump dilution
**Trypsin-3** **REV.**	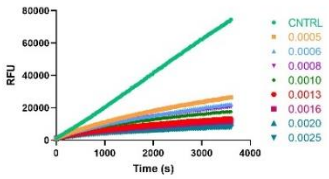	N.D.	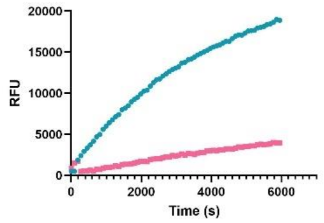
**β-tryptase** **REV.**	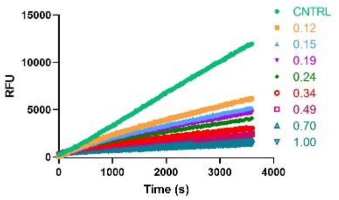	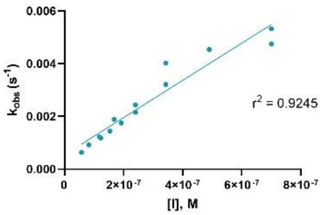	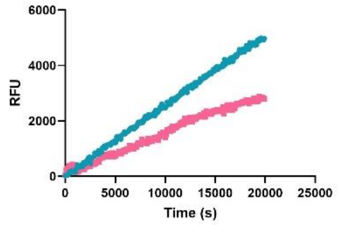
**Thrombin** **REV.**	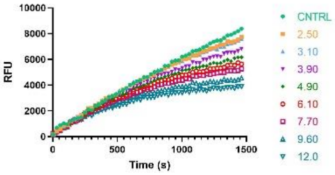	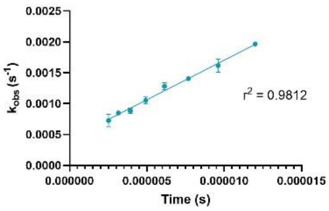	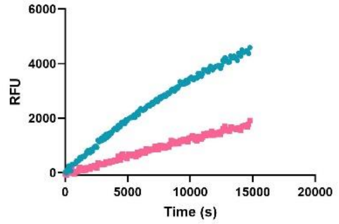
**uPA** **IRREV.**	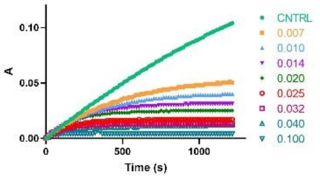	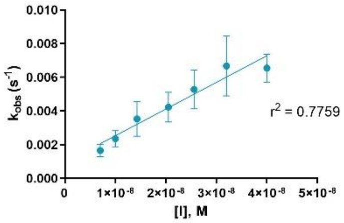	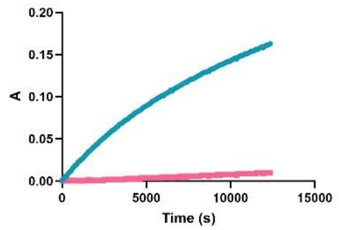
**CatG** **REV.**	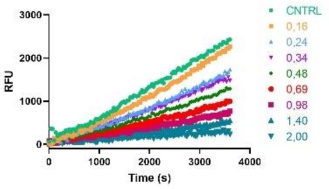	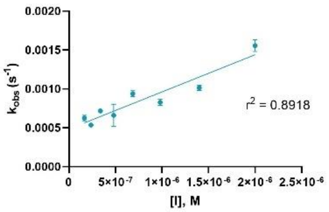	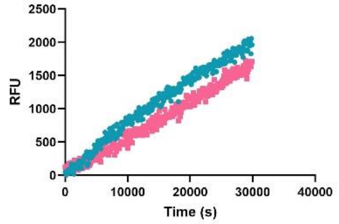

Compound 52b

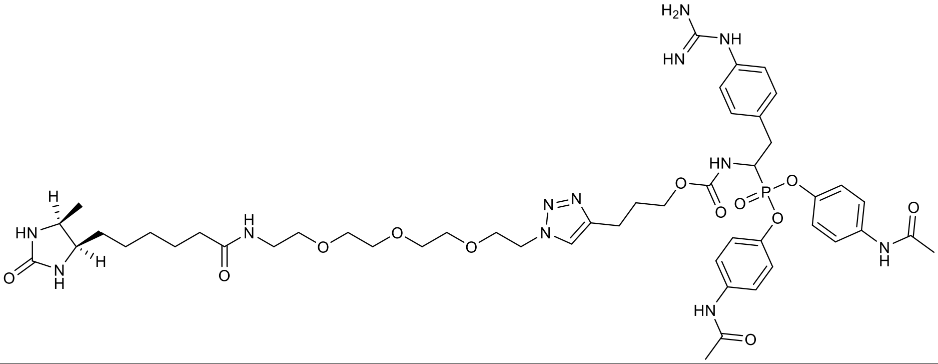

	Progress curve	k_{app} calculation	Jump dilution
**Trypsin-3**<br><br>**REV.**	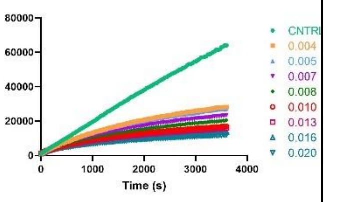	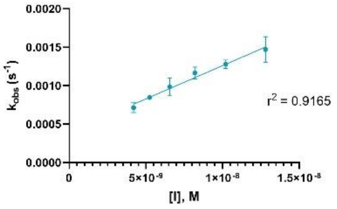	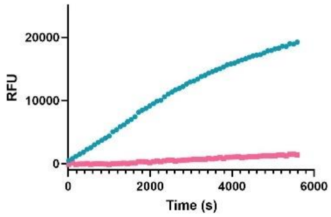
**β-tryptase**<br><br>**REV.**	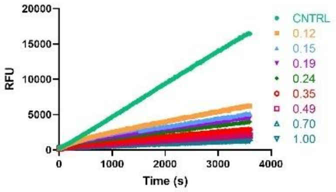	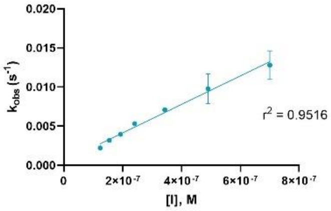	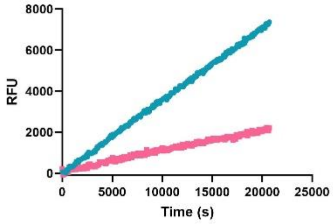
**Thrombin**<br><br>**REV.**	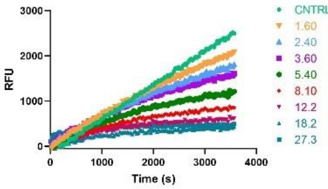	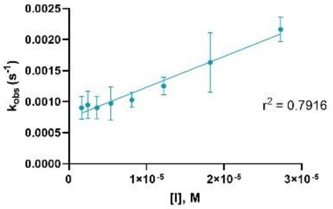	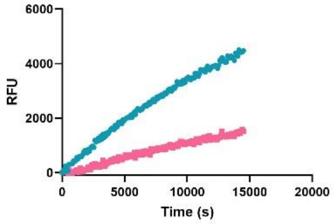
**uPA**<br><br>**IRREV.**	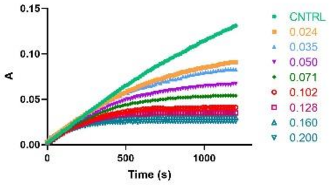	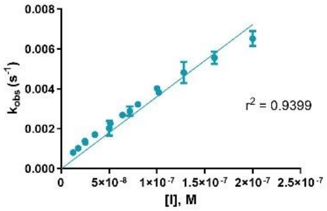	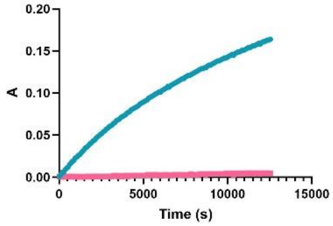
**CatG**<br><br>**REV.**	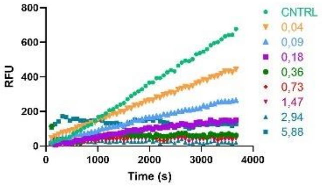	N.D.	v_i/v_0 original mixture – 0,11<br>v_i/v_0 diuted mixture – 0,31<br><br>Activity increases when diluted

Compound 36b

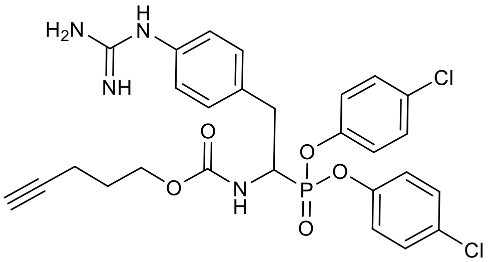

	Progress curve	k_{app} calculation	Jump dilution
**Trypsin-3**<br><br>**REV.**	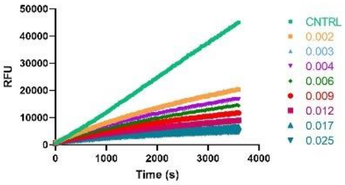	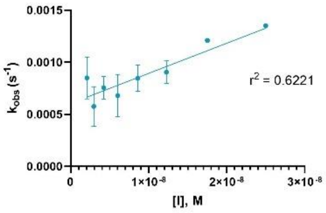	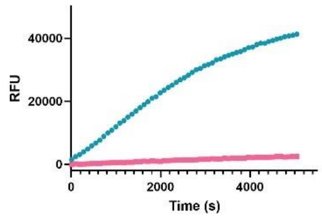
**β-tryptase**<br><br>**REV.**	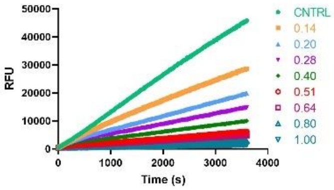	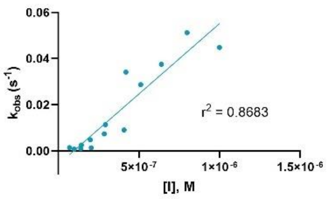	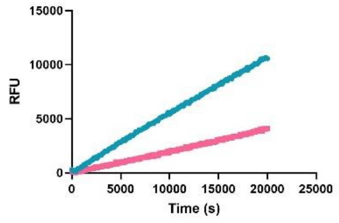
**Thrombin**<br><br>**REV.**	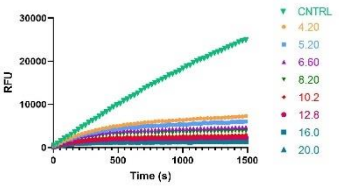	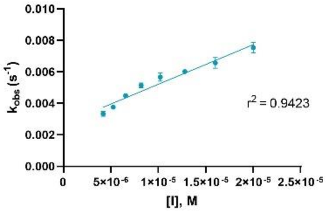	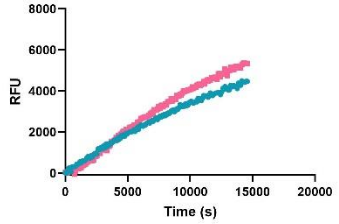
**uPA**<br><br>**IRREV.**	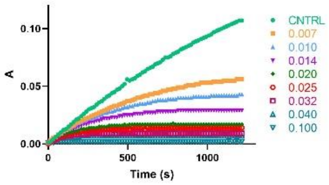	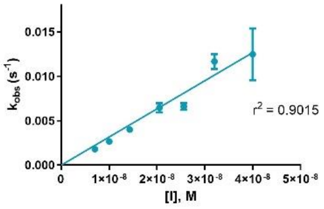	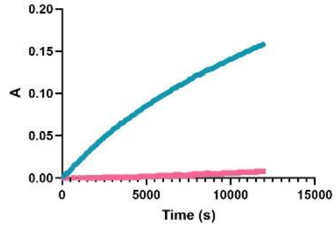
**CatG**<br><br>**REV.**	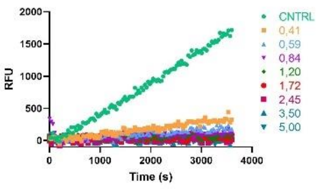	N.D.	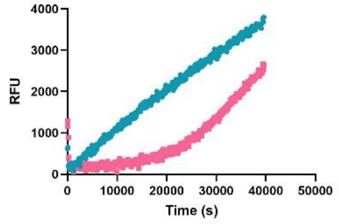

Compound 54b

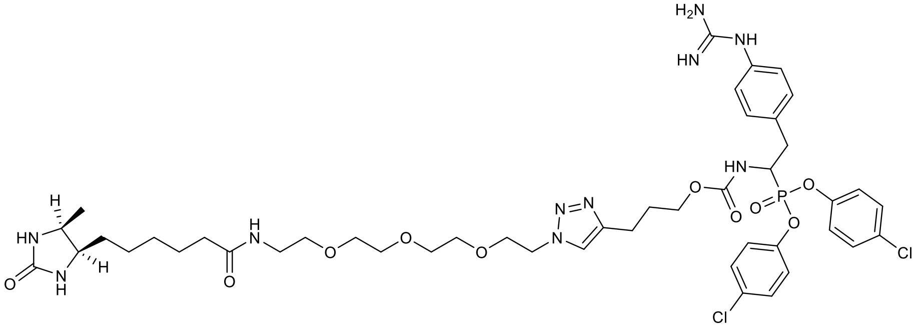

	Progress curve	k_{app} calculation	Jump dilution
**Trypsin-3**<br><br>**REV.**	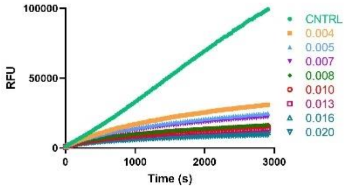	N.D.	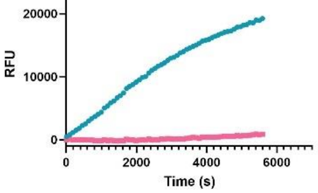
**β-tryptase**<br><br>**REV.**	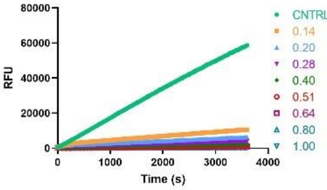	N.D.	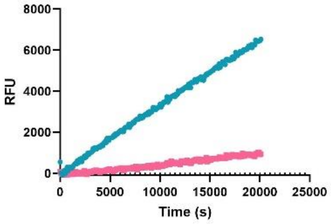
**Thrombin**<br><br>**REV.**	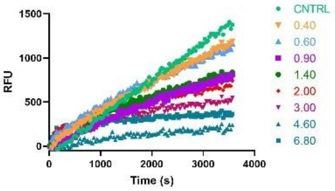	N.D.	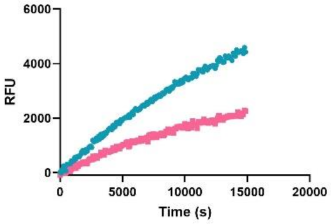
**uPA**<br><br>**IRREV.**	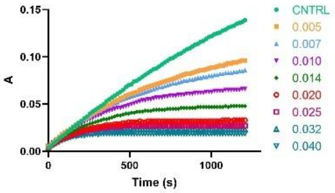	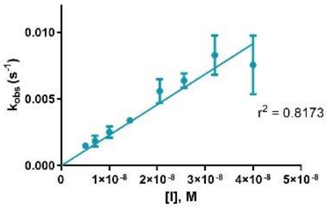	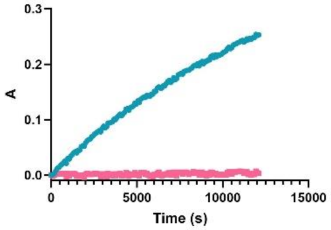
**CatG**<br><br>**REV.**	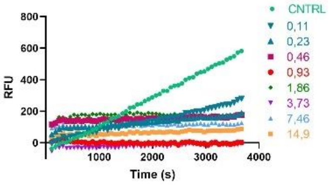	N.D.	v_i/v_0 original mixture – 0,13<br>v_i/v_0 diuted mixture – 0,38<br><br>Activity increases when diluted

Compound 37b

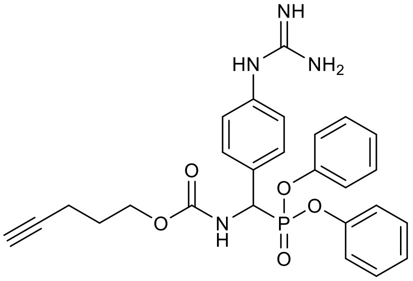

	Progress curve	k_{app} calculation	Jump dilution
**Trypsin-3**<br><br>**IRREV.**	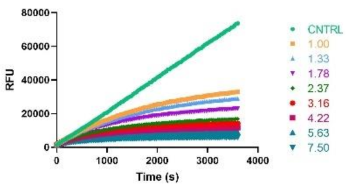	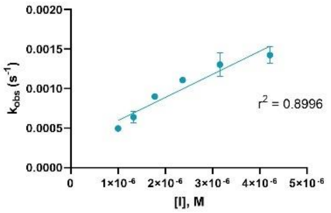	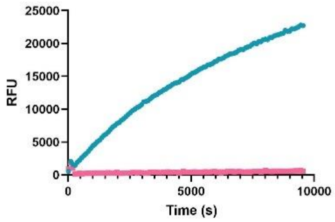
**β-tryptase**<br><br>**IRREV.**	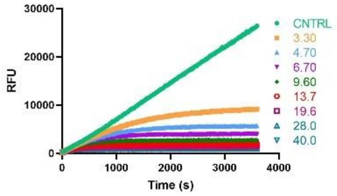	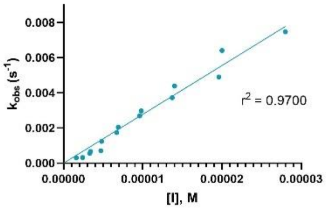	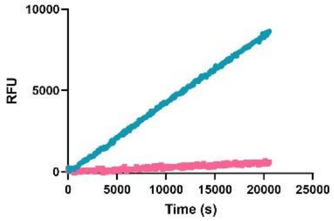
**uPA**<br><br>**IRREV.**	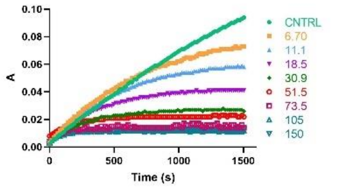	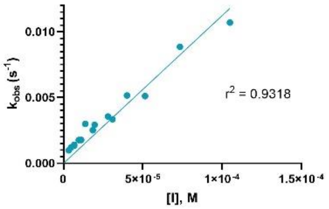	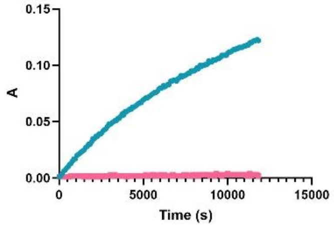
**CatG**<br><br>**IRREV.**	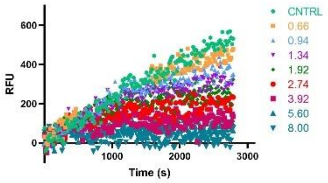	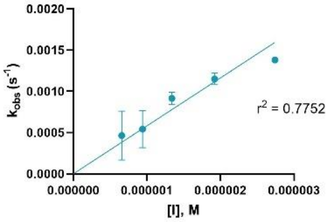	v_i/v_0 original mixture – 0,16<br>v_i/v_0 diuted mixture – 0,20<br><br>Activity does not increases when diluted

Compound 55b

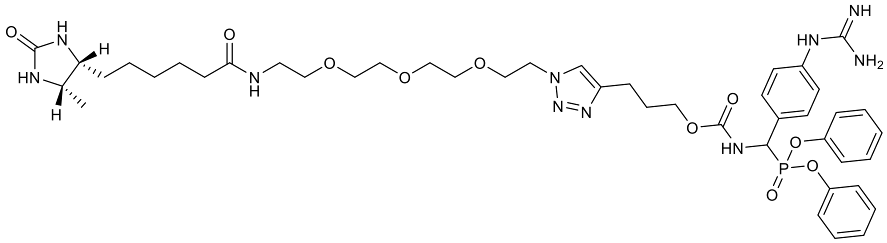

	Progress curve	k_{app} calculation	Jump dilution
**Trypsin-3**<br><br>**IRREV.**	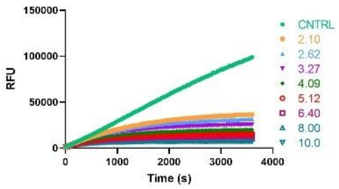	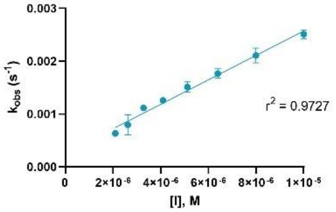	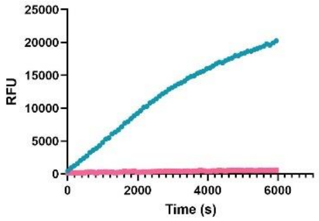
**β-tryptase**<br><br>**IRREV.**	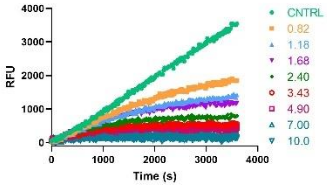	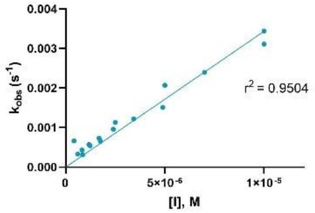	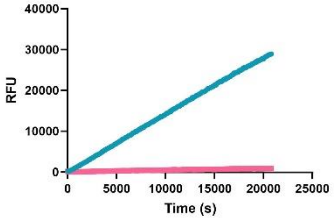
**uPA**<br><br>**IRREV.**	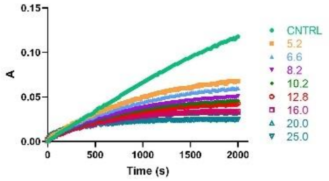	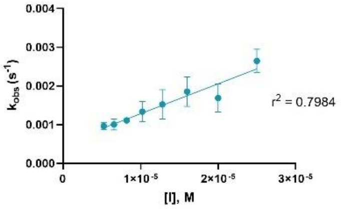	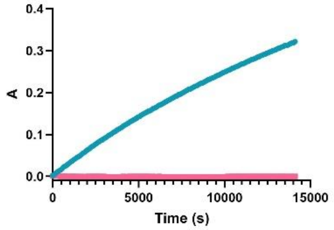
**CatG**<br><br>**IRREV.**	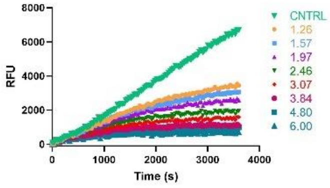	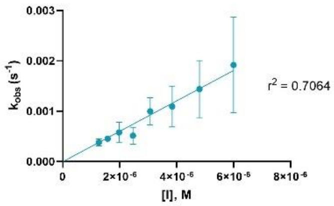	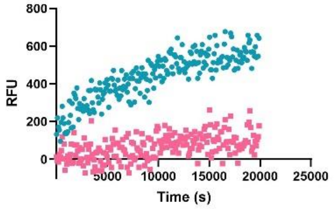

Compound 38b

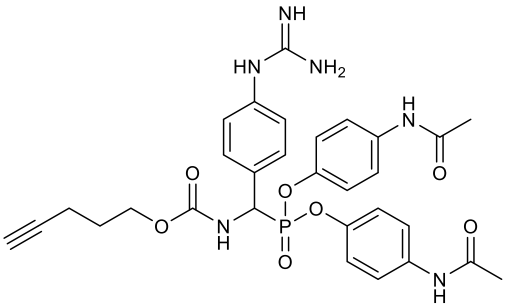

<table>
<tr><th></th><th>Progress curve</th><th>k_{app} calculation</th><th>Jump dilution</th></tr>
<tr><td>Trypsin-3

IRREV.</td><td>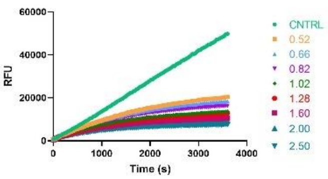
</td><td>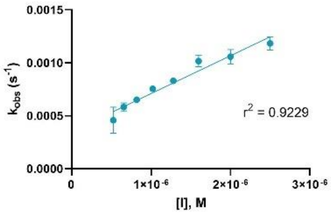
</td><td>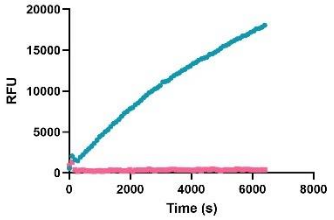
</td></tr>
<tr><td>β-tryptase

IRREV.</td><td>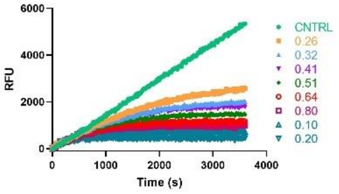
</td><td>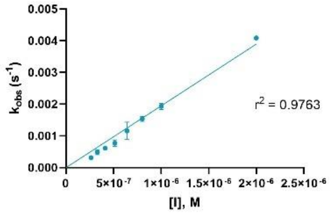
</td><td>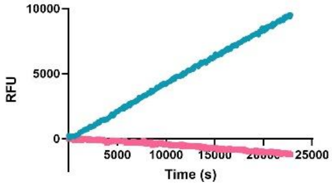
</td></tr>
<tr><td>uPA

IRREV.</td><td>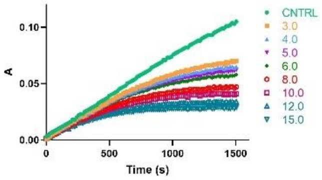
</td><td>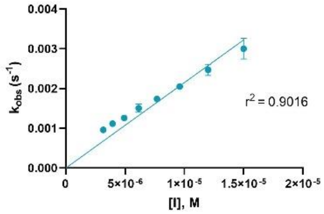
</td><td>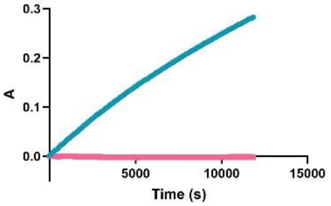
</td></tr>
<tr><td>CatG

IRREV.</td><td>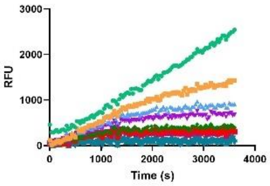
</td><td>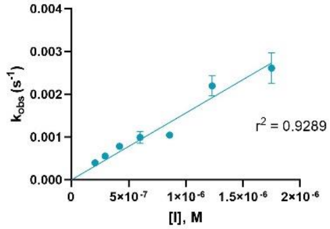
</td><td>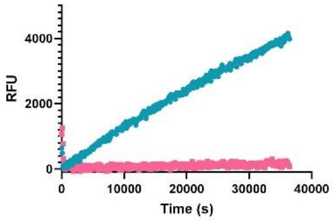
</td></tr>
</table>

Compound 56b

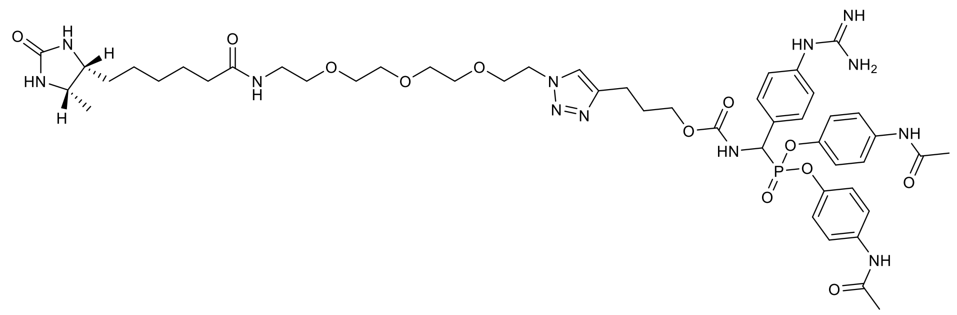

	Progress curve	k_{app} calculation	Jump dilution
**Trypsin-3**<br><br>**IRREV.**	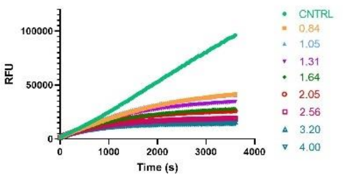	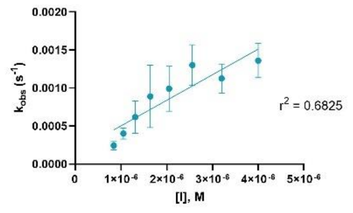	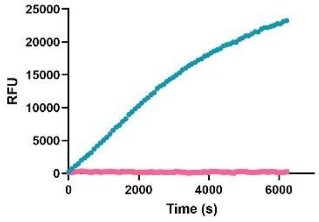
**β-tryptase**<br><br>**IRREV.**	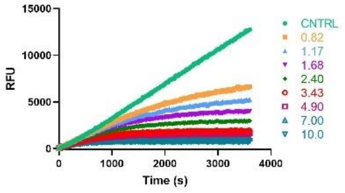	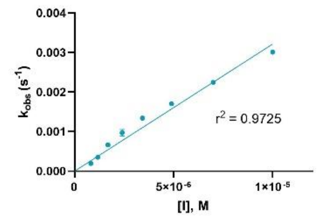	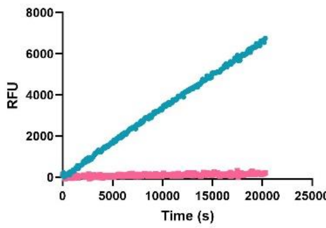
**uPA**<br><br>**IRREV.**	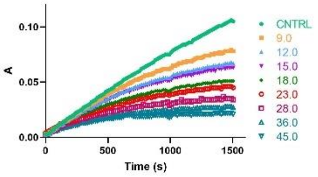	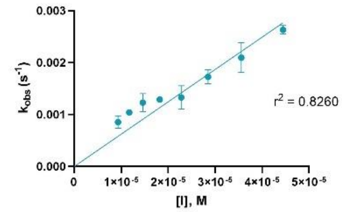	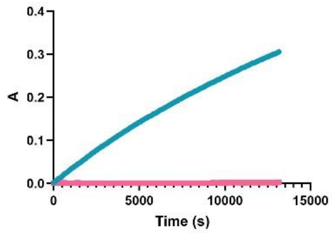
**CatG**<br><br>**IRREV.**	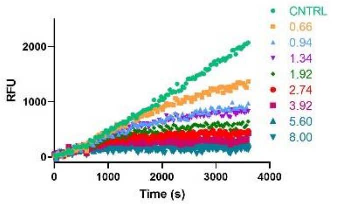	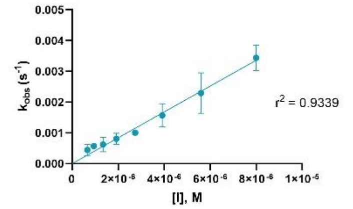	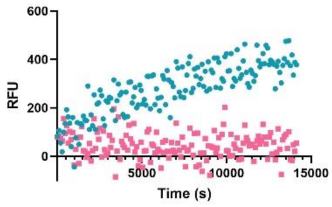

Compound 39b

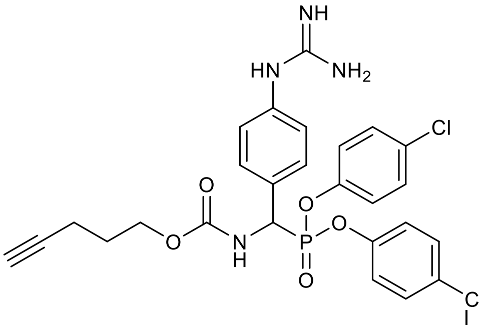

	Progress curve	kapp calculation	Jump dilution
**Trypsin-3**<br><br>**IRREV.**	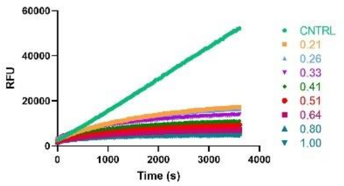	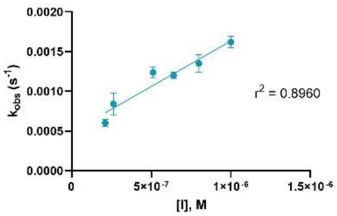	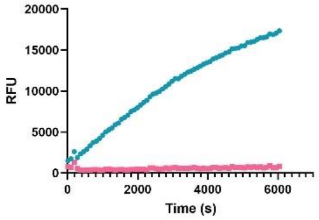
**β-tryptase**<br><br>**IRREV.**	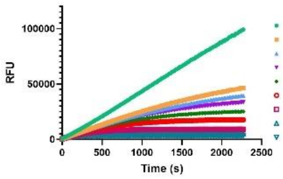	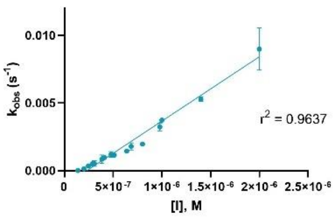	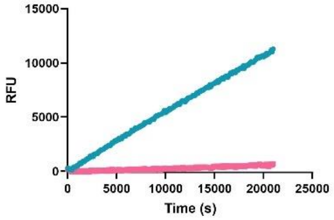
**uPA**<br><br>**IRREV.**	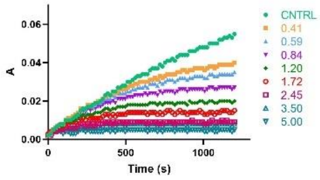	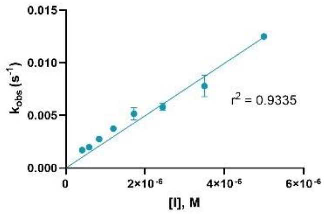	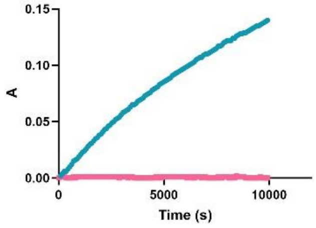
**CatG**<br><br>**REV.**	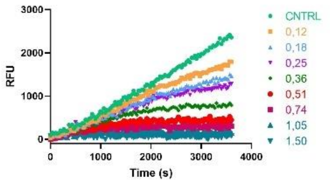	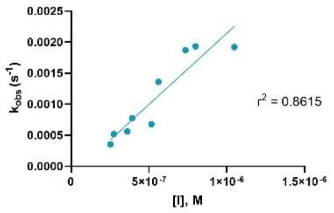	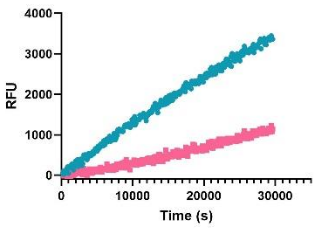

Compound 57b

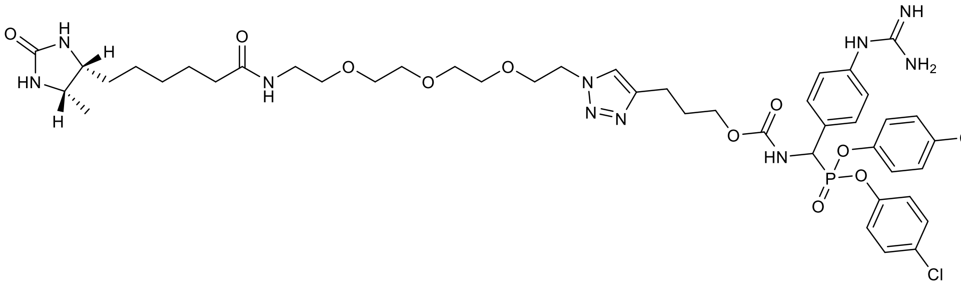

	Progress curve	k_{app} calculation	Jump dilution
**Trypsin-3**<br><br>**IRREV.**	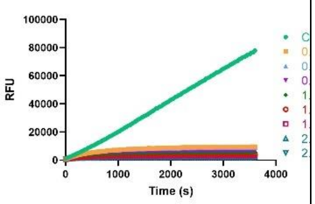	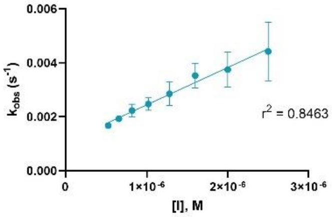	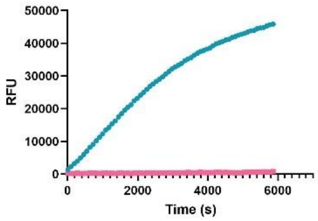
**β-tryptase**<br><br>**IRREV.**	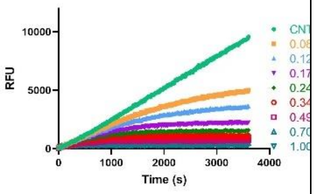	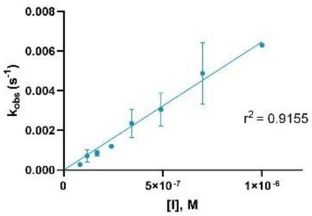	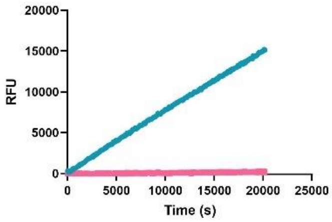
**uPA**<br><br>**IRREV.**	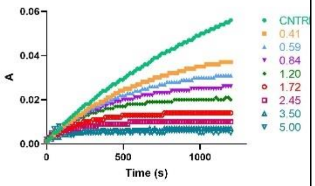	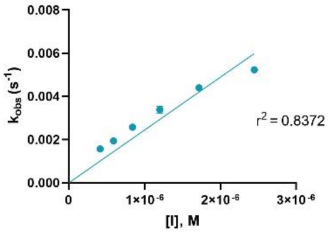	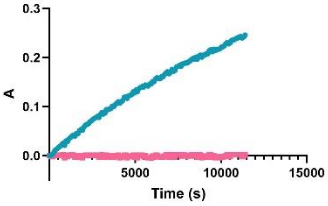
**CatG**<br><br>**REV.**	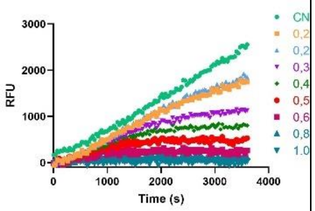	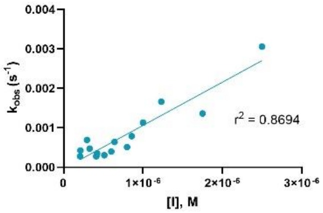	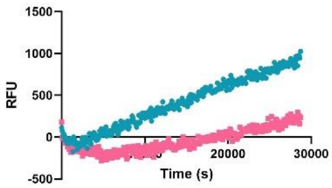

Compound 41b

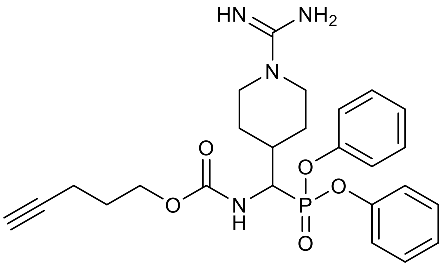

	Progress curve	k_{app} calculation	Jump dilution
**Trypsin-3**<br><br>**IRREV.**	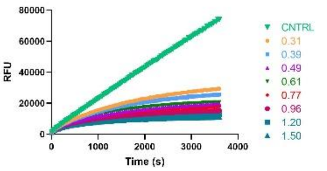	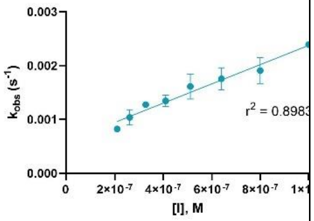	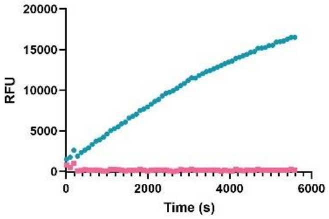
**β-tryptase**<br><br>**IRREV.**	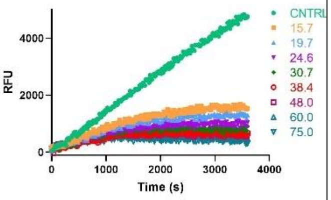	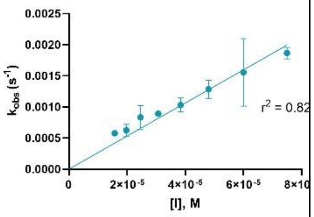	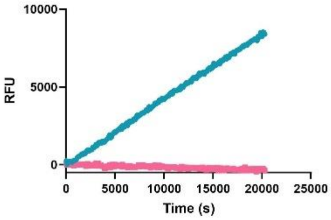
**Thrombin**<br><br>**IRREV.**	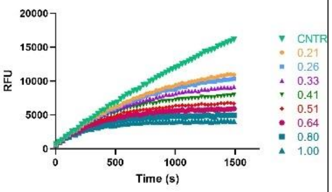	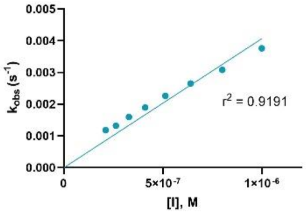	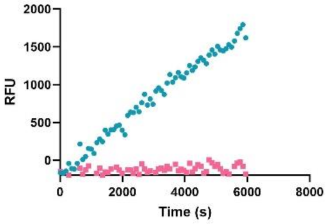

<u>Compound 31b</u>

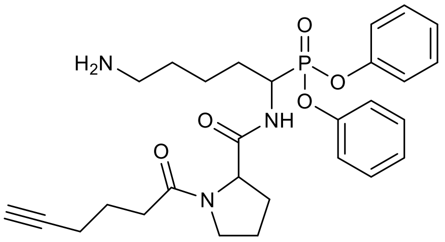

	Progress curve	k_{app} calculation	Jump dilution
**Trypsin-3**<br>**IRREV.**	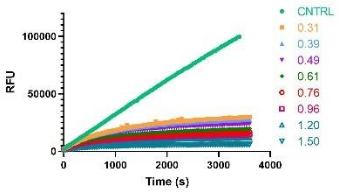	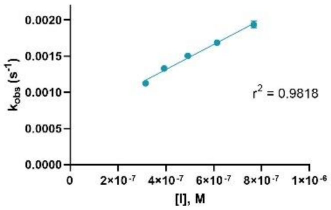	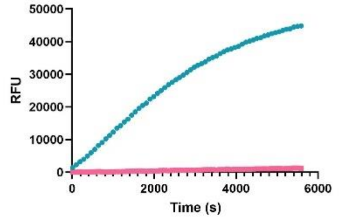
**β-tryptase**<br>**IRREV.**	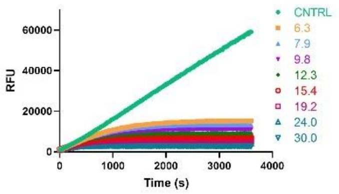	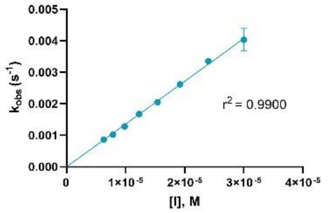	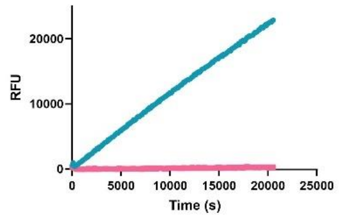
**Thrombin**<br>**IRREV.**	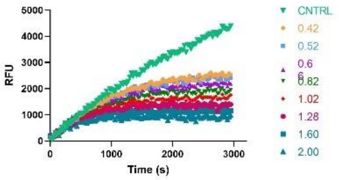	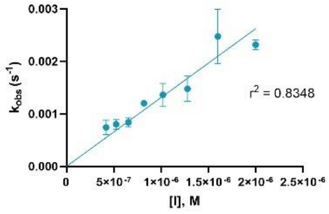	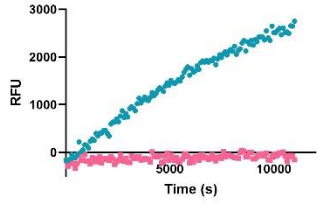
**uPA**<br>**IRREV.**	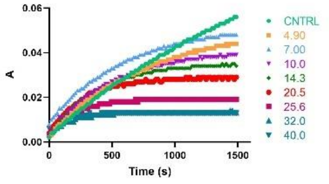	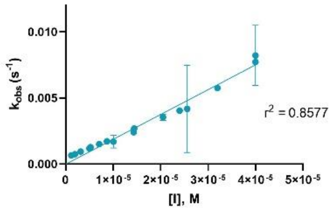	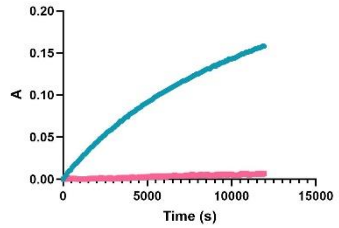
